# Genetic Testing for *APOL1* in Adults With Hypertension: The GUARDD-US Randomized Clinical Trial

**DOI:** 10.1001/jamanetworkopen.2026.0528

**Published:** 2026-03-05

**Authors:** Michael T. Eadon, Kerri L. Cavanaugh, Lilin She, Kady-Ann Steen-Burrell, Dinushika Mohottige, Girish N. Nadkarni, Heather Kitzman, Hrishikesh Chakraborty, Philip E. Empey, Nita A. Limdi, Rajbir Singh, Cherry Maynor Beasley, Alexander S. Parker, Emily J. Cicali, Michelle A. Ramos, Sabrina Clermont, Leilani Dodgen, Erica N. Elwood, Mimsie Robinson, Ebele M. Umeukeje, Abraham Garcia Ortega, Nandini Shroff, Marc B. Rosenman, Khoa A. Nguyen, Simona Volpi, Renee Rider, Paul R. Dexter, Todd C. Skaar, Josh F. Peterson, Larisa H. Cavallari, Julie A. Johnson, Christina M. Wyatt, Lori A. Orlando, Rhonda M. Cooper-DeHoff, Carol R. Horowitz

**Affiliations:** 1 Division of Nephrology, Department of Medicine, Indiana University School of Medicine, Indianapolis; 2 Division of Clinical Pharmacology, Department of Medicine, Indiana University School of Medicine, Indianapolis; 3 Division of Nephrology & Hypertension, Department of Medicine, Vanderbilt University Medical Center, Nashville, Tennessee; 4 Duke Clinical Research Institute, Duke University School of Medicine, Durham, North Carolina; 5 Barbara T. Murphy Division of Nephrology, Department of Medicine, Icahn School of Medicine at Mount Sinai, New York, New York; 6 Department of Population Health Science and Policy, Icahn School of Medicine at Mount Sinai, New York, New York; 7 Institute for Health Equity Research, Icahn School of Medicine at Mount Sinai, New York, New York; 8 The Windreich Department of Artificial Intelligence and Human Health, Icahn School of Medicine at Mount Sinai, New York, New York; 9 The Hasso Plattner Institute for Digital Health at Mount Sinai, New York, New York; 10 The Charles Bronfman Institute for Personalized Medicine, Icahn School of Medicine at Mount Sinai, New York, New York; 11 Peter O’Donnell Jr School of Public Health, University of Texas Southwestern Medical Center, Dallas; 12 Department of Pharmacy & Therapeutics, University of Pittsburgh School of Pharmacy, Pittsburgh, Pennsylvania; 13 Department of Neurology, Heersink School of Medicine, University of Alabama at Birmingham; 14 Meharry Medical College, Nashville, Tennessee; 15 University of North Carolina at Pembroke, Lumberton; 16 University of Florida College of Medicine–Jacksonville, Jacksonville; 17 Department of Pharmacotherapy and Translational Research, Center for Pharmacogenomics and Precision Medicine, University of Florida College of Pharmacy, Gainesville; 18 Baylor Scott & White Health and Wellness Center, Dallas, Texas; 19 Bethel Gospel Assembly, New York, New York; 20 The Institute for Family Health, New York, New York; 21 Department of Pediatrics, Northwestern University Feinberg School of Medicine, Chicago, Illinois; 22 Division of Genomic Medicine, National Human Genome Research Institute, Bethesda, Maryland; 23 Department of Medicine, Indiana University School of Medicine, Indianapolis; 24 Department of Biomedical Informatics, Vanderbilt University Medical Center, Nashville, Tennessee; 25 Department of Medicine, Vanderbilt University Medical Center, Nashville, Tennessee; 26 Center for Precision Medicine, Vanderbilt University Medical Center, Nashville, Tennessee; 27 College of Medicine, The Ohio State University, Columbus; 28 College of Pharmacy, The Ohio State University, Columbus; 29 Clinical and Translational Science Institute, The Ohio State University, Columbus; 30 Division of Nephrology, Department of Medicine, Duke University School of Medicine, Durham, North Carolina; 31 Section of General Internal Medicine, Wake Forest University School of Medicine, Winston-Salem, North Carolina; 32 Department of Medicine, Icahn School of Medicine at Mount Sinai, New York, New York

## Abstract

**Importance:**

Apolipoprotein L1 locus (*APOL1*) high-risk alleles are associated with incidence of chronic kidney disease (CKD) among people with African ancestry. Few studies have examined the effect of genetic return of results on blood pressure (BP) management and control.

**Objective:**

To determine whether providing *APOL1* high-risk genotype results to people with hypertension and their clinicians would reduce systolic BP (SBP) and improve CKD screening and diagnosis.

**Design, Setting, and Participants:**

From July 1, 2020, to September 30, 2023, adults aged 18 to 70 years with hypertension and self-reported African ancestry were enrolled at 14 institutions and 54 clinical sites across the US. Eligible patients either (1) lacked diagnoses of diabetes and CKD or (2) had a diagnosis of CKD with or without diabetes.

**Interventions:**

Participants were randomized to receive *APOL1* genotype results immediately (intervention) or 6 months after enrollment (control). Clinical decision support encouraged appropriate CKD screening, diagnosis, and antihypertensive therapy.

**Main Outcomes and Measures:**

The primary outcome was change in SBP in individuals with *APOL1* high-risk allelles at 3 months, assessed in a modified intention-to-treat analysis. Prespecified per-protocol subgroup analyses included those with uncontrolled BP (baseline SBP ≥140 mm Hg or diastolic blood pressure ≥90 mm Hg), uncontrolled BP while receiving antihypertensive therapy, and CKD at enrollment. Secondary outcomes included urine microalbumin screening and new CKD diagnoses.

**Results:**

Of 6754 individuals recruited (mean [SD] age, 55.3 [10.3] years; 4310 women [63.8%]), 954 (14.1%; mean [SD] age, 54.9 [10.0] years; 600 women [62.9%]) had 2 *APOL1* risk alleles. At 3 months, there was no difference in SBP between the intervention and control groups (between-group difference, −0.3 mm Hg [95% CI, −2.7 to 2.1 mm Hg]). Among 377 individuals with uncontrolled BP, the mean SBP change was −4.1 mm Hg (95% CI, −7.7 to −0.5 mm Hg) more in the intervention group than the control group (*P* = .004). SBP improvement was also observed for the intervention in the subgroup of patients with uncontrolled BP receiving antihypertensive therapy (SPB difference, −4.3 mm Hg [95% CI −8.0 to −0.5 mm Hg]; *P* = .004), but not the CKD subgroup (SPB difference, 0.8 mm Hg [95% CI, −3.0 to 4.5 mm Hg]). Provision of *APOL1* genotype led to increased urine microalbumin screening (between-group difference, 17.3% [95% CI, 9.6%-24.9%]; *P* < .001) and CKD diagnoses (between-group difference, 5.7% [95% CI, 2.2%-9.3%]; *P* = .002) at 6 months.

**Conclusions and Relevance:**

Provision of *APOL1* genotype high-risk results to participants and clinicians was not associated with SBP reduction overall. Among the subset of patients with uncontrolled BP, the intervention group had a significant SBP reduction. *APOL1* disclosure also increased the rate of CKD screening and diagnosis. Effects of reporting *APOL1* genotype merit further investigation among those with uncontrolled BP.

**Trial Registration:**

ClinicalTrials.gov Identifier: NCT04191824

## Key Points

**Question:**

Among participants with hypertension with an apolipoprotein L1 locus *(APOL1*) high-risk genotype, would immediate genetic return of results vs no genetic testing until trial conclusion result in a greater improvement in systolic blood pressure (SBP) at 3 months?

**Findings:**

This randomized clinical trial including 6754 participants found that, at 3 months, there was no difference in SBP between those with immediate and delayed results; however, among participants with uncontrolled blood pressure, those who received immediate results saw a greater reduction in SBP than those with a delay. Provision of *APOL1* genotype also led to increased urine microalbumin screening and chronic kidney disease diagnoses at 6 months.

**Meaning:**

This study suggests that immediate provision of *APOL1* results to participants and clinicians was not associated with SBP reduction, but genotyping increased the rate of CKD screening and diagnosis.

## Teaser

This randomized clinical trial assesses whether immediate provision of *APOL1* high-risk genotype results to people with hypertension and their clinicians reduces systolic blood pressure at 3 months and improves chronic kidney disease screening and diagnosis.

## Introduction

Hypertension and chronic kidney disease (CKD) potentiate the risk for atherosclerotic cardiovascular disease (ASCVD) morbidity and mortality.^1,2,3^ International guidelines recommend blood pressure (BP) control to reduce ASCVD risk in individuals with or without CKD.^4,5^ However, only half of individuals with hypertension achieve recommended BP goals.^6^

Race and ethnicity are sociopolitical constructs, and racial and ethnic disparities in BP control and CKD prevalence are magnified by social determinants of health, which disproportionately affect members of racial and ethnic minority populations, and particularly Black populations.^3,7,8,9^ Ancestry has some genetic underpinnings.^10,11,12^ One in 7 individuals with self-reported African ancestry are homozygous for high-risk alleles (G1 and G2) of the apolipoprotein L1 locus (*APOL1*-HR) associated with protection against sleeping sickness endemic in West and Central Africa.^13,14^ *APOL1*-HR confers increased risk for incident CKD and progression to end-stage kidney disease,^15,16,17,18^ both of which increase ASCVD risk.^19^ *APOL1*-HR is also associated with elevated BP.^20^ BP control is associated with improved cardiovascular mortality among people with self-reported or genetically determined African ancestry and *APOL1*-HR.^21^ CKD remains underdiagnosed,^22^ and international guidelines emphasize enhanced BP control for individuals with CKD to reduce ASCVD risk.^2,23^ Few studies have examined the effect of genetic return of results (gROR) on BP management and control.

A single-center pilot study, conducted in New York City,^20^ demonstrated that *APOL1* gROR to participants and clinicians is feasible and resulted in improved systolic BP (SBP) at 3 months for individuals with *APOL1*-HR compared with those without *APOL1*-HR. Genetic Testing to Understand and Address Renal Disease Disparities Across the United States (GUARDD-US) expands on this pilot to examine the effect of participant and clinician knowledge of *APOL1* genotype on BP among individuals with *APOL1*-HR across 14 institutions and 54 clinical sites.^24^ Using community-engaged approaches, GUARDD-US implemented clinical decision support (CDS) that conveyed patient-specific genotype and phenotype information in the form of best practice alerts or consultation notes and recommended actions such as reaching BP targets, screening for CKD with urine microalbumin testing, or prescribing an angiotensin-converting enzyme inhibitor (ACEI) or angiotensin receptor blocker (ARB) for individuals with proteinuria.^24^ We hypothesized that, among participants with *APOL1*-HR, immediate gROR would result in a greater improvement in SBP at 3 months compared with *APOL1* gROR delayed until after trial conclusion.

## Methods

### Study Design

GUARDD-US was a pragmatic (ie, conducted as part of routine clinical practice), randomized clinical trial that evaluated the effect of *APOL1* genetic testing on BP reduction, with an enrollment period from July 1, 2020, to September 30, 2023.^25,26^ It was approved by a single institutional review board of record, hosted by Duke University. All participants provided voluntary, written informed consent. This study followed the Consolidated Standards of Reporting Trials (CONSORT) reporting guideline. The full trial protocol and statistical analysis plan are included in Supplement 1.

### Participants

We collected self-identified ancestry and race and ethnicity, the latter using the following categories: American Indian, Native American, or Alaska Native; Asian; Black or African American; Hispanic or Latino or Latina; Middle Eastern or North African or Mediterranean; Native Hawaiian or Other Pacific Islander; White or European American, multiple races or ethnicities; and other race or ethnicity, unknown race or ethnicity, or preferred not to answer. Eligible participants were aged 18 to 70 years with documented hypertension and self-reported African ancestry who either (1) lacked diagnoses of diabetes and CKD or (2) had a diagnosis of CKD stage 1 to 4, with or without diabetes. Exclusion criteria included prior *APOL1* genotyping; terminal illness; kidney failure (estimated glomerular filtration rate [eGFR] <15 mL/min/1.73 m^2^); use of a left ventricular assist device; patient-reported pregnancy; a liver, kidney, or allogeneic bone marrow transplant. Participants were recruited from 54 clinical sites in 14 affiliated institutions, including academic, community, and safety-net practices in urban and rural settings.

### Study Procedures and Interventions

Participants were randomized to receive immediate gROR (intervention group) or delayed gROR after study participation concluded (control group). At baseline, all participants underwent standardized BP measurements, completed a survey, provided a DNA sample for genotyping, and received an educational booklet about hypertension, CKD, and *APOL1* testing. At the 3- and 6-month study visits, all participants had repeated BP measurements and surveys including demographic characteristics, medication reconciliation, attitudes about genetics, kidney disease, hypertension, and social determinants. We collected BP measurements during an 8-week study window, either in person (95.6% [6457 of 6754]) or, if not possible, using BPs recorded in the electronic health record (EHR; 4.4% [297 of 6754]). Clinicians received *APOL1* results regardless of genotype, via CDS recommendations (as best practice alerts or EHR consultation notes) that included BP targets, recommendations for urine microalbumin testing, and prescription of an ACEI or ARB to individuals with proteinuria, as previously described.^24^ CDS included recommendations to reach a target BP of less than 140/90 mm Hg and consider ASCVD risk criteria to implement more stringent BP targets when indicated. Results were also integrated into EHRs. Trained research coordinators verbally provided genotype results to all participants; used teach-back to enhance comprehension; and provided a written lay summary of results, a personalized low-literacy educational document, and the opportunity to receive free genetic counseling. The intervention group received gROR by telephone call after the baseline study visit and as soon as results were available. The control group received gROR after the study conclusion. We rerandomized only participants at low risk (without 2 *APOL1*-HR alleles) after study visit 1 at certain sites into a separate pharmacogenomics substudy described elsewhere.^26^

All genotyping was performed in College of American Pathologists–accredited and Clinical Laboratory Improvement Amendments–certified laboratories. A custom polymerase chain reaction–based assay (TaqMan; ThermoFisher) for 3 single-nucleotide variants (rs73885319, rs60910145, and rs71785313) detected the G1 and G2 risk alleles. Study data were collected and managed using REDCap electronic data capture tools.^27^

### Outcomes

The primary outcome was SBP change from baseline to 3 months between groups, assessed in a modified intention-to-treat (mITT) analysis among individuals with *APOL1*-HR (n = 954), who represent a subset of the 6754 individuals randomized to the intervention. Secondary outcomes included appropriate EHR documentation of CKD as a diagnosis and urine microalbumin screening from baseline to 6 months. Another secondary outcome was temporal trend in SBP change over 6 months. Per-protocol subgroup analyses included SBP change from baseline to 3 months among those with uncontrolled BP at enrollment (SBP ≥140 mm Hg or diastolic BP [DBP] ≥90 mm Hg), uncontrolled BP while receiving antihypertensive therapy at enrollment, and CKD. Exploratory outcomes were diastolic BP change, SBP and DBP change 6 months after enrollment, cost-effectiveness (described elsewhere^26^), additional subgroup analyses (age, sex, eGFR, health literacy, educational level, insurance status, socioeconomic status, health care access, prior genetic testing, and medication adherence), and patient-reported measures of genetic testing understanding, satisfaction, and related behaviors.

### Sample Size

Based on an SBP SD of 18.1 mm Hg from a pilot study,^20^ 938 individuals with *APOL1*-HR (469 in each group) were required to detect a difference of 3.5 mm Hg in SBP change from baseline to 3 months between groups with 80% power, a difference similar to that of lifestyle and pharmacologic interventions in CKD^28,29^ that are known to reduce major adverse cardiovascular events.^30^

### Interim Analyses

The data safety and monitoring board reviewed 2 blinded interim analyses in a closed session and found that the study did not fulfill criteria for either efficacy or futility. They determined that to maintain 80% power, enrollment needed to be expanded from 5435 to 6750 due to a lower-than-expected *APOL1*-HR frequency (14.1% [954 of 6754]).

### Randomization and Blinding

We randomized participants 1:1 to intervention or control, stratified by clinical site with block randomization (random block sizes of 4 and 8) within each site. Participants, clinicians, and study personnel were not blinded to randomized treatment assignment because genotype results were available in the EHR for the intervention arm. Investigators were blinded to interim analyses and outcomes during the study.

### Statistical Analysis

We compared the intervention and control groups among participants with *APOL1*-HR (G1/G1, G1/G2, or G2/G2), who comprised the mITT population. We assessed SBP change from baseline to 3 months between groups by the *t* test, using a 2-sided α of .049 to maintain an overall α of .05 accounting for 2 interim analyses. We adjusted the raw *P* value threshold from .05 to .049 using the Lan-Demets spending function approximating O’Brein-Fleming boundaries for the primary outcome. We conducted a covariate-adjusted linear regression analysis to account for differences in baseline characteristics between intervention and control groups, which included randomized treatment as an explanatory factor and baseline SBP as a covariate. Prespecified baseline covariates included CKD, age, and sex. Secondary, subgroup, and exploratory analyses are reported with difference in proportions, least-square mean estimates, and 95% CIs. The widths of the 95% CIs were not adjusted for multiplicity and should not be used to make definitive conclusions of treatment effects. We planned but did not implement missing data analyses because the percentage of missing data was below the prespecified 10% threshold.

We assessed 2 secondary outcomes (urine microalbumin screening and new CKD diagnosis) categorically (yes or no) by the Wald test for equality of proportions and a third outcome (temporal trend in SBP over 6 months) by a covariate-adjusted repeated-measures mixed model. We assessed the 3 per-protocol analytic subgroups for SBP change from baseline to 3 months (those with baseline CKD [eGFR ≤60 mL/min/1.73 m^2^], uncontrolled BP, and uncontrolled BP with active antihypertensive treatment at baseline) by a covariate-adjusted linear regression. We conducted additional exploratory analyses based on age, sex, eGFR, health literacy, educational level, insurance status, poverty level, health care access, prior non-*APOL1* genetic testing, and medication adherence. To control for multiplicity, we applied the Bonferroni method to the secondary analyses and subgroup analyses. The adjusted significance threshold for these analyses was α = .008 (.049/6). Raw *P* values for these analyses are reported against the Bonferroni-adjusted threshold. We did not adjust 95% CIs or additional exploratory subgroup analyses for multiplicity. All statistical tests were 2-sided. The full statistical analysis plan is included in Supplement 1. All analyses were performed using SAS software, version 9.4 (SAS Institute Inc).

## Results

### Participants

**Figure 1.**
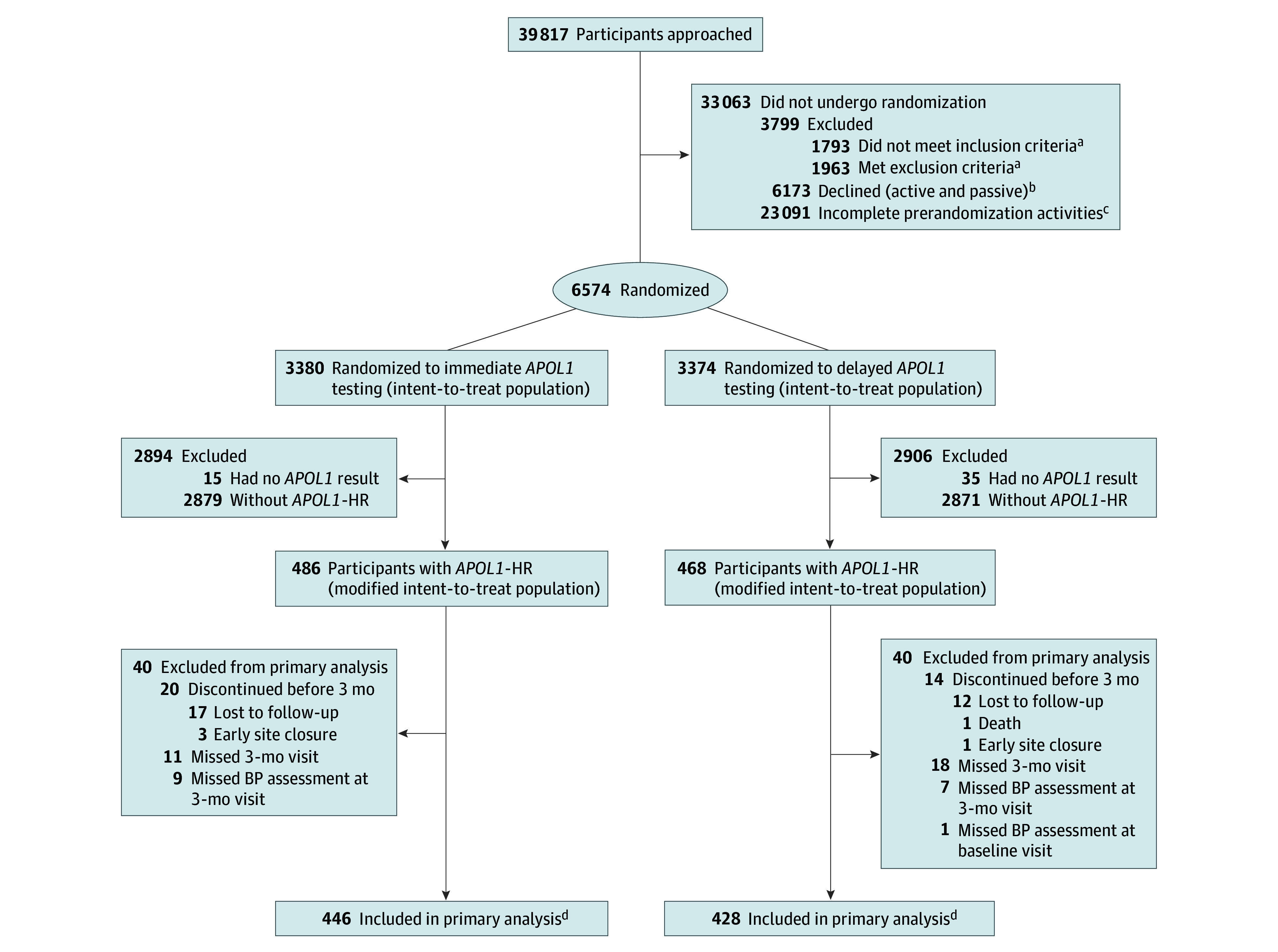
Participant Flow Diagram *APOL1* indicates apolipoprotein L1 locus; *APOL1*-HR indicates *APOL1* high-risk genotype. ^a^Data from 1 site not included due to missing data. ^b^A total of 4991 participants actively declined: 4866 without reading the informed consent form and 125 after reading it. A total of 1182 participants passively declined after reaching the maximum number of contact attempts. ^c^Participants did not attend the baseline visit or could not be reached for scheduling. ^d^The primary analysis focused on the change in systolic blood pressure (BP) from baseline to 3 months between treatment groups. Follow-up continued until 6 months after randomization. In the immediate *APOL1* testing group, 414 participants completed the 6-month visit, while 72 discontinued (65 lost to follow-up, 1 withdrawn by investigators, and 6 due to early site closure). In the delayed *APOL1* testing group, 397 participants completed the 6-month visit, and 71 discontinued (69 lost to follow-up, 1 death, and 1 due to early site closure). Participants missing the 3-month or 6-month visits were included in analyses if an appropriate BP reading was available in the electronic health record.

We enrolled and randomized 6754 participants: 3380 intervention and 3374 control (Figure 1). The mean (SD) age was 55.3 (10.3) years, 4310 participants (63.8%) were female and 2443 (36.2%) were male, the mean (SD) SBP was 133.1 (19.2) mm Hg, 1721 (33.4%) lived below the federal poverty level, and 2585 (38.3%) had a high school education or less (eTable 1 in Supplement 2). All participants self-reported African ancestry: 19 (0.3%) identified as American Indian, Native American, or Alaska Native; 1 (0.01%) as Asian; 6113 (90.5%) as Black or African American; 58 (0.9%) as Hispanic or Latino or Latina; 2 (0.03%) as Middle Eastern or North African or Mediterranean; 4 (0.1%) as Native Hawaiian or Other Pacific Islander; 1 (0.01%) as White or European American, 453 (6.7%) as multiple races or ethnicities; and 102 (1.5%) as other race or ethnicity, unknown race or ethnicity, or preferred not to answer. A total of 96 of 930 participants (10.3%) reported previous genetic testing. Baseline characteristics were balanced between randomization groups. The primary outcome was assessed in an mITT analysis of 954 participants with *APOL1-*HR (14.2%), not all 6754 randomized participants. The 954 participants with *APOL1-*HR were similar to the full cohort and by randomized group, except for having more female participants in the intervention group (326 of 486 [67.1%] vs 274 of 468 [58.5%]) (Table 1). Characteristics overall and by intervention group are presented in eTable 2 in Supplement 2. A total of 192 of 377 participants (50.9%) with uncontrolled BP were prescribed ACEI or ARB therapy at baseline. At baseline, 31.8% of participants (303 of 954) were prescribed 1 antihypertensive medication, 34.1% (325 of 954) were prescribed 2 antihypertensives, and 28.5% (272 of 954) were prescribed 3 or more antihypertensives (eTable 3 in Supplement 2).

**Table 1.** Participant Characteristics by Randomization to Immediate vs Delayed Return of *APOL1* Results

Characteristic	*APOL1* high-risk genotype (n = 954)		Uncontrolled BP at baseline (n = 377)	
	Immediate (n = 486)	Delayed (n = 468)	Immediate (n = 194)	Delayed (n = 183)
Age at randomization, mean (SD), y	54.7 (9.9)	55.1 (10.1)	53.4 (10.2)	53.7 (10.1)
Sex at birth, No. (%)				
Female	326 (67.1)	274 (58.5)	130 (67.0)	103 (56.3)
Male	160 (32.9)	194 (41.5)	64 (33.0)	80 (43.7)
Self-identified race and ethnicity, No. (%)				
American Indian, Native American, or Alaska Native	0	2 (0.4)	0	1 (0.5)
Asian	0	0	0	0
Black or African American	457 (94.0)	422 (90.2)	183 (94.3)	163 (89.1)
Hispanic or Latino or Latina	1 (0.2)	1 (0.2)	0	0
Middle Eastern or North African or Mediterranean	1 (0.2)	0	0	0
Native Hawaiian or Other Pacific Islander	1 (0.2)	1 (0.2)	0	1 (0.5)
White or European American	0	0	0	0
Multiple	17 (3.5)	34 (7.3)	7 (3.6)	14 (7.7)
Other, unknown, prefer not to answer^a^	9 (1.9)	8 (1.7)	4 (2.1)	4 (2.2)
Education: more than high school, No./total No. (%)	273/482 (56.6)	274/467 (58.7)	106/192 (55.2)	104/182 (57.1)
Household income <$40 000/y, No./total No. (%)	213/363 (58.7)	198/374 (52.9)	88/155 (56.8)	82/143 (57.3)
Insurance, No./total No. (%)				
No insurance	35/482 (7.3)	42/467 (9.0)	23/193 (11.9)	26/182 (14.3)
Medicaid^b^	116/482 (24.1)	83/467 (17.8)	39/193 (20.2)	38/182 (20.9)
Medicare^b^	70/482 (14.5)	69/467 (14.8)	23/193 (11.9)	20/182 (11.0)
Medicare and Medicaid	48/482 (10.0)	52/467 (17.8)	13/193 (6.7)	12/182 (6.6)
Private health insurance only	163/482 (33.8)	173/467 (37.0)	73/193 (37.8)	66/182 (36.3)
Other insurance (including military health care or VA)	50/482 (10.4)	48/467 (10.3)	22/193 (11.4)	20/182 (11.0)
Below federal poverty level, No./total No. (%)	126/362 (34.8)	124/372 (33.3)	51/155 (32.9)	54/143 (37.8)
Current tobacco use, No./total No. (%)	109/485 (22.5)	104/467 (22.3)	37/193 (19.2)	47/183 (25.7)
BP medication use at enrollment, No. (%)	463 (95.3)	437 (93.4)	182 (93.8)	169 (92.3)
Baseline SBP, mean (SD), mm Hg	132.5 (18.9)	133.8 (19.5)	148.6 (16.7)	151.1 (17.4)
No.	485	467	194	183
Baseline DBP, mean (SD), mm Hg	83.1 (11.9)	82.8 (13.6)	92.9 (9.9)	93.7 (13.1)
No.	485	467	194	183
SBP ≥140 mm Hg, No./total No. (%)	142/485 (29.3)	143/467 (30.6)	142/194 (73.2)	143/183 (78.1)
DBP ≥90 mm Hg, No./total No. (%)	133/485 (27.4)	121/467 (25.9)	133/194 (68.6)	121/183 (66.1)
SBP <140 and DBP <90 mm Hg, No./total No. (%)	291/485 (60.0)	284/467 (60.8)	NA	NA
Diabetes diagnosis, No./total No. (%)^c^	97/454 (21.4)	106/433 (24.5)	35/184 (19.0)	44/169 (26.0)
CKD diagnosis, No./total No. (%)^d^	146/454 (32.2)	166/433 (38.3)	53/182 (29.1)	72/170 (42.4)

Abbreviations: *APOL1*, apolipoprotein L1 locus; BP, blood pressure; CKD, chronic kidney disease; DBP, diastolic blood pressure; NA, not applicable; SBP, systolic blood pressure; VA, Veterans Administration.

^a^ The “Other” option did not list any specific races or ethnicities.

^b^ Regardless of any additional coverage by private insurance or other insurance.

^c^ Medical record diagnosis.

^d^ Medical record diagnosis, stages 1 to 4.

### Primary BP Outcome

**Figure 2.**
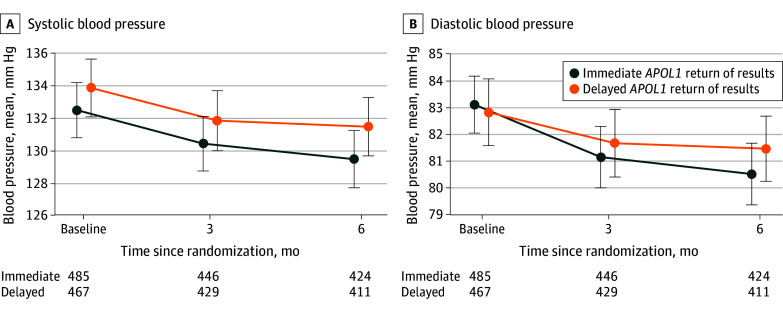
Line Graph of Change in Systolic Blood Pressure and Diastolic Blood Pressure *APOL1* indicates apolipoprotein L1 locus. Error bars indicate 95% CIs.

*APOL1* gROR occurred at a median of 16 days (IQR, 13-22 days), with 99.0% of results (944 of 954) received by 3 months after randomization. In each group, treatment crossover was below 1%, and study completion was similar (intervention, 414 of 486 [85.2%]; control, 397 of 468 [84.8%]). The mean (SD) baseline SBP of the intervention group (132.5 [18.9] mm Hg) and the mean (SD) baseline SBP of the control group (133.8 [19.5] mm Hg) were similar (Figure 2; Table 2). At 3 months, the mean (SD) SBP decreased by 1.8 (18.8) mm Hg in the intervention group and 1.5 (17.3) mm Hg in the control group, a between-group mean difference of −0.3 mm Hg (95% CI, −2.7 to 2.1 mm Hg) (*P* = .78). The mean (SD) covariate-adjusted SBP change from baseline to 3 months did not differ between groups (difference, −0.5 [1.1] mm Hg [95% CI, −2.6 to 1.6 mm Hg]) (Table 2; eTable 4 in Supplement 2). Similar patterns were observed in the DBP change from baseline to 3 months (difference, −0.9 mm Hg [95% CI, −2.2 to 0.5 mm Hg]) and DBP change from baseline to 6 months (difference, −1.3 mm Hg [95% CI, −2.7 to 0.1 mm Hg]). A mixed-model analysis for the trend in change in SBP from baseline to 6 months did not demonstrate a difference between groups (difference, −1.1 mm Hg [95% CI, −2.8 to 0.7 mm Hg]). At baseline, 60.0% of participants in the intervention group (291 of 485) and 60.8% in the control group (284 of 467) had controlled BP (<140/90 mm Hg). At 3 months, 64.6% of participants in the intervention group (288 of 446) and 63.6% of participants in the control group (273 of 429) had a BP less than 140/90 mm Hg. At 6 months, 65.1% of participants in the intervention group (276 of 424) and 62.0% of participants in the control group (255 of 411) had controlled BP. A sensitivity analysis excluded the EHR-derived BPs obtained in 4.4% (42 of 954) of 3-month visits and did not alter the findings (eTable 5 in Supplement 2).

**Table 2.** Primary Outcome and Secondary CKD-Related Outcomes^a^

Outcome	Return of *APOL1* results		Difference between groups (95% CI)
	Immediate (n = 486)	Delayed (n = 468)	
Primary analysis, change in SBP, baseline to 3 mo^b^			
SBP at 3 mo, all participants, mean (SD), mm Hg	130.4 (18.0)	131.8 (19.4)	NA
No.	446	429	NA
Change in SBP at 3 mo, all participants, mean (SD), mm Hg	−1.8 (18.8)	−1.5 (17.3)	−0.3 (−2.7 to 2.1)
No.	446	428	NA
Covariate-adjusted linear regression model of primary outcome, 3 mo^c^			
Change in SBP at 3 mo, all participants, least-square mean (SE)	−1.3 (0.8)	−0.8 (0.8)	−0.5 (−2.6 to 1.6)
No.	446	428	NA
Urine microalbuminuria or proteinuria orders, No./total No. (%)^d^			
Urine microalbuminuria or proteinuria test orders documented at baseline	207/473 (43.8)	221/454 (48.7)	−4.9 (−11.3 to 1.5)
Documented order of microalbuminuria or proteinuria tests by 6 mo	302/473 (63.8)	264/454 (58.1)	5.7 (−0.6 to 12.0)
Change in urine microalbuminuria or proteinuria test orders from baseline to 6 mo	95/473 (20.1)	43/454 (9.5)	10.6 (6.1 to 15.1)
Change in urine microalbuminuria or proteinuria test orders from baseline to 6 mo, among participants with no orders at baseline	95/266 (35.7)	43/233 (18.5)	17.3 (9.6 to 24.9)
Documented diagnosis of CKD (any stage), No./total No. (%)^d^			
Documented diagnosis of CKD (any stage) at baseline	109/456 (23.9)	128/441 (29.0)	−5.1 (−10.9 to 0.6)
Documented diagnosis of CKD (any stage) by 6 mo	140/456 (30.7)	138/441 (31.3)	−0.6 (−6.6 to 5.5)
Change in documented diagnosis for CKD (any stage) from baseline to 6 mo	31/456 (6.8)	10/441 (2.3)	4.5 (1.8 to 7.2)
Change in documented diagnosis for CKD (any stage) from baseline to 6 mo, among participants with no diagnosis at baseline	31/347 (8.9)	10/313 (3.2)	5.7 (2.2 to 9.3)

Abbreviations: *APOL1*, apolipoprotein L1 locus; CKD, chronic kidney disease; NA, not applicable; SBP, systolic blood pressure.

^a^ CKD defined by *International Statistical Classification of Diseases and Related Health Problems, Tenth Revision* codes, estimated glomerular filtration rate, and urine albuminuria or proteinuria tests in the 24 months prior to enrollment; additional detail provided in eTable 7 in Supplement 2.

^b^ Primary outcome of change in SBP was assessed at 3 months by the *t* test.

^c^ Covariate-adjusted mixed model exploratory analysis.

^d^ Secondary CKD-related outcomes were assessed at 6 months.

### Per-Protocol Subgroup Analyses

**Figure 3.**
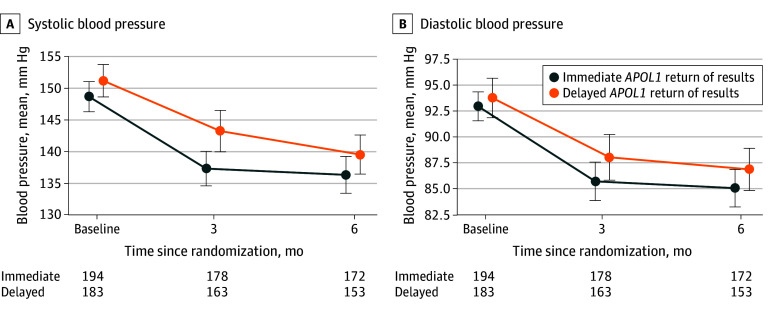
Line Graph of Change in Systolic Blood Pressure and Diastolic Blood Pressure in the Subgroup With Uncontrolled Blood Pressure at Baseline *APOL1* indicates apolipoprotein L1 locus. Error bars indicate 95% CIs.

Per-protocol subgroup analyses were conducted among those with uncontrolled BP or CKD at baseline. The mean (SD) baseline SBP in the subgroup with uncontrolled BP was similar between treatment arms (intervention, 148.6 [16.7] mm Hg; control, 151.1 [17.4] mm Hg). There was a significant interaction between the uncontrolled BP subgroup factor and randomized treatment (*P* = .004 for interaction, below the Bonferroni multiple testing threshold of *P* < .008): the intervention group had a significantly greater mean (SD) SBP change from baseline to 3 months than the control group (−11.4 [18.7] mm Hg vs −7.1 [20.3 mm Hg]) (Figure 3; eTable 4 in Supplement 2). A covariate-adjusted regression analysis yielded a least-square mean difference of SBP change from baseline to 3 months of −4.1 mm Hg (95% CI −7.7 to −0.5 mm Hg) favoring immediate gROR (eFigure 1 and eTable 4 in Supplement 2). The magnitude of SBP reduction was maintained at 6 months (not statistically significant). Individuals with uncontrolled BP receiving antihypertensive therapy at baseline had a similar interaction (difference in SBP change from baseline to 3 months, −4.3 mm Hg [95% CI −8.0 to −0.5 mm Hg]; *P* = .004 for interaction). Baseline CKD diagnosis did not modify the effect of the intervention on SBP change from baseline to 3 months (0.8 mm Hg [95% CI, −3.0 to 4.5 mm Hg]) (eFigure 1 and eTable 4 in Supplement 2). In contrast with the direction of effect in the subgroup with uncontrolled BP, we identified a trend of increasing BP among the intervention subgroup with controlled baseline BP (intervention, 121.7-125.8 mm Hg; control, 122.7-124.8 mm Hg; difference in SBP change from baseline to 3 months, 2.7 mm Hg [95% CI, −0.2 to 5.6 mm Hg]) (eTable 6 in Supplement 2).

To provide context, we examined the interaction between age, sex, baseline eGFR, educational level, self-reported medication adherence, health literacy, health insurance, poverty level, and prior non-*APOL1* genetic testing with the treatment effect of gROR as exploratory analyses. The intervention only held a greater effect on SBP change from baseline to 3 months among individuals with incomes exceeding 125% of the federal poverty level (difference in SBP change from baseline to 3 months, 2.9 mm Hg [95% CI, –1.1 to 6.9 mm Hg]; *P* = .03 for interaction without Bonferroni correction) (eFigure 2 in Supplement 2.

### Secondary Outcomes of CKD Screening

At baseline, the prevalence of urine microalbumin testing within 1 year of study entry was similar in the intervention (207 of 473 [43.8%]) and control (221 of 454 [48.7%]) groups (Table 2). At 6 months, among those without baseline urine microalbumin testing, there were more microalbumin testing orders in the intervention group (95 of 266 [35.7%]) than in the control group (43 of 233 [18.5%]), with a 17.3% between-group difference (95% CI, 9.6%-24.9%; *P* < .001). CKD diagnosis documentation from baseline to 6 months increased more in the intervention group (31 of 347 [8.9%]) than in the control group (10 of 313 [3.2%]), with a difference of 5.7% (95% CI, 2.2%-9.3%; *P* = .002). Among those without CKD at baseline, documentation of CKD diagnoses increased more in the intervention group (24 of 308 [7.8%]) than in the control group (1 of 267 [0.4%]) (eTable 7 in Supplement 2).

### Treatment Fidelity

In the 954 participants with *APOL1*-HR, no change in the number of antihypertensive medications prescribed was observed between groups (eTable 3 in Supplement 2). In the uncontrolled BP subgroup (n = 377) intervention arm, the net proportion of participants with antihypertensive medication added were 3.3% (6 of 181) at 3 months and 10.0% (17 of 170) at 6 months. In the control arm, the net proportion of participants with antihypertensive medication added were 0.6% (1 of 167) at 3 months and 2.0% (3 of 148) at 6 months (eTable 3 in Supplement 2). Antihypertensive additions were distributed across multiple medication classes.

Among the 954 participants with *APOL1*-HR, we observed no difference in self-reported nonadherence between intervention (195 of 467 [41.8%]) and control (181 of 444 [40.8%]) arms. There were few differences between groups related to physical activity or dietary changes (eTable 8 in Supplement 2). In the intervention arm, 229 of 412 participants (55.6%) reported greater physical activity and 255 of 411 participants (62.0%) reported a healthier diet. In the control arm, 208 of 395 participants (52.7%) reported greater physical activity and 229 of 396 participants (57.8%) reported healthy diet changes.

### Patient-Reported Outcomes

Participants found the *APOL1* information provided by research coordinators sufficient (821 of 856 [95.9%]) and easy to understand (789 of 857 [92.1%]), and most would get tested again (414 of 440 [94.1%]) (eTable 8 in Supplement 2). Only 7 of 954 participants with *APOL1*-HR (0.7%) expressed distress related to the gROR. All participants were offered to meet in person or virtually with a genetic counselor at no cost to them, and only 5 of 954 (0.5%) chose this option.

## Discussion

The GUARDD-US study results supported the null hypothesis, demonstrating that immediate provision of *APOL1-*HR genotype results to participants and clinicians did not improve SBP. Among a prespecified subgroup of participants with elevated BP at baseline, immediate *APOL1-*HR gROR resulted in a −4.1 mm Hg (95% CI, −7.7 to −0.5 mm Hg) greater SBP change from baseline to 3 months compared with those with delayed gROR, a finding that may prove important and should be explored in future studies. A similar improvement in SBP change from baseline to 3 months was observed among those with uncontrolled baseline BP receiving antihypertensive therapy, but not among a CKD subgroup that included a mix of participants with controlled and uncontrolled BP. Only 39.5% (n = 377) of participants with *APOL1*-HR had a SBP higher than 140 mm Hg or a DBP higher than 90 mm Hg. The lower mean baseline SBP of the GUARDD-US population (133.1 mm Hg) may, in part, explain the negative results compared with other studies of uncontrolled BP among populations with African ancestry.^31^ For individuals with controlled SBP, clinicians and patients may not have deemed further BP reduction necessary, given the lack of guideline uniformity about reaching a target SBP of 120 or 130 mm Hg in general, and among individuals with *APOL1*-HR.^32,33^

SBP increased at 3 months in both study arms for those with controlled BP (intervention, 121.7-125.8 mm Hg; control, 122.7-124.8 mm Hg), yielding a nonsignificantly greater SBP change from baseline to 3 months in the intervention arm (covariate-adjusted increase, 2.7 mm Hg [95% CI, −0.2 to 5.6 mm Hg; *P* = .07). Still, their BPs remained well controlled (<130 mm Hg for most participants). Although only 0.7% reported distress from their *APOL1*-HR gROR, we cannot exclude an effect from *APOL1*-HR gROR contributing to increased anxiety and SBP, or a labeling effect on white-coat hypertension, which some (but not all)^34^ studies have demonstrated.^35,36^

Immediate *APOL1* gROR led to significant improvements in urine microalbumin screening for CKD and in CKD EHR diagnosis documentation. CKD is an ASCVD risk criteria for which the American Heart Association recommends more stringent BP targets.^5^ Increased CKD recognition may contribute to the positive effect of gROR observed in the subgroup with uncontrolled BP. Pragmatic trial structures are suboptimal for mechanistic inference; however, we did collect medication data and participant-reported outcomes by survey and medication review of pill bottles with participants. GUARDD-US provided gROR to both clinicians (who prescribe) and participants (who control diet and exercise). CDS alerts prioritized recommendations for ACEI or ARB therapy; however, 50.9% of patients with uncontrolled BP (192 of 377) were already prescribed these medications at baseline. In the subgroup with uncontrolled BP, we observed intensification of antihypertensive therapy, which was distributed across medication classes. Survey data suggested increased attention to diet and physical activity in the *APOL1*-HR intervention arm, but we used very brief measures, and future studies should assess the effects of diet and physical activity in more detail to draw conclusions about any effect of gROR.

The methods for testing and gROR were well received by participants. We created GUARDD-US educational and gROR materials in collaboration with our expert community partners, which we adapted based on formative research and results of the GUARDD pilot. This may explain why more than 95% of GUARDD-US participants reported satisfaction with gROR and few participants elected to meet with a genetic counselor. Survey data from the GUARDD pilot study also suggested that knowledge of *APOL1* status countered harmful stereotypes that hypertension control among Black individuals primarily relates to medication nonadherence or low health literacy.^37^

### Strengths and Limitations

GUARDD-US has some strengths, including the potential for generalizability, low refusal rates (15.5%), high retention (92%) and satisfaction rates, participant sociodemographic and geographic diversity, and implementation across safety-net, academic, and community clinics; health systems; and EHRs. GUARDD-US also has some limitations. Knowledge of genetic risk is only 1 factor affecting BP; effects of harmful social determinants, which disproportionately affect Black communities, contribute to suboptimal BP control.^38,39,40^ Although this is the largest intervention trial among those with *APOL1*-HR, the mITT analysis is not fully protected by randomization, and there may be unrepresented factors that contribute to residual confounding despite our rigorous statistical methods. The mITT analysis was designed to include participants with 2 *APOL1*-HR alleles. We did not study monoallelic carriers who we now know possess an intermediate phenotype for CKD risk.^41^ Patients with uncontrolled BP were not randomized but rather were selected as a per-protocol population. *APOL1*-HR gROR was delivered with CDS, and we could not separate the effects of gROR and CDS. Finally, we reported data on the effect of gROR on urine albumin-creatinine ratio orders, not completion of UACR testing.

## Conclusions

Our large multicenter randomized clinical trial enrolled 6754 individuals of African ancestry, demonstrating significant engagement with high satisfaction from gROR. *APOL1*-HR gROR to clinicians and participants led to improved SBP among individuals with uncontrolled BP, which merits further investigation for future trials. It also led to increased testing for and diagnosis of CKD, which is important given that that CKD is significantly undertested for and underdiagnosed in primary care. Although consensus indications for clinical *APOL1* genetic testing have not been formalized,^42^ GUARDD-US results corroborate findings that individuals with self-identified African ancestry and elevated BP, as well as their clinicians, may use knowledge of the *APOL1* genotype to improve BP control.^20,37^

## Notes

Supplement 1.
Trial Protocol and Statistical Analysis Plan

Supplement 2. **eAppendix 1.** GUARDD-US Clinical Groups **eAppendix 2.** GUARDD-US Protocol Implementation Teams **eAppendix 3.** GUARDD-US Site PIs and Site Study Staff **eAppendix 4.** Additional Collaborators **eTable 1.** Participant Characteristics of ITT Population **eTable 2.** Participant Characteristics of *APOL1*-HR, *APOL1*-HR With Uncontrolled BP and *APOL1*-HR With Controlled BP **eTable 3.** Description of Antihypertensive Medication Use at Enrollment, 3 Months, and 6 Months mITT and Uncontrolled BP **eTable 4.** Blood Pressure Outcomes at 3 Months and 6 Months, *APOL1*-HR Population **eTable 5.** Sensitivity Analysis of Primary Outcome With Removal of EHR-Derived Measures **eTable 6.** Controlled and Uncontrolled Systolic Blood Pressure Analysis **eTable 7.** Secondary Chronic Kidney Disease-Related Outcomes at 6 Months **eTable 8.** Perspectives of Trial Participants on Sharing Genotype Results and Self-Reported Behaviors **eFigure 1.** Per-Protocol Subgroup Analyses of Treatment Group Difference in Systolic Blood Pressure Change From Baseline to 3 Months **eFigure 2.** Exploratory Subgroup Analyses of Treatment Group Difference in Systolic Blood Pressure Change From Baseline to 3 Months

Supplement 3. **Nonauthor Collaborators.** Members of the Implementing Genomics in Practice (IGNITE) Pragmatic Trials Network

Supplement 4.
Data Sharing Statement
