# Supplementary material for: Genetic Testing for APOL1 in Adults With Hypertension: The GUARDD-US Randomized Clinical Trial
Source: JAMA Netw Open. 2026 Mar 5;9(3):e260528. doi: 10.1001/jamanetworkopen.2026.0528 (PMC12964156; doi:10.1001/jamanetworkopen.2026.0528)
Supplement: Supplement 1. — Trial Protocol and Statistical Analysis Plan [file jamanetwopen-e260528-s001.pdf]

Genetic testing to Understand and Address Renal Disease Disparities across the United States (GUARDD-US)

Principal Investigator: Carol R. Horowitz, MD, MPH

Co-Investigator: Girish Nadkarni, MD

Statistical Investigator: Hrishikesh Chakraborty, DrPH

Clinicaltrial.gov #: NCT04191824

Funded by: National Human Genome Research Institute

Version Number: v6.0

19 March 2024

PROTOCOL VERSION AND AMENDMENT TRACKING

Version Number/ Affected Section(s)	Summary of Revisions Made	Version Date
1/ Protocol Version and Amendment Tracking; Investigator Statement; Abbreviations; Protocol Synopsis; Sections 4,5,6,7, 8, 9, 10 and Appendix B	• Protocol Version and Amendment Tracking – added Amendment 1 change information<br>• Investigator Statement – updated protocol version date<br>• Abbreviations – removed reference to PROMIS and PROPr<br>• Protocol synopsis – number of recruitment sites, inclusion criteria definition of CKD and exclusion criteria definition of terminal illness<br>• Section 4 – Study Design – explain randomization plan<br>• Section 5 – inclusion criteria to clarify CKD definition and exclusion definition of terminal illness<br>• Section 6 – clarified information on EHR queries, provider consent, use of recruitment script as optional, changed will to may and should, clarified consenting process and timeline for completing the baseline visit, and clarified compensation<br>• Section 7 – clarified education materials and APOL1 results, clarified the recommendation for drug class, clarified baseline assessment completion, specimen collection guidelines, added secure email as method of return of results<br>• Section 8 – added information on reportable events and removed meeting location<br>• Section 9 – updated cost effective analysis plan<br>• Section 10 – added pulse to data collection points<br>• Section 15 – Appendix B – clarified recommendation section for poor/normal/rapid metabolizers	05February2020
2/ Protocol Version and Amendment Tracking; Investigator Statement; Protocol	• Protocol Version and Amendment Tracking – added Amendment 2 change information	22June2020

Synopsis; Sections 6,7, and 9	• Investigator Statement – updated protocol version date<br>• Protocol Synopsis – changed analysis method from Mann-Whitney to t-test under main study analyses<br>• Section 6 – Recruitment and Enrollment Procedures – added that "the consenting process can be remote, via phone or electronically if approved by the reviewing IRB".<br>• Section 7 – Study Procedures – added that baseline survey and follow-up surveys may be administered over the phone.<br>• Table 5 – changed "blood draw" to "sample collection"<br>• Section 9 – changed "Mann-Whitney" to 't-test" in Analysis of Primary Endpoint and Other Planned Analyses sections	
3/ Title Page; Protocol Version and Amendment Tracking; Investigator Statement; Protocol Synopsis; Sections 3, 5,7, 8, 9, 10 and 11	• Title page updates<br>• Protocol Version and Amendment Tracking – added Amendment 3 change information<br>• Investigator Statement – updated protocol version date<br>• Protocol synopsis – added in-person as option for return of PGx results, added new exclusion criteria (LVAD), clarified population for primary endpoint analysis, updated duration of participation for EHR data retrieval<br>• Section 3 – Primary Endpoint – clarified population for primary endpoint analysis<br>• Section 5 – Exclusion Criteria – added new exclusion criteria (LVAD)<br>• Section 7 – Intervention/Treatment – clarified distribution methods for written test results and added in-person for verbal communication of test results<br>• Section 7 – Assessments – updated criteria for which blood pressure reading would activate the elevated blood pressure protocol, clarified the thresholds for the elevated BP protocol, updated screen failure definition, updated the guidance for which EMR	27August2021

	BP values would qualify for inclusion in the study database<br>• Section 7 – Data from EMR – revised the % of participants that would be included in the first data extract, updated the timing for the final EHR extract, updated description for how the EHR download and transfer process instructions would be distributed<br>• Section 7 – Clinical Decision Support – updated the description for how the CDS alert details will be distributed<br>• Section 8 – Safety Assessment - revised the % of participants that would be included in the first data extract, updated the timing for the final EHR extract, updated description for how the EHR download and transfer process instructions would be distributed and added virtual as a meeting format<br>• Section 9 – Statistical Analysis Plan – updated language describing the planned interim analyses.<br>• Section 10 – Data management – Updated language regarding verification of laboratory results, revised the % of participants that would be included in the first data extract, updated the timing for the final EHR extract, updated description for how the EHR download and transfer process instructions would be distributed<br>• Section 11 – Updated language under the confidentiality and privacy section regarding participant name disclosure.	
4 Title Page; Protocol Version and Amendment Tracking; Investigator Statement; Protocol Synopsis; Sections 3, 5,7, 8, 9, 10 and 11	• Title page and Investigator Statement<br>• Protocol Version and Amendment Tracking – Changed “Approval date” to “Version Date”, added Amendment 3 change information<br>• Protocol Synopsis – updated number of participants under randomized population, added “~” to “6 months” for duration of participation and clarified inclusion criteria – changed “African American/Black” to “self-reported African ancestry”	10Jun2022

	• Section 4 – Study Design – added language regarding manual blood pressure collection. Updated numbers for randomized population, *APOL1* positive assumed percentage, numbers of population that would be in each arm for main study and PGx substudy. Changed power for PGx substudy from 80% to 90%.<br>• Section 5 – Study Population – updated/clarified inclusion criteria – changed “African American/Black” to “self-reported African ancestry”<br>• Section 6 – Recruitment and Enrollment Procedures – added “Study staff may also ask current participants if they know anyone who would qualify for the study and provide them with study-related contact information to share with others.”<br>• Section 6.3 Screening Procedures – added “Screening may also be performed in a location agreed upon between a potential participant and the research team.”<br>• Section 6.4 Participant Consent Process – added “also” after “The consenting process can”.<br>• Section 6.10 Compensation to Participants – changed “or” to “and/or” in sentence “Participants may be reimbursed for travel or parking expenses per institutional specific policies.”<br>• Section 7 – Study Procedures - Section 7.2 Baseline Assessments – added “which may occur in a location agreed upon between potential participants and the research team,” after “At baseline”.<br>• Section 7.2 Baseline Assessments – added “Manual blood pressures may be collected if necessary.”<br>• Section 7.3 Follow-Up Assessments – added “in a location agreed upon between the participant and the research team” after “Participants enrolled in the study will meet with Research Coordinators”.	

	• Section 7.7 Data Collection from the Electronic Health Record – added "for Other Planned Analyses including" after "variables needed". Deleted "in the" before "Cost Effectiveness Analysis".<br>• Section 7.10 Schedule of Activities – updated Tables 6 and 7 to reflect change in 3M and 6M study visit windows from "+6 wks" to "+8 wks".<br>• Section 9 – Statistical Analysis Plan and Sample Size - Section 9.1 Sample Size Determination – updated numbers for randomized population, *APOL1* positive assumed percentage, numbers of population that would be in each arm for main study and PGx substudy. Updated Tables 8 and 9. Changed power for PGx substudy from 80% to 90%.<br>• Section 9.1 Sample Size Determination – added "Enrollment may end prior to the 6650 target if the study team notes that the necessary 931 *APOL1* positive individuals have already been enrolled."<br>• Section 9.7 Interim Analyses – added language describing how additional interim analyses would be handled if requested by the DSMB.<br>• Sections 6, 7 &10 – removed "to be" from "to be provided in the MOP".	
5 Title Page; Protocol Version and Amendment Tracking; Investigator Statement; List of Tables; Protocol Synopsis; Sections 2, 3, 4, 5, 7, 8, 9, 10 and 11	• Title Page and Investigator Statement<br>• Protocol Version and Amendment Tracking – added Amendment 5 change information<br>• List of Tables – removed Tables 8 & 9<br>• Protocol Synopsis – changed "Appropriate" to "Documented" under objectives and secondary endpoints; clarified PGx substudy participants by including information regarding participating sites; changed randomized population to 6,750; clarified inclusion criteria "allogenic" as added before bone marrow (previously approved on 04-Nov-2022 in clarification memo #4); added "an	19Mar2024

	overall" under Statistical Analyses; changed "All" to "Major" and removed "and secondary" under Substudy Analyses.<br>• Section 2 – Introduction – changed "≤" to "<" and changed "appropriate" to "documented" for CKD diagnosis.<br>• Section 3.2 – Secondary Endpoints – Changed "appropriate" to "documented" under secondary endpoints 2, 3, 4, 5, and 6.<br>• Section 4 – Study Design – clarified PGx substudy population by including information regarding participating sites; updated numbers for randomized population, *APOL1* positive assumed percentage, and numbers of population that would be in each arm for main study and PGx substudy and clarified rationale for updated target; added "approximately" before 90% power.<br>• Section 5 – Study Population – clarified exclusion criteria by adding "allogeneic". (previously approved on 04-Nov-2022 in clarification memo #4)<br>• Section 7.3 – Follow-Up Assessments – changed "6" to "8" consistent with extended window approved in Jul 2022.<br>• Section 7.7 – Data Collected from the Electronic Health Record – changed "in the 6 months" to "up to 8 months" (previously approved on 14-Feb-2024 in clarification memo #5)<br>• Section 7.7 – Data Collected from the Electronic Health Record and Section 8 – Safety Assessments and Monitoring – removed "two", "one" and "another" from the EHR downloads sentence. (previously approved on 14-Feb-2024 in clarification memo #5)<br>• Section 9 – Statistical Analysis Plan and Sample Size – updated numbers for randomized population, *APOL1* positive assumed percentage, numbers of population that would be in each arm for main study and PGx substudy; added additional information regarding continued enrollment until effect size is reached; removed sentences	

	"Additional calculations using the same assumptions…and BP standard deviation"; removed table 8; removed "interim analysis" from PGx substudy; added "approximately" before 90% power for PGx substudy; removed sentences "Additional calculations using the assumptions…major cardiac outcomes"; removed table 9; clarified population for PGx substudy; added "overall" before two-sided for primary endpoint analysis; changed "appropriate to "documented" for secondary endpoint analysis; updated potential covariates by adding "and baseline CKD" and removing "income, education, marital status, and insurance, and Charlson comorbidity index"; updated "above 140 mm Hg" to "140 mm Hg or greater"; added "depending on the outcome of the main study" and removed "body mass index" from Cost-Effectiveness section; removed "all" form pGx substudy analyses section; added "Impact of Social Determinants of Health" under Other Planned Analyses; updated Interim Analyses section to describe a second interim analysis and impact on p-value.<br>• Section 10 – Data Management – Clarified definitions of baseline and follow-up SBPs; Updated CPT codes; added "OR the presence of urine microalbuminuria/proteinuria lab test order and/or result"; removed "AND order of urine microalbuminuria/proteinuria test at 6 months"; updated ICD-10 codes; removed "AND appropriate diagnosis at 6 months (as described in 3b)) under 4 and 6"; added "YN" under 2, 4 and 6; removed "OR having hypertension in the patient's medical record problem list in the 24 months prior to baseline] and [having 2 lab results of eGFR <=60 ml/min 3 months apart with at least one test in the 24 months prior to baseline OR order of urine microalbuminuria/proteinuria test with	

	both eGFR and urine tests in the time period from 24 months prior to baseline to 6 months after baseline]" from 3 and 5; changed "appropriate" to "documented" under 2, 3, 4, 5, and 6.<br>• Section 11 – Ethical and Human Subjects Considerations – changed "recruiters at each site" to "the study team" and changed "and" to "or" in the phrase "de-identified and anonymized".	

INVESTIGATOR STATEMENT

By signing below, I agree to the conditions relating to this trial as set out in this protocol (v6 dated 19Mar2024).

I agree to conduct this clinical trial according to Good Clinical Practice (ICH GCP) and US Regulatory Requirements.

I fully understand that any changes instituted by me without previous discussion with the IGNITE Pragmatic Trials Network Coordinating Center or their designated representative constitute a violation of the protocol.

I agree to adhere to the protocol in all circumstances other than where necessary to protect the well-being of the participant.

Principal Investigator's

Name: ___________________________________

Signature: __________________________ Date____________

ABBREVIATIONS

AA	African Americans
AE	Adverse Event
ACE	Angiotensin-converting enzyme
ARB	Angiotensin receptor blocker
APOL1	Apolipoprotein L1
BP	Blood pressure
CC	Coordinating center
CKD	Chronic kidney disease
CDS	Clinical decision support
CE	Cost-effectiveness
CEA	Cost-effectiveness analysis
CIOMS	Council for International Organizations of Medical Sciences
CLIA	Clinical Laboratory Improvement Amendments
CPT	Current Procedural Terminology
CRF	Case Report Form
DSMB	Data and safety monitoring board
eGFR	Estimated glomerular filtration rate
EHR	Electronic Health Record
ESRD	End stage renal disease
GUARDD-US	Genetic Testing to Understand and Address Renal Disease Disparities across the US
HIPAA	Health Insurance Portability and Accountability Act of 1996
HTN	Hypertension
IGNITE PTN	Implementing Genomics in Practice Pragmatic Trials Network
MOP	Manual of operations
NHGRI	National Human Genome Research Institute
PCT	Pragmatic Clinical Trial
PI	Principal Investigator
PGx	Pharmacogenetic
PPC	Publications and Presentations Committee
ROR	Return of results
SAP	Statistical analysis plan
SBP	Systolic blood pressure
SOP	Standard operating procedure

LIST OF FIGURES

Figure 1. GUARDD-US study design for sites participating in PGx. 23
Figure 2. GUARDD-US study design for sites not participating in PGx. 24

LIST OF TABLES

Table 1. APOL1 Laboratory Report Language 32
Table 2. Genes and gene variants to be tested in the PGx substudy. 33
Table 3. PGx genotypes, phenotypes, and predicted genotype frequencies. 33
Table 4. PGx recommendations[a] 34
Table 5. Schedule of participant activities up to randomization for sites. 38
Table 6. Schedule of activities following participant baseline visit for sites participating in the PGx substudy. 39
Table 7. Schedule of activities following participant baseline visit for sites participating not in the PGx substudy. 40
Table 8. ICD10 diagnosis codes and descriptions for diabetes 56

TABLE OF CONTENTS

1. PROTOCOL SYNOPSIS 16
2. INTRODUCTION 20
2.1 Background, Significance, and Rationale 20
2.2 Aims and Objectives of the Study 21
3. ENDPOINTS 22
3.1 Primary Endpoint 22
3.2 Secondary Endpoints 22
3.3 Pharmacogenetic (PGx) Substudy Endpoints 22
4. STUDY DESIGN 23
4.1 Study Arms & Design 23
4.2 Randomization 24
4.3 Blinding 25
4.4 Sample Size 25
5. STUDY POPULATION 26
5.1 Inclusion Criteria 26
5.2 Exclusion Criteria 27
6. RECRUITMENT AND ENROLLMENT PROCEDURES 28
6.1 Provider Recruitment, Assent, and Consent 28
6.2 Participant Recruitment Strategies 28
6.3 Screening Procedures 28
6.4 Participant Consent Process 29
6.5 Participant Discontinuation/Withdrawal from the Study 29
6.6 Lost to Follow-Up 29
6.7 Risk 29
6.8 Benefit 30
6.9 Costs to the Participants 30
6.10 Compensation to Participants 30
7. STUDY PROCEDURES 31
7.1 Interventions/Treatments 31
7.2 Baseline Assessments 34
7.3 Follow-Up Assessments 35
7.4 Specimen Collection 35

7.5 Specimen Transfer and Genetic Testing Procedures 36
7.6 Return of Genetic Results 36
7.7 Data Collection from the Electronic Health Record 36
7.8 Clinical Decision Support System 37
7.9 Provider Surveys 37
7.10 Schedule of Activities 37
8. SAFETY ASSESSMENT AND MONITORING 41
8.1 Distress from Return of Results 41
8.2 Events of Interest 41
8.3 Safety Monitoring/Data and Safety Monitoring Board 41
8.4 Early Termination and Participant Discontinuation 41
9. STATISTICAL ANALYSIS PLAN AND SAMPLE SIZE 42
9.1 Sample Size Determination 42
9.2 General Statistical Methods 43
9.3 Population for Analyses 43
9.4 Analysis of the Primary Endpoint 43
9.5 Analysis of the Secondary Endpoints 43
9.6 Other Planned Analyses 43
9.7 Interim Analyses 45
9.8 Handling of Missing Data 46
9.9 Multiplicity 46
10. DATA MANAGEMENT 47
10.1 Data Entry and Record Keeping 47
10.2 Data Element Definitions 47
10.3 Database Management and Quality Control of Data 49
10.4 Study Close Out 49
11. ETHICAL AND HUMAN SUBJECTS CONSIDERATIONS 50
11.1 Institutional Review Board/Ethics Committee Review 50
11.2 Use and Disclosure of Protected Health Information 50
11.3 Confidentiality and Privacy 50
11.4 Publication and Data Sharing Policies 50
12. PROTOCOL DEVIATIONS 52
13. CONFLICT OF INTEREST 53

14. REFERENCES 54
15. APPENDICES 56
15.1 Appendix A. Diabetes Diagnosis Codes 56
15.2 Appendix B. PGx Substudy Gene Description and Evidence 76

1. PROTOCOL SYNOPSIS

**Protocol Title**	Genetic testing to Understand and Address Renal Disease Disparities across the United States (GUARDD-US)
**Product/Intervention**	Immediate versus delayed return of Apolipoprotein L1 (*APOL1*) gene testing results to provider and participant.
**Objectives:**	The primary aim is to determine the effect of participant and provider knowledge of a positive *APOL1* status on change in systolic blood pressure (SBP) from baseline to 3 months after randomization. Secondary aims are to:<br>1. Determine the effect of participant and provider knowledge of a positive *APOL1* status on the probability of documented chronic kidney disease (CKD) diagnosis.<br>2. Determine the effect of participant and provider knowledge of a positive *APOL1* status on the probability of receiving a urine test for CKD.<br>3. Explore cost effectiveness, mediators, moderators, psychobehavioral impact of results disclosure on participants, and effects of knowledge of *APOL1* status on provider treatment recommendations.<br><br><u>PGx Substudy:</u><br>GUARDD-US will include a substudy to determine the effect of knowledge of genetic test results that predict efficacy of various antihypertensive medications on change in SBP from baseline to 3 months in *APOL1* negative individuals.
**Study Design**	<u>Study Design:</u><br>GUARDD-US is a prospective, multicenter, unblinded, two arm randomized pragmatic clinical trial. Participants will be randomized in a 1:1 ratio to immediate *APOL1* gene testing and return of results (ROR) to participant and provider (Intervention arm) versus delayed *APOL1* gene testing and ROR to participant and provider (Control arm). The main study will compare outcomes between *APOL1* positive participants in the Intervention arm (i.e., early knowledge of *APOL1* status) to *APOL1* positive participants in the Control arm (i.e., delayed knowledge of *APOL1* status). Participants that are *APOL1* negative in the Intervention and Control groups will not be included in the main study analyses.<br><br><u>Pharmacogenetic (PGx) Substudy Design:</u><br>GUARDD-US will also include a substudy that randomizes participants in the Intervention arm who are from the PGx substudy participating sites and who test negative for *APOL1* to PGx Intervention (i.e., immediate PGx ROR) and PGx Control (i.e., delayed PGx ROR) in a 1:1 ratio. This substudy will compare outcomes between participants in the PGx Control group and the PGx Intervention group.
**Rationale for Study Design**	High-risk variants in the *APOL1* gene explain approximately 70% of the excess prevalence of CKD in African Americans (AAs), conferring a 5 times higher risk for hypertensive CKD and a 10 times higher risk for ESRD. A recent pilot study (GUARDD), showed that returning *APOL1* gene test results had a statistically significant improvement in SBP at 3 months when comparing *APOL1* positives to *APOL1* negatives who received their genetic testing results and when comparing *APOL1* positives that received their results early to overall controls who did not receive their results until after the 3 month visit. GUARDD was not however powered to evaluate the effects of having and knowing a positive *APOL1* status on outcomes for those with high risk of developing CKD (i.e., comparing outcomes for

	APOL1 positive patients who know their genetic risk to *APOL1* positive patients who do not know their genetic risk). A broader trial is needed to better determine the importance of *APOL1* gene testing for improving the testing, diagnosis, and treatment of individuals at risk of CKD.<br><br><u>PGx Substudy:</u><br>New data show that genetic differences may cause patients to respond differently antihypertensive medication therapy, particularly in AAs. Pharmacogenomics may help guide initial or add-on antihypertensive therapy management. However, the impact of PGx testing on BP has not been studied in clinical trials among general or AA populations.
**Study Population**	<u>Randomized Population</u>:<br>Approximately 6,750 participants of African ancestry age 18-70 with hypertension that either: 1) do not have diabetes and do not have CKD, or 2) have CKD (with or without diabetes).<br><br><u>Population for Main Study</u>:<br>Participants from Randomized Population (above) who test positive for *APOL1*<br><br><u>Population for PGx Substudy:</u><br>Participants from Randomized Population (above) randomized to Intervention and who test negative for *APOL1.* Only participants from PGx substudy participating sites are included in this population.
**Number of Recruitment Sites**	~46-60
**Duration of Participation**	~6 months for study visits, up to 12 months for EHR data retrieval
**Description of implementation of intervention (e.g., dose, schedule, etc.)**	The primary intervention for GUARDD-US is the immediate return of *APOL1* genetic testing results to participants and participant providers. Return of *APOL1* genetic testing results to participants and providers will occur as described in Section 7.6. In addition to verbal ROR, participants will also receive lay explanations in writing and an educational booklet explaining their results and genetic risks. Providers will be notified of participant results via standard site-specific laboratory return of results methods and, where possible, a clinical decision support system (CDS) within the electronic health system as described in Section 7.8. Providers may also receive links for accessing provider and participant educational materials through the CDS or other means. Finally, all participants are given the option to discuss their results with a genetic counselor.<br><br>The intervention for the PGx substudy is the immediate return of PGx testing results to participants via phone and/or in-person, return of results and alerts via standard site-specific laboratory return of results methods and a CDS within the electronic health system for providers, and educational materials for both patients and providers regarding the genes tested and phenotypes associated with the efficacy of hypertension medications.
**Inclusion/Exclusion Criteria**	<u>Inclusion Criteria:</u><br>● Self-reported African ancestry<br>● English Speaking

- Age 18-70 years
- Have diagnosis of hypertension
  - Diagnosis of hypertension is defined by either:
    - ICD10 diagnosis codes (i.e., I10; I11.x; I12.x; I13.x; I16.x) OR
    - On active antihypertensive therapy for indication of hypertension OR
    - Having systolic blood pressure of 140 mm Hg or greater in at least 2 of the last 3 consecutive recorded values in the EHR OR
    - Having hypertension in the patient's medical record problem list
- Have been seen at ≥1 time in past year at a participating primary care site
- Either: 1) do not have diabetes and do not have CKD, or 2) have CKD; Participants with diabetes may be included as long as they also have CKD.
  - CKD is defined by:
    - ICD10 codes (i.e., N18.x; E08.22; E09.22; E10.22; E11.22; E13.22 (exclude Z94.0; N18.6; Z99.2)) OR
    - Microalbumin/proteinuria level >30 mg/g for 2 time periods ≥ 3 months. Values taken within 12 months of enrollment, unless 2 values are unavailable, then review within 24 months of enrollment OR
    - 15 ≤ eGFR ≤ 60 ml/min for 2 time periods ≥ 3 months
  - Diabetes is defined by:
    - HbA1c ≥ 6.5 at least one time in the last year OR
    - ICD10 diagnosis codes (see Appendix A) OR
    - Having diabetes in the patient's medical record problem list

Exclusion Criteria
- Have diabetes, but no CKD.
- Are currently on dialysis (ICD 10 codes N18.6, Z99.2 and Z94.0)
- Have ESRD (eGFR<15 ml/min)
- Have a left ventricular assist device (LVAD)
- Have a terminal illness (specifically metastatic cancer, palliative care or hospice)
- Have patient-reported known pregnancy at time of enrollment
- Have had a liver, kidney, or allogeneic bone marrow transplant
- Too cognitively impaired to provide informed consent and/or complete the study protocol
- Institutionalized or too ill to participate (i.e. incarcerated, psychiatric or nursing home facility)
- Plan to move out of the area within 6 months of enrollment
- Not a current patient seeing a provider who cares for their hypertension (i.e., family medicine, internal medicine, nephrology, HIV provider, cardiology, hypertension specialists) at a participating site
- Previously participated in the GUARDD pilot study OR have previously undergone *APOL1* testing

**Primary Endpoint**	Change in Systolic Blood Pressure (SBP) from baseline to 3 months for *APOL1* positive participants.
**Secondary Endpoints**	The secondary endpoints for the main study include:<br>● Change in urine microalbuminuria/proteinuria orders from baseline to 6 months (Y/N)<br>● Documented order of microalbuminuria/proteinuria tests by 6 months (Y/N)<br>● Change in documented diagnosis for stage 3 CKD and above from baseline to 6 months (Y/N)<br>● Documented diagnosis of CKD stage 3 and above by 6 months (Y/N)<br>● Change in documented diagnosis for any stage CKD from baseline to 6 months (Y/N)<br>● Documented diagnosis of all stages of CKD by 6 months (Y/N)<br><br>The major endpoint for the PGx substudy is the change in systolic blood pressure from baseline to the 3 month study visit.
**Statistical Analyses**	<u>Main Study Analyses</u>:<br>● To determine the effect of participant and provider knowledge of a positive *APOL1* status on SBP, we will compare the change in SBP from baseline to 3 months of the Intervention – *APOL1* positive group to the change in SBP from baseline to 3 months of the Control - *APOL1* positive group using a two sided t-test, as appropriate, with an overall two-sided type I error of 0.05.<br>● The effect of knowledge of a positive *APOL1* status on all secondary endpoints will be compared between Intervention - *APOL1* positives to Control - *APOL1* positives with the proportion difference test.<br>● Additional analyses will include analysis of time trends in SBP, subset analyses, and exploratory analyses of cost effectiveness, mediators, moderators, psychobehavioral impact of results disclosure on participants, and effects of knowledge of *APOL1* status on provider treatment recommendations.<br><br><u>Substudy Analyses</u>:<br>● Major primary endpoint analyses conducted for the *APOL1* main study will be repeated for the PGx substudy focusing on differences in outcomes between *APOL1* negative individuals with immediate PGx ROR (PGx Intervention) and *APOL1* negative individuals with delayed PGx ROR (PGx Control).

2. INTRODUCTION

2.1 Background, Significance, and Rationale

Chronic kidney disease (CKD) is associated with hypertension and diabetes and affects 26 million US adults.[1] African Americans (AA's) have higher risk of end stage renal disease (ESRD) necessitating maintenance dialysis or transplant than Whites.[2] This disparity widens as kidney function declines. Additionally, AAs have the highest prevalence of hypertension (41% AA, 28% White) and the lowest rate of blood pressure (BP) control (43% AA versus 53% White).[3] While improved BP control decreases CKD progression and reduces morbidity and mortality, BP control (defined as < 140/90 mm Hg[4-7]) is only achieved in 50% of participants.[8]

One in 7 AAs carry the Apolipoprotein L1 (*APOL1)* gene risk allele, but it is nearly absent in other populations. High-risk variants in the *APOL1* explain approximately 70% of the excess prevalence of CKD in AAs, conferring a 5 times higher risk for hypertensive CKD and a 10 times higher risk for ESRD.[9] This risk is conferred by two variants (G1 and/or G2) in the last exon of *APOL1* with the high-risk genotype being G1/G1; G2/G2 or G1/G2.[10] *APOL1* does not significantly increase CKD risk in hypertensive people with diabetes and normal renal function, but in hypertensive people with prevalent CKD, *APOL1* exacerbates worsening of kidney function and progression to ESRD regardless of diabetes diagnosis.[11] Among adults with CKD, specific medications (angiotensin-converting enzyme (ACE) inhibitors or angiotensin receptor blockers (ARBs)) are recommended for participants of all ancestries, but 50% of participants do not receive these medications. Compounding this issue is the fact that providers do not include CKD on the problem list of 40% of participants with lab evidence of CKD,[12] leading to widespread participant unawareness of CKD. Urine protein tests, essential for early diagnosis and prognosis of CKD, are missing in up to 30% of participants with CKD.[13]

In Genetic Testing to Understand Renal Disease Disparities (GUARDD), investigators randomized participants to immediate (Intervention) versus delayed (Control) *APOL1* testing. Results were returned to participants through culturally appropriate clinical coordinators trained by genetic counselors, and to providers through low-intensity clinical decision support (CDS) (including best practice alerts (BPAs) with the test results and links to more provider information, participant education, and a generic message about the importance of blood pressure control). The study included a narrower phenotype of participants, excluding those with CKD or diabetes. GUARDD showed a statistically significant reduction in SBP at 3 months when comparing: 1) *APOL1* positives and negatives in the Intervention group (-6±18 mm Hg vs -3±18 mm Hg, p=0.008 cross-sectional at 3 months), and 2) Intervention *APOL1* positives versus overall Controls (-6±18 mmHg vs -3±18 mmHg, p=0.04 cross-sectional at 3 months). GUARDD also showed a trend towards a higher proportion of participants receiving renal disease testing at 12 months, as captured through electronic health records, when comparing: 1) *APOL1* positives and negatives in the Intervention group (29% vs 24%, p=0.10 cross-sectional at 12 months), and 2) Intervention *APOL1* positives versus overall Controls (29% vs 20%, p=0.01 cross-sectional 12 months). Participants self-reported making lifestyle changes and taking more blood pressure medicines in response to positive versus negative *APOL1* results. In addition, over 95% of participants were satisfied with the type, timing, and amount of information they received. Although participants were offered the option to speak with or meet with a genetic counselor, none chose to do so. Using stakeholder-engaged processes, there was an 11% rate of refusal, 93% 3-month retention rate, and 88% 12-month retention rate.

Several new developments highlight the importance of a broader pragmatic clinical trial (PCT). The GUARDD study was not powered to compare those with high risk genotypes (*APOL1* positives) in the Intervention group to *APOL1* positives in the Control group. New evidence since the study's inception also allows investigators to make CDS tools more robust, which may lead to greater action on the part of providers to improve hypertension management and renal disease screening. Finally, new data show that genetic differences affect the response to antihypertensive medication therapy, particularly in AAs. While pharmacogenetic (PGx) testing may help guide

initial or add-on antihypertensive therapy management for patients with hypertension, the impact of PGx testing on BP has not been studied in clinical trials among general or AA populations.

2.2 Aims and Objectives of the Study

Because AAs are not routinely screened for *APOL1*, it is unknown whether participant or provider knowledge of genetic risk impacts participant care or outcomes. Accordingly, the main objective of GUARDD-US is to determine the effect of participant and provider knowledge of a positive *APOL1* status on BP, urine testing for CKD, and documented diagnosis of CKD.

The primary aim is to determine the effect of participant and provider knowledge of a positive *APOL1* status and accompanying guideline based clinical decision support (CDS) on blood pressure management on change in systolic blood pressure (SBP) from baseline to 3 months after randomization among the APOL1 positive participants. Secondary aims are to:

1. Determine the effect of participant and provider knowledge of a positive *APOL1* status on the probability of documented CKD diagnosis.
2. Determine the effect of participant and provider knowledge of a positive *APOL1* status on the probability of receiving a urine microalbumin/creatinine testing and ACE-I/ARB prescription based on results of the urine microalbumin level.
3. Explore cost effectiveness, mediators, moderators, psychobehavioral impact of results disclosure on participants, and effects of participant and provider knowledge of *APOL1* status on provider treatment recommendations.

In addition, GUARDD-US will include a substudy to determine the effect of knowledge of genetic test results that predict efficacy of various antihypertensive medications on change in SBP from baseline to 3 months in *APOL1* negative individuals.

3. ENDPOINTS

3.1 Primary Endpoint

The primary endpoint is the change in systolic blood pressure from baseline to the 3 month study visit in *APOL1* positive participants.

3.2 Secondary Endpoints

Secondary endpoints include:

1. Change in urine microalbuminuria/proteinuria orders from baseline to 6 months (Y/N)
2. Documented order of microalbuminuria/proteinuria tests by 6 months (Y/N)
3. Change in documented diagnosis for stage 3 CKD and above from baseline to 6 months (Y/N)
4. Documented diagnosis of CKD stage 3 and above by 6 months (Y/N)
5. Change in documented diagnosis for any stage CKD from baseline to 6 months (Y/N)
6. Documented diagnosis of all stages of CKD by 6 months (Y/N)

Please see Section 10.2 for the derivations of these endpoints.

3.3 Pharmacogenetic (PGx) Substudy Endpoints

The major endpoint for the PGx substudy is the change in systolic blood pressure from baseline to the 3 month study visit.

Additional exploratory endpoints and analyses for the PGx substudy will be described in a separate Statistical Analysis Plan (SAP).

4. STUDY DESIGN

4.1 Study Arms & Design

This is a prospective, multicenter, unblinded, two arm, randomized pragmatic clinical trial (PCT) (**Figure 1**). Participants will be recruited from several recruitment sites (hereafter referred to as site) and randomized in a 1:1 ratio to immediate *APOL1* gene testing and return of results (ROR) to participant and provider (Intervention arm) versus delayed *APOL1* gene testing and ROR to participant and provider (Control arm). The main study will compare outcomes in a subset of the randomized population: *APOL1* positive participants in the Intervention arm (i.e., early knowledge of *APOL1* status) versus *APOL1* positive participants in the Control arm (i.e., delayed knowledge of *APOL1* status) (red outline in **Figure 1**). Participants that are *APOL1* negative in the Intervention and Control groups will not be included in the main study analyses.

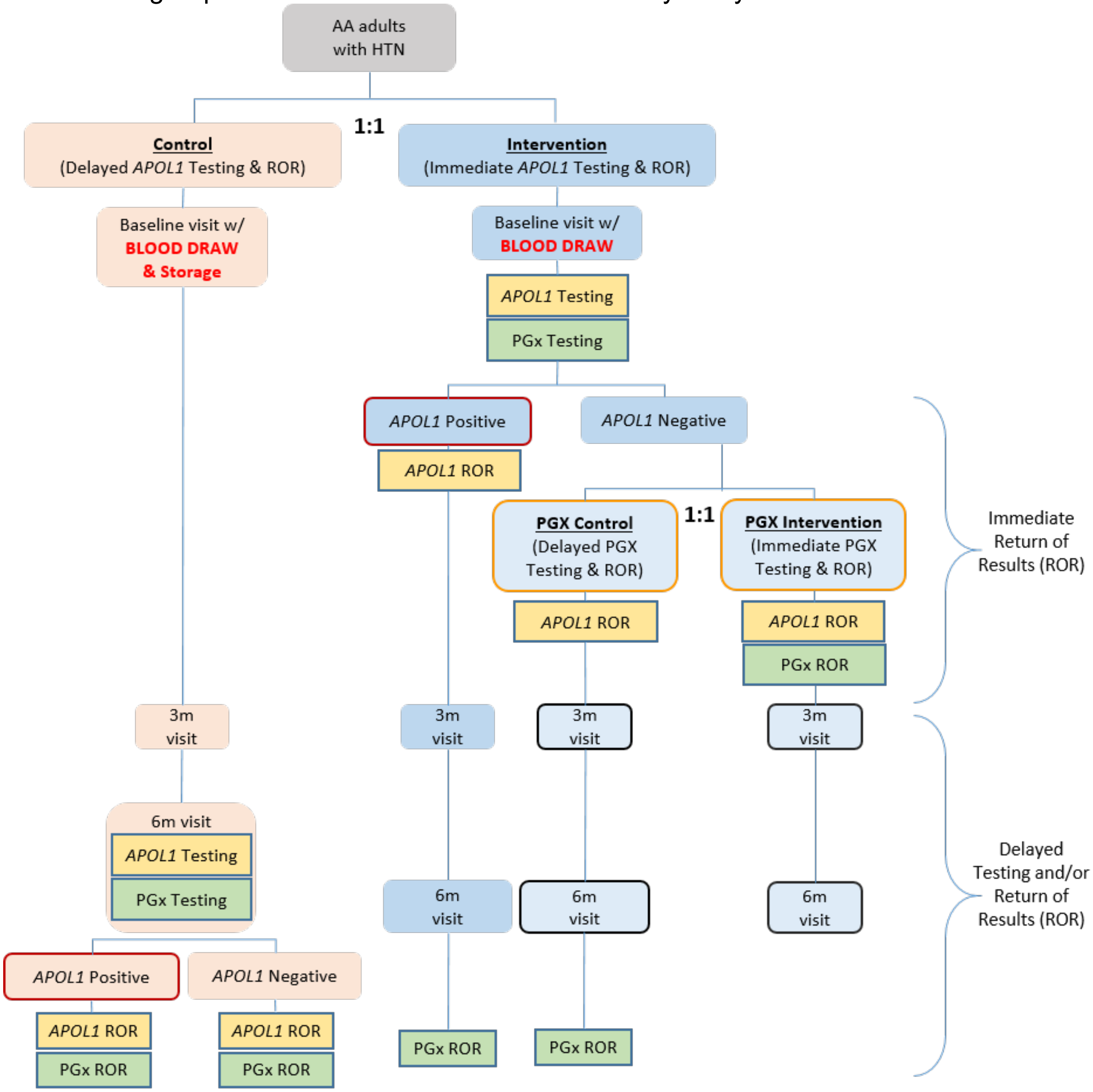

Figure 1. GUARDD-US study design for sites participating in PGx.

The primary outcome of the main study is systolic blood pressure (SBP) at 3 months in Control-*APOL1* positive and Intervention-*APOL1* positive participants (red outline). The substudy outcome is SBP at 3 months in Intervention-*APOL1*

Negative PGX Control and PGx Intervention participants (orange outline). AA = African Americans. *APOL1* = Apolipoprotein L1. HTN = hypertension. PGx = Pharmacogenetics. ROR = Return of Results.

GUARDD-US will also include a substudy that randomizes participants in the Intervention arm who test negative for *APOL1* to PGx Intervention (i.e., immediate PGx ROR) and PGx Control (i.e., delayed PGx ROR) in a 1:1 ratio. Only participants from the PGx substudy participating sites are included in this substudy. This substudy will compare outcomes between participants in the PGx Control group and the PGx Intervention group (orange outline in **Figure 1**).

The study design for sites that are not participating in the PGx substudy is summarized in **Figure 2.**

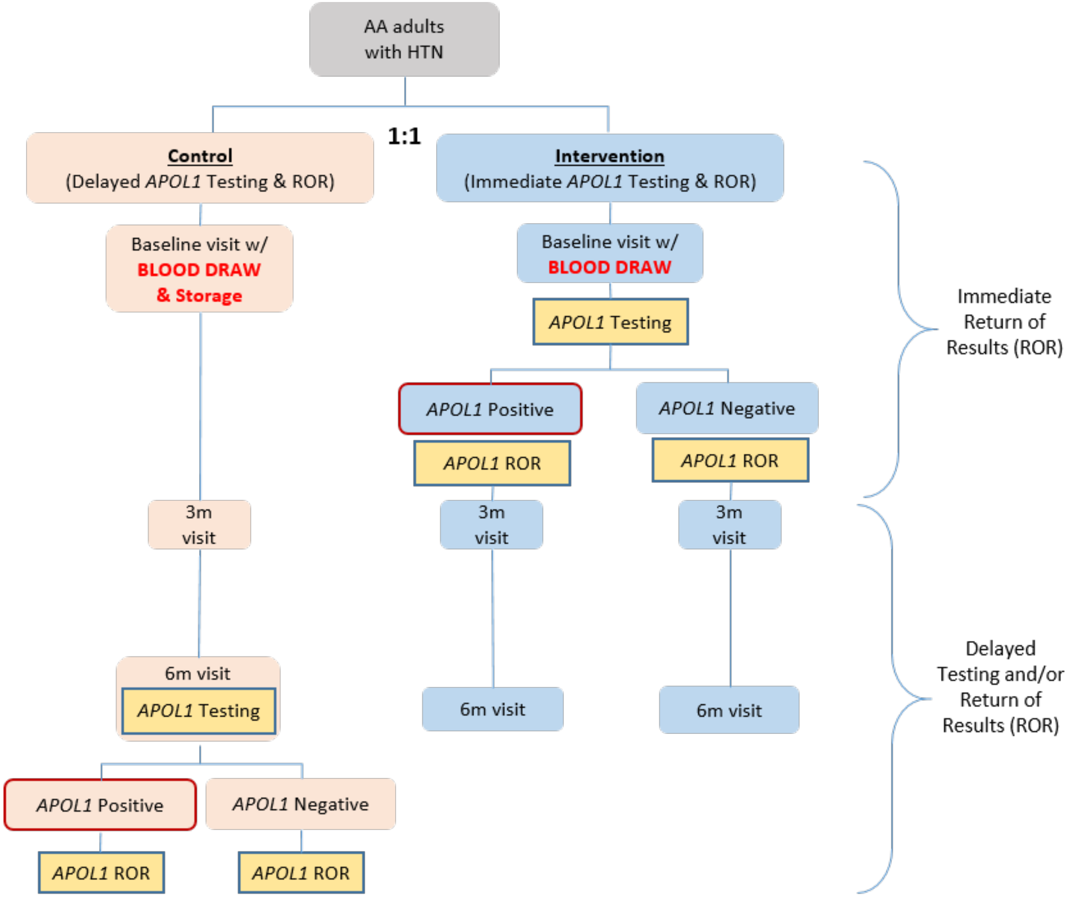

Figure 2. GUARDD-US study design for sites not participating in PGx.
The primary outcome of the main study is systolic blood pressure (SBP) at 3 months in Control-*APOL1* positive and Intervention-*APOL1* positive participants (red outline). AA = African Americans. *APOL1* = Apolipoprotein L1. HTN = hypertension. ROR = Return of Results.

4.2 Randomization

Eligible participants will be randomized in a 1:1 allocation to Intervention (i.e., immediate *APOL1* gene testing and ROR to participant and provider) and Control arms (delayed *APOL1* gene testing and ROR to participant and provider). Randomization will be stratified by clinical site with a random block size within site.

Participants that are randomized to receive immediate *APOL1* ROR will simultaneously be randomized to the PGx substudy intervention (i.e., immediate PGx gene testing and ROR to participant and provider) or the PGx control (delayed PGx gene testing and ROR to participant and provider) using a 1:1 allocation ratio. Participants from PGx substudy participating sites that test negative for *APOL1* will proceed into the PGx substudy under their assigned PGx treatment arms, while participants from non-participating sites of the substudy and participants that test positive for *APOL1* will be excluded from the substudy and receive delayed PGx gene testing and ROR.

4.3 Blinding

GUARDD-US randomization assignments will not be blinded to any participants, providers or study personnel because: 1) it is impossible to mask participants from the intervention testing and ROR, 2) research staff often receive questions from participants about the interpretation and risk associated with the participant's genetic results during the administration of surveys, and 3) resource limitations make hiring additional blinded staff to measure blood pressure impractical. To minimize bias in the measurement of the primary outcome, randomization assignments will only be revealed after baseline survey responses and blood pressure readings have been collected, digital blood pressure devices (such as Professional Intellisense Blood Pressure Monitor OMRON-907[14]) will be used to measure blood pressure, and blood pressure will be measured as the mean of the second and third blood pressure readings for each participant at each visit. Manual blood pressures may be collected if necessary.

4.4 Sample Size

Main Study: GUARDD-US will randomize approximately 6750 individuals with approximately 3375 individuals in the Control Arm and 3375 in the Intervention arm. Assuming a 14% prevalence of *APOL1* positive individuals, a sample size of 6700 for the randomized population would result in 938 *APOL1* positive individuals with at least 469 in each arm for the main study population. This sample size will detect a 3.5 mm Hg difference in the change in SBP between baseline and 3 months between Intervention - *APOL1* positive and Control - *APOL1* positive groups with 80% power. Since APOL1 result data and SBP change data could be missing for various reasons, an approximately 50 individuals will be randomized in addition to the 6700 sample size. Therefore, the total randomization target is approximately 6750. Please see Section 9.1 for assumptions, clinical significance, and detailed power and sample size calculations.

PGx Substudy***:*** Of the approximately 6750 participants in the GUARDD-US study, approximately 4400 will also be at a site participating in the PGx substudy. A sample size of 4400 with a 14% prevalence of *APOL1* positive individuals would result in 1892 participants for the PGx substudy (Intervention – *APOL1* negative participants) with 946 participants in the PGx Control and PGx Intervention arms. This sample size will detect a 3.1 mm Hg difference in the change in SBP from baseline to 3 months between the PGx Intervention group and PGx Control group with approximately 90% power. Please see Section 9.1 Sample Size Determination for assumptions, clinical significance, and detailed power and sample size calculations.

5. STUDY POPULATION

5.1 Inclusion Criteria

- Self-reported African ancestry
- English Speaking
- Age 18-70 years
- Have diagnosis of hypertension
  - Diagnosis of hypertension is defined by either:
    - ICD10 diagnosis codes (i.e., I10; I11.x; I12.x; I13.x; I16.x) OR
    - On active antihypertensive therapy for indication of hypertension OR
    - Having systolic blood pressure of 140 mm Hg or greater in at least 2 of the last 3 consecutive recorded values in the EHR OR
    - Having hypertension in the patient's medical record problem list
- Have been seen at ≥1 time in past year at a participating primary care site
- Either: 1) do not have diabetes and do not have CKD, or 2) have CKD; Participants with diabetes may be included as long as they also have CKD.
  - CKD is defined by:
    - A) ICD10 codes (i.e., N18.x; E08.22; E09.22; E10.22; E11.22; E13.22 (exclude Z94.0; N18.6; Z99.2)) OR
    - B) Microalbumin/proteinuria level >30 mg/g for 2 time periods ≥ 3 months. Values taken within 12 months of enrollment, unless 2 values are unavailable, then review within 24 months of enrollment.

      1) If there is only 1 proteinuria in E.H.R. (or if there are multiple and they are all within 3 months), any single proteinuria/microalbuminuria >30 mg/g would qualify

      2) If there are two or more proteinuria measures in E.H.R. that are at least 3 months apart AND are >30 mg/g would qualify

         a. Even if there are multiple proteinuria measures in the E.H.R., and only two of them are >30 mg/g, but they are separated by at least 3 months would qualify

         b. If there is only 1 proteinuria measure >30 mg/g and there are 2 or more proteinuria measures separated by 3 months within the prior 12 months would not qualify

      3) If there are not 2 proteinuria measures in 12 months, review within 24 months of enrollment.

      OR
    - C) 15 ≤ eGFR ≤ 60 ml/min for 2 time periods ≥ 3 months

      GFRs are taken within 12 months of enrollment, unless 2 values are unavailable, then review within 24 months.

      1) If there is only 1 GFR in E.H.R. (or if there are multiple and are all within 3 months), any single GFR between 15-60 would qualify

      2) If there are two or more GFRs in E.H.R. that are at least 3 months apart AND are between 15-60 would qualify

         a. Even if there are multiple GFRs in the E.H.R., and only two of them are between 15-60, but they are separated by at least 3 months would qualify

         b. If there is only 1 GFR between 15-60 and there are 2 or more GFRs separated by 3 months within the prior 24 months would not qualify

      3) If there are not 2 GFRs in 12 months, review within 24 months.
  - Diabetes is defined by:
    - HbA1c ≥ 6.5 at least one time in the last year OR

- ICD10 diagnosis codes (see Table 10, Appendix A) OR
- Having diabetes in the patient's medical record problem list

5.2 Exclusion Criteria

- Have diabetes, but no CKD.
- Are currently on dialysis (ICD 10 codes N18.6, Z99.2 and Z94.0)
- Have ESRD (eGFR<15 ml/min)
- Have a left ventricular assist device (LVAD)
- Have a terminal illness (specifically metastatic cancer, palliative care or hospice)
- Have patient-reported known pregnancy at time of enrollment
- Have had a liver, kidney, or allogeneic bone marrow transplant
- Too cognitively impaired to provide informed consent and/or complete the study protocol
- Institutionalized or too ill to participate (i.e. incarcerated, psychiatric or nursing home facility)
- Plan to move out of the area within 6 months of enrollment
- Not a current patient seeing a provider who cares for their hypertension (i.e., family medicine, internal medicine, nephrology, HIV provider, cardiology, hypertension specialists) at a participating site
- Previously participated in the GUARDD pilot study OR have previously undergone *APOL1* testing

6. RECRUITMENT AND ENROLLMENT PROCEDURES

6.1 Provider Recruitment, Assent, and Consent

Providers (including physicians, nurse practitioners, and provider assistants) that care for participants with hypertension (including, for example, general internists, family doctors/nurses, and nephrologists): 1) should be notified that their patients will be contacted to participate in the study, per site-specific institutional guidelines, and 2) may be asked to participate in a baseline provider survey. Provider assent may be obtained as required per institutional guidelines. Site/clinical champions and GUARDD-US staff may present the study to providers at scheduled meetings and also in one-on-one encounters or email at various recruitment sites. Provider survey responses should be recorded in the study database. Study staff may send reminders to providers to increase survey completion rates.

6.2 Participant Recruitment Strategies

Electronic Health Record (EHR) Data Queries for Eligible Participants
Sites may query EHRs to identify potentially eligible participants using the inclusion/exclusion criteria (see the manual of operations (MOP) for further details).

Participant Recruitment
Participants may be approached for the study in various ways following local policies and procedures including, for example, recruitment letters mailed directly to patients, clinic intercepts, study flyers, clinic referrals, or directly in clinic during a regularly scheduled clinic appointment. Participant recruitment letters may be created according to site-specific institutional policies, including, for example, instructions for how to opt out of study participation. Research Coordinators can also approach potentially eligible patients at clinic appointments. Research Coordinators may also provide clinical staff with study referral cards with eligibility criteria and study contact information for patient referrals. Clinics may have posters, flyers, and advertisements on TV monitors in waiting rooms with a study description and Research Coordinator contact information. If a patient expresses interest in participating, Research Coordinators may use a recruitment script (provided in the MOP) to do an initial screen for eligibility and then schedule the baseline visit in the future or that same day. Study staff may also ask current participants if they know anyone who would qualify for the study and provide them with study-related contact information to share with others.

Stakeholder Group and Additional Recruitment Materials
Diverse groups of stakeholders consisting of patient advocates, clinicians and community leaders will be engaged throughout the study process. These will include the Sinai Genomics Board, the Implementing Genomics in Practice Pragmatic Trials Network (IGNITE PTN) Patient, Provider, and Payer Advisory Board, and where available, stakeholders from sites. They may meet regularly to review all patient facing materials, including consent forms and patient recruitment and educational materials (recruitment letters, scripts, flyers, posters, etc.), for clarity and content. They may also provide input on recruitment strategies, addressing study design and challenges.

6.3 Screening Procedures

Research Coordinators may use a recruitment script (provided in the MOP) during recruitment phone calls or clinic intercepts (described above) to inform the potential participant about the study and screen participants for eligibility using the study inclusion/exclusion criteria. If the participant is eligible and interested, a baseline visit should be scheduled in the future or that same day. Screening may also be performed in a location agreed upon between a potential participant and the research team.

6.4 Participant Consent Process

If a participant is interested and eligible, Research Coordinators will review the consent document prior to enrolling the participant. Prospective research participants will have the opportunity to ask questions before providing written consent. Research Coordinators will provide the participant with a signed copy or version of the consent document. An original signed consent form is maintained by the Research Coordinator to be filed per site policies. If the individual chooses not to sign the consent form, the Research Coordinator will inform him/her they are unable to participate in the study. The consenting process can also be remote, via phone or electronically if approved by the reviewing IRB. A participant can complete the baseline visit and be randomized into the study within 30 days of providing written consent. If randomization does not occur within 30 days then this participant would be considered a screen failure.

Research participants will also be informed that they may be given the option to participate in other studies and provide additional biological specimens or data for those studies, which will require separate informed consent. They will also be provided the option to have their data collected as part of this study stored and shared for future related research, according to site-specific institutional policies.

6.5 Participant Discontinuation/Withdrawal from the Study

Participants may stop participating or withdraw from the study at any point in time. All information and data collected from the participant up to that point can be used in the study. Withdrawal of consent to participate in the research study can be verbal or in writing, however, revocation of HIPAA authorization must be in writing. Research Coordinators should attempt to obtain a reason for withdrawal from the participant and record it in the study database.

6.6 Lost to Follow-Up

Losses to follow-up may be minimized and retention maximized through various mechanisms, including offering study visits during evening and weekend hours, collecting information for and contacting family members when participants cannot be reached, approaching research participants at clinic appointments, and completing surveys over-the-phone (although research participant should still attend study visits for blood pressure measurement). Certified letters may also be sent to those not reached by phone. Research Coordinators should confirm the best contact information for the participant at each study visit. Research Coordinators may also obtain permission to contact patients via text message or email and may send additional correspondence during the study. Participants may be assigned to a specific Research Coordinator at the site in order to maintain continuity and build rapport.

6.7 Risk

This research presents minimal risks to participants. Possible risks are described below.

<u>Blood Pressure</u>
Participants may feel some arm pressure when the blood pressure cuff is briefly inflated.

<u>Blood Draw</u>
The risks of a blood draw include pain, bruising, and the slight possibility of infection at the location of needle insertion. Some participants may feel dizzy or may faint during or after a blood draw.

<u>Learning test results</u>
Results of blood, saliva, or buccal cell tests may show that a participant is at an increased risk of kidney disease. This knowledge may cause anxiety or psychological distress. Study staff should be trained to recognize anxiety and psychological stress and to attempt to relieve patient discomfort via discussion. All test results will be

discussed with the participant at the time of ROR. If systolic blood pressure exceeds 190 mm Hg or diastolic exceeds 110 mm Hg during any study visit, the participant will be strongly advised to seek urgent and appropriate evaluation and care from a healthcare provider. The Research Coordinator may facilitate this by assisting the participant in contacting their primary care provider, urgent care, or clinic staff on site including the Principal Investigator (if study visit is taking place in a clinical setting).

Delay in Learning Test Results
There is a risk that some patients in the Control group will have a 6 month delay in learning positive genetic test results relative to those in the Intervention group. Earlier knowledge of positive *APOL1* status could improve patient care and health outcomes. However, because *APOL1* testing is not currently considered standard care, the risks are low compared to patients receiving standard care.

Genetic Discrimination
While the Genetic Information Nondiscrimination Act (GINA), generally makes it illegal for health insurance companies, group health plans, and most employers to discriminate against participants based on their genetic information, federal law does not protect patients against genetic discrimination by companies that sell life insurance, disability insurance, or long-term care insurance.

6.8 Benefit

Research participants may not directly benefit from taking part in this research. However, some participants and their doctors may become aware of possible kidney disease earlier or which blood pressure medications are most efficacious. Providers may be able to make better decisions about the kind of treatment patients receive, thus providing some indirect benefit to participant health.

6.9 Costs to the Participants

Taking part in this research study may lead to minor added costs including, for example, transportation to attend study visits.

6.10 Compensation to Participants

Research participants will receive compensation throughout the course of the study for their time and effort. They will receive compensation for completion of each study visit – baseline, 3-month follow up, and 6-month follow up. Compensation may also be provided, on a pro-rated basis, for survey completion over the phone. Participants may be reimbursed for travel and/or parking expenses per institutional specific policies.

7. STUDY PROCEDURES

This section provides an overview of the study procedures to be followed in GUARDD-US. Further details will be provided in a MOP. Sites will receive training on the protocol and MOP before beginning enrollment.

7.1 Interventions/Treatments

The intervention for GUARDD-US is intended to reflect the practices and procedures that are likely to be implemented if *APOL1* testing were to be integrated into standard clinical practice. It includes the following components for participants and providers:

Participants

- An educational booklet given to all participants at the baseline visit that includes information about high blood pressure, kidney disease, and *APOL1* gene testing, and language recommending that participants speak with their provider if their BP is >130/80 mm Hg.
- The results of participant blood pressure readings at each study visit including
  - the mean of the second and third blood pressure readings
  - an indication of whether the mean value is normal (<130/80 mm Hg), up to your provider (between 131/81 mm Hg and 140/90 mm Hg) or elevated (>140/90 mm Hg)
  - a recommendation to speak with a provider if the participant's BP is above normal
- The verbal return of *APOL1* genetic testing results (i.e., *APOL1* positive or negative) and a verbal explanation of the meaning of the genetic testing results for risk of CKD and hypertension to participants following the schedule of activities and procedures for the participant's *APOL1* randomization assignment (see Section 7.10).
- A written explanation of the clinical implications of the patient's *APOL1* genetic testing results given to participants at the time of ROR.
- The same educational booklet will be given to participants as a reminder at the time of ROR.
- The option for patients to speak with a genetic counselor about their results.

Providers

- The electronic or written return of *APOL1* genetic testing results (i.e., *APOL1* positive or negative) to patient providers via standard site-specific laboratory return of results methods at approximately the same time or after participants have received their *APOL1* genetic testing results.
- Where possible, the electronic return of positive *APOL1* gene testing results to providers via a CDS alert that includes the participant's positive *APOL1* status, the risks associated with the participant's *APOL1* status, and treatment recommendations (including treatment for elevated BPs, ordering urine proteins tests, and ordering ACEs/ARBs) at approximately the same time or after participants have received their *APOL1* genetic testing results (see Section 7.8 for details).
- Educational materials explaining *APOL1* gene testing and clinical implications may be given to providers at the same time or after they have received patient *APOL1* genetic testing results.

During the baseline visit, all participants should receive an educational booklet with information about high blood pressure, kidney disease, and *APOL1* gene testing. This document will be identical for all participants. Participants will be notified of their *APOL1* randomization assignment after completing the baseline visit. At the end of each study visit (i.e., baseline, 3 month, 6 month), all participants will also receive the results of their blood pressure readings including the mean of the second and third blood pressure readings and an indication of whether this measurement is normal (<130/80 mm Hg), up to your provider (between 131/81 mm Hg and 140/90 mm Hg) or elevated (>140/90 mm Hg). Participants in the Control arm will receive their *APOL1* genetic testing results and an explanation of the meaning of their genetic testing results for risk of CKD and hypertension by phone and/or in-person after their 6 month study visit, while participants in the Intervention arm will receive their *APOL1* genetic testing results and an explanation of the meaning of their genetic testing results via phone and/or

in-person as soon as their results are available after the baseline visit. All participants will be given the option to speak to a genetic counselor as soon as results are returned. In addition to verbal ROR, participants will also receive lay explanations of their test results in writing and the educational booklet at the time of ROR in case the education booklet given at baseline was misplaced. These documents will be sent via mail and/or secure email to participants after the study team has attempted to verbally notify the participant of their test results. Written explanations of the test results and educational booklets will be identical for both Control and Intervention participants. In addition to study visits, participants may also have non-study related visits with their provider/s during the study. All participants may receive treatment recommendations or therapies for hypertension as part of these routine care visits.

Providers may be notified of participant results via standard site-specific laboratory return of results methods and, where possible and for positive *APOL1* results, a CDS alert within the electronic health system, as described in Section 7.8. Providers may also receive electronic links for accessing provider and participant educational materials through the CDS or other means. Providers of participants in the Intervention arm may receive notifications of the participants *APOL1* gene status during the study (via CDS alert or standard site-specific laboratory return of results methods). Providers of participants in the Control arm may be notified of participant *APOL1* gene status after the participant's 6 month study visit. Details of the ROR are presented in Section 7.6 and the schedule of activities is presented in Section 7.10. **Table 1** describes the *APOL1* variants and their predicted CKD risk.[9]

Table 1. *APOL1* Laboratory Report Language

Gene	Result	Predicted Risk Status
APOL1	*G2/*G2	Increased Risk of Chronic Kidney Disease Incidence and Progression
	*G1(GM) HET	Standard Risk of Chronic Kidney Disease Incidence and Progression

The intervention for the PGx substudy is also intended to reflect the practices and procedures that are likely to be implemented if PGx testing were to be integrated into standard clinical practice. It includes the following components for participants and providers:

<u>Participants</u>

- An educational booklet explaining PGx testing and clinical implications for hypertension medication management given to participants at the baseline visit.
- The return of PGx genetic testing results to participants following the schedule of activities and procedures for the participant's PGx randomization assignment.
- A written explanation of clinical implications of the patient's PGx genetic testing results including the genes tested and phenotypes associated with the efficacy of hypertension medications given to participants at the time of ROR.
- An educational booklet given to participants at the time of ROR.

<u>Providers</u>

- The return of PGx genetic testing results to patient providers via standard site-specific laboratory return of results methods at approximately the same time or after participants have received their PGx testing results.

- Where possible, a CDS provider alert for actionable phenotypes when a relevant medication is or may be ordered that indicates the participant's genetic results, the predicted phenotype (i.e., efficacy of various hypertension medications), and treatment recommendations at approximately the same time or after participants have received their PGx testing results.

Tables 2 and **3** describe the genes, variants, and phenotypes for the PGx substudy. The PGx sub-study will examine the efficacy of providing genetic information for two gene-drug pairs. The first gene-drug pair is *YEATS4* and thiazide diuretic efficacy, and focuses on first-line antihypertensive therapy in African Americans. The second gene-drug pair is *NAT2* and hydralazine. Hydralazine is not first line therapy, but is used in the treatment of resistant hypertension. GUARDD-US will include patients with both hypertension and resistant hypertension. Neither gene-drug pair is presently recommended by CPIC, but both have significant evidence supporting their relationship in populations that include individuals of African ancestry which is summarized in **Appendix B**.

Table 4 describes the PGx recommendations to be given for actionable genotypes. Each recommendation will be provided in the laboratory return of results and via a CDS alert, where available, that will be activated when a provider prescribes a medication that is not recommended based on the participant's genotype. Please see section 7.6 for more details.

Table 2. Genes and gene variants to be tested in the PGx substudy.

Gene	Variant
NAT2	rs1801279, rs1801280, rs1799930, rs1799931
YEATS4	rs7297610

Table 3. PGx genotypes, phenotypes, and predicted genotype frequencies.

Gene	Genotyping Result	Predicted Phenotype	Phenotype Frequency
NAT2	1 or more copy of *4	Reduced Hydralazine Efficacy	0.50
NAT2	2 copies of *5, *6, *7, *14[b]	Increased Hydralazine Efficacy	0.50
YEATS4	T/T or T/C	Reduced Thiazide Efficacy	0.51
YEATS4	C/C	Standard Thiazide Efficacy	0.49

Table 4. PGx recommendations[a]

Goal (Drug Class)	Gene	Genotype	Recommendation
First Line Therapy (Thiazide diuretic)	*YEATS4*	C/C	This individual is predicted to have standard thiazide efficacy. Consider use of a thiazide diuretic or calcium channel blocker as first line treatment of hypertension.
First Line Therapy (Thiazide diuretic)	*YEATS4*	TT or TC	This individual is predicted to have reduced thiazide efficacy. Consider use of an alternate first line therapy such as a calcium channel blocker for treatment of hypertension.
Resistant Hypertension (Hydralazine)	*NAT2*	*4/*X	This individual is predicted to have reduced hydralazine efficacy and may require a 50-100% higher starting dose or an alternate agent such as clonidine, spironolactone, or doxazosin for resistant hypertension.
Resistant Hypertension (Hydralazine)	*NAT2*	2 non-*4 alleles	This individual's NAT2 genetic test result is associated with increased hydralazine levels, which may lead to increased efficacy and/or adverse effects.

[a]Detailed references and rationale are summarized in Appendix B

7.2 Baseline Assessments

At baseline, which may occur in a location agreed upon between potential participants and the research team, Research Coordinators will verify that the participant has given consent. If participant has not given consent, Research Coordinators will obtain consent and information for multiple methods of patient contact (including mailing address, all phone numbers, and next of kin contact information). Research Coordinators will administer a survey (approximately 30 minutes long) to obtain information regarding the demographics, comorbidities, psychosocial factors, and knowledge, attitudes and beliefs towards genomics, kidney disease and hypertension. Survey responses will be recorded by the Research Coordinator in the study database and may be administered over the phone. A paper version of the survey can be used during a study visit, with survey data entered into the study database at a later time. Blood pressure will be measured according to the GUARDD-US Blood Pressure Protocol (details provided in the MOP) using a digital blood pressure monitor (such as Professional Intellisense Blood Pressure Monitor, OMRON-907[14]). Manual blood pressures may be collected if necessary. Pulse measurement will also be recorded for each participant and a blood, saliva, or buccal cell sample will be collected (as described in Section 7.4). After completion of the baseline visit, the Research Coordinator will randomize participants to immediate or delayed *APOL1* genetic testing and give patients: 1) an educational booklet with information about high blood pressure, kidney disease, and *APOL1* gene testing, 2) the results of participant blood pressure readings including the mean of the second and third blood pressure readings and an indication of whether this measurement is normal (<130/80 mm Hg), up to provider (between 131/81 mm Hg and 140/90 mm Hg) or elevated (>140/90 mm Hg), and 3) the participant's randomization assignment.

During the baseline visit, if the third (3rd) of three (3) consecutively measured blood pressure readings is greater than or equal to 190 mmHg for SBP or greater than or equal to 110 mmHg for DBP, study staff must inform the participant that they have very high blood pressure and strongly advise that they get urgent and appropriate evaluation and care from a healthcare provider. Study staff must also inform study PIs and follow the elevated blood pressure protocol outlined in the MOP.

Genetic sample collection and blood pressure measurement should occur on the same day and randomization must occur within 72 hours of blood pressure collection.

7.3 Follow-Up Assessments

Participants enrolled in the study will meet with Research Coordinators in a location agreed upon between the participant and the research team for a baseline, 3 month, and 6 month study visit. Follow-up visits will be completed within 8 weeks after the projected follow-up date. At each follow-up visit, Research Coordinators will administer the study surveys to participants and record their responses, measure and record the participant's blood pressure according to GUARDD-US Blood Pressure Protocol (provided in the MOP) and pulse measurement. Participants will be compensated for each study visit (see Section 6.10). Whenever possible, participants will meet with the same Research Coordinator for follow-up visits to maintain continuity. Follow-up assessments at each visit will include only participant-reported surveys, blood pressure and pulse measurements. Follow-up surveys may also be administered over the phone.

If a study site has been unable to reach a participant for a follow up visit, a blood pressure measurement may be obtained from the EHR and recorded in the study database if it meets the following criteria: 1) the blood pressure was measured within the specified visit window for the missed projected visit date and 2) the visit was either a clinic visit or a hospital discharge. Within the projected follow-up window, the blood pressure reading that was collected closest to the survey completion date will be used.

During any follow-up visit, if the third (3rd) of three (3) consecutively measured blood pressure readings is greater than or equal to 190 mmHg for SBP or greater than or equal to 110 mmHg, study staff must inform the participant that they have very high blood pressure and strongly advise that they get urgent and appropriate evaluation and care from a healthcare provider. Study staff must also inform study PIs and follow the elevated blood pressure protocol outlined in the MOP.

7.4 Specimen Collection

At the baseline visit, Research Coordinators will consent participants if not previously consented, administer the baseline survey, take 3 blood pressure readings, and, obtain a genetic sample via blood draw, saliva or buccal cell collection, whichever is most appropriate for the site and if possible, participant preferences.

<u>Blood Draw Protocol</u>
A phlebotomist or a coordinator trained in phlebotomy should collect 1 tube (approximately 1-2 teaspoons or 5 mLs) of blood via venipuncture from participants willing to give a blood sample. Research Coordinators should follow the MOP for instructions regarding the collection, storage, and delivery of the genetic samples to the designated laboratory.

<u>Saliva Protocol</u>
Alternatively, participants may provide a saliva sample in lieu of a blood sample. Research Coordinators should follow the MOP for instructions regarding the collection, storage, and delivery of the sample to the designated laboratory**.** Patients giving a saliva sample should not eat, drink, smoke or chew gum for 30 minutes before sample collection. Previous studies have confirmed that saliva samples are a viable alternative as a source of DNA for genotyping procedures[15].

<u>Buccal Cell Protocol</u>
Alternatively, participants may provide a buccal cell sample in lieu of a blood or saliva sample. Sample collection swabs/brushes should be labeled by recruiters, and collection, storage, and delivery to the designated laboratory

will follow the Buccal Cell Collection Protocol provided in the MOP. Participants providing a buccal cell sample should not eat, drink, smoke, or chew gum for 30 minutes before sample collection.

7.5 Specimen Transfer and Genetic Testing Procedures

All collected specimens should be clearly marked with the participant ID and all other required fields according to the instructions in the MOP, and delivered to a designated laboratory. Samples from Intervention arm participants will be processed by the laboratory as soon as possible using analytically validated *APOL1* and PGx gene testing procedures. For Control arm samples, designated laboratories will either extract DNA as soon as possible after sample receipt, and store the DNA for later analysis or store the sample and extract DNA after the Control participant has completed the 6 month follow-up assessment. Control Arm DNA samples will be processed as soon as possible after the 6 month visit using analytically validated procedures. Genes to be tested for the PGx substudy along with genotyping results and predicted phenotypes are described in Section 7.1. Details of sample storage and transport will be presented in the MOP. All laboratories in GUARDD-US are Clinical Laboratory Improvement Amendments (CLIA)-certified for high complexity.

7.6 Return of Genetic Results

The designated laboratories will transfer participant genetic testing results to the IGNITE PTN Coordinating Center (CC) as soon as possible and follow standard site-specific procedures for return of results to providers. Genetic results will be imported into the study database and the site EHR, where available. The study database will notify study personnel when a participant's lab results are ready to be returned to the participant.

Participants will receive *APOL1* and/or PGx results via phone and/or in-person. Research Coordinators should call participants at the appropriate time (see **Figure 1** and **Tables 5-7**), follow the ROR script (provided in the MOP), and end with a speak back in which participants will explain their results back in order to ensure comprehension. Research Coordinators should note the date, time, and completion status of all RORs in the study database. All participants will receive their genetic test results with lay explanations in writing and an educational booklet explaining their results and genetic risks via mail and/or secure email. Research Coordinators will offer all participants the option to speak by phone or in person with a study genetic counselor about their results. If a participant wants to speak with a genetic counselor, the Research Coordinator should contact the genetic counselor for their site on behalf of the patient, and the genetic counselor should reach out to the patient for counseling.

Providers may be notified of participant results via standard site-specific laboratory return of results methods and, where possible, a CDS within the electronic health system as described in Section 7.8. The genetic testing results should also be stored in the participant's medical records.

7.7 Data Collection from the Electronic Health Record

Data from the local EHR will be used to determine: 1) components of the secondary endpoints (i.e., presence of CKD diagnosis codes in the 24 months prior to baseline and up to 8 months after baseline, urine tests obtained in the 24 months prior to baseline and in the 6 months after baseline), 2) events of interest (see Section 8.2), and 3) variables needed for Other Planned Analyses including Cost Effectiveness Analysis (CEA) (see Section 9.6). Sites will extract the relevant EHR data using prespecified algorithms developed centrally by the IGNITE PTN CC or using site-developed algorithms. If using the CC-developed algorithms, sites will modify the algorithm codes based on their own customized EHR system. Sites will be asked to conduct EHR data downloads after at least 25% of the participants have completed their 6 month visit, and approximately 6-12 months after the last patient is randomized. EHR results will be sent to the IGNITE PTN CC via secure data transfer and formatted for data analyses. Details of the EHR download and transfer process will be provided separately to relevant site personnel.

7.8 Clinical Decision Support System

While participants will be notified of their *APOL1* and PGx test results via a phone call and/or in-person, providers may be notified of participant results via standard site-specific laboratory return of results methods and, where possible, a CDS within the electronic health system. For *APOL1* testing, sites should work with their institutional informational technology departments to set up provider CDS alerts for positive *APOL1* results and for actionable PGx phenotypes when a relevant medication is ordered. *APOL1* alerts should include the participant's *APOL1* status, the risks associated with the participant's *APOL1* status, and treatment recommendations (including treatment for elevated BPs, ordering urine proteins tests, and ordering ACEs/ARBs), and additional educational materials delivered via the site-specific CDS implementation method. For PGx testing, provider alerts should indicate the participant's genetic results, the predicted phenotype (i.e., efficacy of various hypertension medications), and treatment recommendations.

Reflecting the pragmatic nature of the trial, the many involved research sites, and variations in local CDS policies, it is anticipated that there will be differences in CDS implementation details, but all sites will ensure that both participants and providers are alerted to *APOL1* and PGx test results and associated significance. Details of the alerts will be provided separately to relevant site personnel.

7.9 Provider Surveys

Providers that agree to participate in the baseline survey should be given a written baseline survey to complete or instructions for completing the baseline survey online.

7.10 Schedule of Activities

Providers should be recruited and asked to complete the baseline survey as soon as possible after site activation. CDS alerts for providers should be activated as soon as lab results are entered into the electronic health record.

Tables 5, 6, and 7 describe the participant schedule of events for GUARDD-US.

Table 5. Schedule of participant activities up to randomization for sites.

Activities	Prior to baseline		Baseline visit
Recruit participants	X		X
Screen potential participants for interest/ eligibility	X		X
Contact participant to schedule baseline visit	X		X
Obtain consent	X	OR	X
Obtain BP & pulse & return BP results to participant			X
Distribute educational booklet/s to participant			X
Administer baseline participant survey			X
Perform sample collection			X
Randomize to *APOL1* treatment at end of baseline visit & notify patients of their randomization assignment			X

Table 6. Schedule of activities following participant baseline visit for sites participating in the PGx substudy.

Activities	CONTROL GROUP				INTERVENTION GROUP											
					APOL1 Positive				*APOL1* Negative PGx Control Group				*APOL1* Negative PGx Intervention Group			
	After Baseline & before 3M visit	3M visit (+8 wks)	6M visit (+8 wks)	After 6M visit	After Baseline & before 3M visit	3M visit (+8 wks)	6M visit (+8 wks)	After 6M visit	After Baseline & before 3M visit	3M visit (+8 wks)	6M visit (+8 wks)	After 6M visit	After Baseline & before 3M visit	3M visit (+8 wks)	6M visit (+8 wks)	After 6M visit
Participant surveys, BP & pulse measurements, & return of BP results to participant		X	X			X	X			X	X			X	X	
APOL1 testing conducted at laboratory				X	X				X				X			
Return *APOL1* test results to participant verbally and with written explanation				X	X				X				X			
Give educational booklet for *APOL1* results via mail or secure email				X	X				X				X			
PGx testing conducted at laboratory				X	X				X				X			
Return PGx Results verbally and with written explanation				X				X				X	X			
Give educational booklet for PGx results via mail or secure email				X				X				X	X			

Table 7. Schedule of activities following participant baseline visit for sites participating not in the PGx substudy.

Activities	CONTROL GROUP				INTERVENTION GROUP			
	After Baseline & before 3M visit	3M visit (+8 wks)	6M visit (+8 wks)	After 6M visit	After Baseline & before 3M visit	3M visit (+8 wks)	6M visit (+8 wks)	After 6M visit
Participant surveys, BP and pulse measurements, & return of BP results to participant		X	X			X	X	
APOL1 testing conducted at laboratory				X	X			
Return *APOL1* test results to participant verbally and with written explanation				X	X			
Give educational booklet for *APOL1* results via mail or secure email				X	X			

8. SAFETY ASSESSMENT AND MONITORING

GUARDD-US is an observational-type study that does not include a drug or device intervention. For this reason, no adverse events will be collected or recorded in the study database. Distress from return of results and events of interest will be monitored (as described in the next sections) and a Data and Safety Monitoring Board (DSMB) will provide trial oversight. Adverse events suspected to be related to study interventions should be reported to the reviewing IRB following local policies. Reportable events including unanticipated study related deaths will be collected in the study database per IRB reporting policies.

8.1 Distress from Return of Results

Distress from return of results should be monitored at patient visits. If the participant seems overly distressed by the outcomes of the genetic test results, the Research Coordinator should:

1. Offer the participant the opportunity to speak to the genetic counselor. If the genetic counselor is not immediately available, the Research Coordinator should help coordinate a phone call or meeting with the genetic counselor based on participant's preference.
2. Inform the Project Manager who should then:
    - Inform PIs
    - Document the event in the notes field of the participant's study database record
    - Follow-up with participant

8.2 Events of Interest

EHR data will be reviewed to monitor emergency department visits and hospitalizations. Sites will be asked to conduct two EHR data downloads, one after at least 25% of the participants have completed their 6 month visit, and another approximately 6-12 months after the last patient is randomized. EHR results will be sent to the IGNITE PTN CC via secure data transfer and formatted for data analyses. Details of the EHR download and transfer process will be provided separately to relevant site personnel.

8.3 Safety Monitoring/Data and Safety Monitoring Board

The DSMB, appointed by the NHGRI, will be responsible for providing recommendations regarding the conduct of the study and guidance to ensure the safety and well-being of participants. The DSMB will meet semi-annually with one conference call and one in-person/virtual meeting. A DSMB Charter will be developed detailing the procedures to be followed. A Data and Safety Monitoring Plan and a separate DSMB statistical analysis plan (SAP) will be developed in collaboration with the IGNITE PTN, IGNITE PTN CC, and NHGRI, and enacted by the DSMB.

8.4 Early Termination and Participant Discontinuation

Early termination considerations will generally apply only to emerging issues of major concern, or problems with trial conduct that suggest the trial could not be completed successfully with a reliable conclusion in a feasible time frame.

The site investigator, sponsor or institution may stop involvement of any participant in this research study at any time without their consent. This may be because the research study is being stopped, the instructions of the study team have not been followed, the investigator believes it is in the participant's best interest, or for any other reason. If specimens or data have been stored as part of the research study, they too may be destroyed without participant consent.

9. STATISTICAL ANALYSIS PLAN AND SAMPLE SIZE

9.1 Sample Size Determination

Main Study

In GUARDD, there was a 13% prevalence of *APOL1* positive individuals among hypertension participants and a standard deviation of 18.1 mm Hg for SBP. A separate unpublished pilot study from Indiana University showed a 20% prevalence of *APOL1* positive individuals among participants with CKD and an estimated CKD prevalence of 50%. Finally, another study showed a 26% prevalence of *APOL1* positive AAs with CKD[9]. As such, the initial sample size determination assumed a 17% prevalence of *APOL1* positive individuals. However, observation of the prevalence of the *APOL1* positive study participants during the course of the study led investigators and the DSMB to believe that this assumption was too high and the actual prevalence in the study population was between 14 and 15 percent. Assuming a conservative14% prevalence of *APOL1* positive individuals based on these studies, a sample size of approximately 6700 for the randomized population would result in approximately 938 *APOL1* positive individuals with at least 469 in each arm for the main study population examined here. Using a two tailed t-test and assuming an overall 5% level of significance, 10% lost-to-follow up, two interim analyses, and a standard deviation of 18.1 mm of Hg, a sample size of 6700 for the randomized population and approximately 938 for the *APOL1* positive individuals will detect a 3.5 mm Hg difference in the change in SBP from baseline to 3 months between Intervention - *APOL1* positive and Control - *APOL1* positive groups with at least 80% power. This sample size determination assumes 10% of the 938 APOL1 positive individuals could drop out of the study before 3 months and therefore have incomplete data on SBP change from baseline to 3 months. APOL1 positive individuals with incomplete data on SBP change from baseline to 3 months cannot be included in the primary endpoint analysis. Therefore, the final analysis on the primary endpoint needs at least 844 APOL1 positive individuals who have complete data for SBP change from baseline to 3 months to detect a 3.5 mm Hg difference between two treatment groups. Enrollment will continue until the 844 target can be ensured. Since APOL1 and SBP data could be missing for various reasons, approximately 50 additional participants will be randomized to help us to reach this 844 target. This makes the total randomization target to be approximately 6750. Enrollment may end prior to the 6750 target if the study team is certain that the necessary 844 *APOL1* positive individuals with complete SBP change data can be achieved.

PGx Substudy

Of the approximately 6750 participants in the GUARDD-US study, approximately 4400 will also be at a site participating in the PGx substudy. A sample size of 4400 with a 14% prevalence of *APOL1* positive individuals would result in 1892 participants for the PGx substudy (Intervention – *APOL1* negative participants) with 946 participants in the PGx Control and PGx Intervention arms. Using a two tailed t-test and assuming a 5% level of significance, 10% lost-to-follow up, and a standard deviation of 20 mm Hg (obtained from an unpublished study from the University of Florida), a sample size of 4400 for the randomized population and 1892 for the PGx substudy will detect a 3.1 mm Hg difference in the change in SBP from baseline to 3 months between the PGx Intervention group and PGx Control group with approximately 90% power.

9.2 General Statistical Methods

A detailed SAP will be developed and contained in a separate document. Prior to analysis, study population details including the number randomized, in each treatment arm, and lost to follow-up will be described. Baseline participant characteristics will be summarized as means, standard deviations, medians, and/or 25th, 75th percentiles for continuous variables, and as counts and percentages for categorical variables. Model assumptions will be examined prior to analysis and transformations implemented, if necessary, to more adequately meet the assumptions. Unless otherwise stated, hypotheses will be tested as two-sided with a significance level of 0.05.

9.3 Population for Analyses

Population for Main Study Objectives
Participants from the Randomized Population who test positive for *APOL1*.

Population for PGx Substudy Objective
Participants from the PGx substudy participating sites who are randomized to Intervention in the main study and test negative for *APOL1* are included in the PGx substudy population. Only participants who have non-missing PGx test results are included in the population for the primary and secondary analyses.

9.4 Analysis of the Primary Endpoint

To determine the effect of knowledge of a positive *APOL1* status on SBP, we will compare the change in SBP from baseline to 3 months of the Intervention – *APOL1* positive group to the change in SBP from baseline to 3 months of the Control - *APOL1* positive group using a two sided t-test with an overall two-sided type I error of 0.05. Participants that are *APOL1* negative in both the Intervention and Control groups will not be included. This primary analysis will be conducted according to the principle of intention-to-treat (ITT) with participants analyzed and endpoints attributed according to the treatment arm to which the participants were randomized, regardless of subsequent crossover or post-randomization medical care within the pre-specified subset of *APOL1* positive participants.

9.5 Analysis of the Secondary Endpoints

The effect of knowledge of a positive *APOL1* status on secondary endpoints [i.e., change in urine microalbuminuria/proteinuria orders from baseline to 6 months (Y/N), documented order of microalbuminuria/proteinuria tests by 6 months (Y/N), change in documented diagnosis for stage 3 CKD and above (Y/N) from baseline to 6 months, documented diagnosis of CKD stage 3 and above by 6 months (Y/N), change in documented diagnosis for any stage CKD from baseline to 6 months (Y/N), and documented diagnosis of all stages of CKD by 6 months (Y/N)] will be compared between Intervention - *APOL1* positives and Control - *APOL1* positives with the proportion difference test.

9.6 Other Planned Analyses

Trends in SBP. In addition to the primary endpoint analysis described above, we will compare the time trends in SBP between Intervention – *APOL1* positive individuals and Control – *APOL1* positive individuals using a repeated measures mixed model. Since participants are randomized to Intervention and Control groups and not to the *APOL1* positive subgroups within Intervention and Control groups, there could be important differences in baseline characteristics between the two groups. For this reason, we will conduct covariate-adjusted repeated measures mixed models that will account for differences in baseline characteristics that differ between the Intervention – *APOL1* positive group and the Control – *APOL1* positive group. Potential baseline covariates include baseline SBP, age, sex, and baseline CKD. We will select the appropriate covariance matrix (e.g.,

compound symmetry, autoregressive, unstructured, or other covariance structure) based on the data to account for repeated measures.

CKD subset. If the data provide evidence of an overall difference in the primary outcome (change in SBP from baseline to 3 months) between Intervention – *APOL1* positive group and the Control – *APOL1* positive groups, we will further examine whether the effect of knowledge of a positive *APOL1* status on SBP differs among those with and without CKD. This subgroup analyses will involve testing for interactions between treatment arm and CKD using linear regression. Effect estimates will be carefully (conservatively) interpreted in conjunction with the formal interaction test. If the interaction is significant, we will test whether the time trends in effect of early versus delayed knowledge of a positive *APOL1* status on SBP differs among Intervention – *APOL1* positive group and the Control – *APOL1* positive groups in those with CKD at baseline using a covariate-adjusted repeated measures mixed model. Potential covariates are the same as described above. We will select the appropriate covariance matrix (e.g., compound symmetry, autoregressive, unstructured, or other covariance structure) based on the data to account for repeated measures.

Controlled versus Uncontrolled Blood Pressure Subset. We will also examine whether the effect of knowledge of a positive *APOL1* status on SBP differs among those whose blood pressure is well-controlled at baseline versus those whose blood pressure is not well-controlled at baseline. This subgroup analyses will involve testing for interactions between treatment arm and whether or not SBP is 140 mm Hg or greater at baseline using linear regression. Effect estimates will be carefully (conservatively) interpreted in conjunction with the formal interaction test. As an additional subgroup analysis, we will similarly examine the effect of knowledge of a positive APOL1 status on SBP between those on treatment for hypertension versus those not on treatment in the subset of participants with SBP 140 mm Hg or greater.

Cost-Effectiveness (CE). Complete details of the cost-effectiveness analysis (CEA) will be presented in a separate SAP depending on the outcome of the main study. Briefly, two analyses will be conducted comparing economic outcomes in Intervention – *APOL1* positive and the Control – *APOL1* positive groups. In the first, *within-trial cost analysis*, 6-month total healthcare cost will be estimated per participant using estimates of intervention costs (obtained from Centers for Medicare and Medicaid Services (CMS) physician fee schedule[17]), medication costs (obtained from participant survey, EHR costs per cost center and/or total costs, VAFFS drug prices[18], National Average Drug Acquisition Cost (NADAC) drug prices[19], Medicare drug prices[20]), and downstream health care costs of inpatient, outpatient, and emergency department use (from participant survey, EHR costs per cost center and/or total costs, Medicare outpatient prospective payment system (PPS) for healthcare common procedure coding system (HCPCS)[21], cost-to-charge-ratio (CCR) for estimating inpatient costs from charges[22]). The analyses will be adjusted for baseline 3-month total costs and utility scores by means of regression. The second, *long-term CE analysis*, will use a microsimulation model with five health states (Well, Cardiovascular Disease (CVD), ESRD, CVD plus ESRD, and Death) for predicting CE outcomes over a 5-year, 10-year and lifetime horizon following U.S. recommendations[23]. The microsimulation model's predictions of CVD, ESRD and competing mortality rates will be informed by published epidemiological risk models and U.S. life tables. Specifically, we will use the Pooled Cohort Equations[24] for modeling CVD and epidemiological studies for linking CKD stage to long-term ESRD risk with the following risk factors as input parameters: participant demographics (age, gender, and race) obtained from participant surveys; 6-month systolic blood pressure measurements; 6-month smoking status and preventive medication use (BP med) obtained from participant surveys; and 6-month diabetes status, eGFR, urine albumin to creatinine ratio, and blood cholesterol parameters obtained from the EHR. The estimated event rates of CVD, ESRD and competing mortality will be modified using expected rate ratios of blood pressure lowering therapy estimated from difference in 6-month change of systolic blood pressure from baseline. Rate ratios will be based on published meta-analyses of trials on blood pressure lowering medication. The microsimulation model will be used to estimate cumulative costs and QALYs as a function of life expectancy and simulated CVD and ESRD events for each trial participant. Long-term utility and cost penalties

conditioned on increasing age, CVD, and ESRD will be based on the literature. Details of the procedures for obtaining EHR data and estimates from the EHR will be provided in the MOP and SAP.

PGx Substudy. Analyses for the PGx substudy will compare outcomes between *APOL1* negative individuals with immediate PGx ROR (PGx Intervention) and *APOL1* negative individuals with delayed PGx ROR (PGx Control). A two sided t-test with a two-sided type I error of 0.05 will be used to evaluate differences in the change in SBP from baseline to the 3 month study visit between PGx Intervention and PGx Control groups. To evaluate time trends in SBP, we will also conduct covariate-adjusted repeated measures mixed models that will account for differences in baseline characteristics that differ between the PGx Intervention group and the PGx Control group. Potential baseline covariates are as described above. We will select the appropriate covariance matrix (e.g., compound symmetry, autoregressive, unstructured, or other covariance structure) based on the data to account for repeated measures. Additional exploratory endpoints and analyses for the PGx substudy will be described in a separate SAP.

Details of these statistical analyses along with all other exploratory analyses (e.g., mediators, moderators and psychobehavioral impact of results disclosure on participant outcomes and provider treatment recommendations, and medication adherence) will be described in the study SAP.

Long-Term Exploratory Outcomes:
Research participants can consent for long-term follow up of kidney function. We will use appropriate procedures (including secure linkage, honest broker procedures, etc.) to access several databases, including patient electronic medical records, or restricted databases such as USRDS (United States Renal Data System) and CMS to track kidney function measurements, blood pressure, medication usage and health outcomes related to kidney disease up to 10 years from the date of enrollment, for patients who agree. We will then analyze these data as part of our exploratory outcomes and assess effect of interventions on long-term kidney outcomes.

Impact of Social Determinants of Health:
Data from participant surveys will be used to explore the impact of individual-level social determinants of health on primary, secondary, and exploratory study outcomes. Participant address will be used for linkage to publicly available data on community-level determinants of health (eg, Social CDC Vulnerability Index).

9.7 Interim Analyses

A single planned interim analysis was planned initially for this study. To account for repeated significance testing of the accumulating data, the group sequential method of Lan and DeMets will be used as a guide for interpreting this interim analysis. Monitoring boundaries for the primary endpoint will be based on a two-sided symmetric Lan-DeMets spending function using O'Brien-Fleming type boundaries with an overall two-sided significance level of $\alpha=0.05$. The O'Brien-Fleming approach requires large critical values early in the study but relaxes (i.e., decreases) the critical value as the trial progresses. The primary and interim analyses will compare SBP at 3 months among *APOL1* positive individuals in the Control arm to *APOL1* positive individuals in the Intervention arm. Because *APOL1* genotype (i.e., positive vs. negative) for participants in the Control arm is determined around the time of the 6 month study visit, only participants that have completed their 6 month study visit will be included in the interim analysis. To account for the delays in receiving genetic testing results, the interim analysis will be targeted to occur when approximately 25% of the participants have completed their 6 month study visit. Using the O'Brien-Fleming type boundary at 25% information, if the analysis calculates a p-value that is less than 0.00009, stopping the trial for efficacy or harm will be proposed. In order to maintain the overall significance level of 0.05, the final analysis of the primary endpoint will be considered significant for p-values less than 0.049975.

If the interim analysis does not propose stopping for efficacy (p-value <0.00009 and the intervention has the larger reduction in SBP at 3 months), then the conditional power to detect a significant result at the end of the trial will be estimated. The conditional power will be presented to the DSMB to facilitate discussion of whether the trial should be stopped for futility.

An additional interim analysis was performed per request by the DSMB in May, 2022. In order to maintain the overall significance level of 0.05 after this second interim analysis, the definition of significant p-value for the final analysis is recalculated by continuing to use the Lan-DeMets function with O'Brien-Fleming type boundaries appropriately for the approximate percent information at which the interim analysis occurred. Due to the occurrence of this second interim analysis, the final analysis of the primary endpoint will be considered significant if p-value is less than 0.0492.

9.8 Handling of Missing Data

For the primary and secondary statistical analyses described above, multiple imputation will be used for all missing values except those due to participant death. To ensure that the missing at random assumption for multiple imputation is valid, we will compare baseline SBP and other patient characteristics in those lost to follow up to participants retained on their randomization assignment. We will also do sensitivity analyses where we compare results obtained with multiple imputation to those obtained without imputation. Missing values due to death will not be imputed and will therefore not be included in the analyses.

For exploratory repeated measures analyses, missing data are easily handled in repeated measures mixed models, as long as the missing at random assumption is valid. However, the missing at random assumption cannot be tested. Accordingly, for exploratory analyses using repeated measures mixed models, we will also conduct a sensitivity analysis where missing values are imputed using multiple imputation. The results with and without multiple imputation will be compared. More complete details of the handling of missing values under different circumstances will be described in the SAP.

9.9 Multiplicity

With the primary and various secondary endpoints that have been outlined, there is a multiplicity of analyses to be performed, which leads to an increased probability that at least one of the comparisons could be "significant" by chance. Adjusting for the effects of the repeated significance testing for the multiplicity of secondary endpoints would require that very small significance levels be used for every comparison. Therefore, rather than adjusting for multiple comparisons, statistical results (e.g., p values) will only be presented for the primary endpoint analysis. We will be conservative in the interpretation of secondary analyses and report only point estimates and 95% confidence intervals. We have also pre-specified the primary and secondary outcome variables to avoid over-interpretation of strictly exploratory comparisons.

10. DATA MANAGEMENT

10.1 Data Entry and Record Keeping

GUARDD-US will have 4 main data sources: data collected from participants (survey, blood pressure and pulse) and providers (survey), genetic testing results from designated laboratories, data from the EHR, and publicly available data for CEA analyses. Data from patients and providers should be entered directly into study database by trained and qualified personnel at each clinical site. Site staff will receive training on the use of the study database. After staff are trained, they will receive a unique user identification and password to access data entry forms for their site. Access codes should not be shared and are non-transferable.

Laboratories will transfer genetic testing results to the IGNITE PTN CC and follow standard site-specific return of results procedures for providers. The genotype and result (i.e., positive versus negative) for each participant will then be automatically imported to the participant's study database record from laboratory produced output files. The accuracy of the automatic import of laboratory results will be verified by comparing the results recorded in the study database to an external record of the result for a subset of the study participants.

Sites will extract the relevant data from their EHR (see Section 7.7) and transfer those data to the IGNITE PTN CC following procedures that will be specified. Briefly, sites will be given a list of information to extract for their GUARDD-US participants, a common format that data should be converted to, and procedures for secure file transfer to the CC. Transfers of data will occur after at least 25% of the participants have completed their 6 month visit and 6-12 months after the last participant is randomized.

Data to be used for cost-effectiveness analyses (CEA) will be obtained via participant surveys administered at study visits, direct measurement at study visits (for blood pressure), via the EHR, and via publicly available data sources (CMS physician fee schedule[17], VAFFS drug prices[18], NADAC drug prices[19], Medicare drug prices[20], PPS for HCPCS[21], and CCR for estimating inpatient costs from charges[22]). Publicly available data sources can be downloaded freely at the end of the study from corresponding online sources and stored on secure servers at the CC. Any specialized data collection procedures will be presented in the MOP. Details on the data to be used for CEA are presented in Section 9.6.

10.2 Data Element Definitions

Baseline SBP will be defined as the mean of the 2nd and 3rd measurements taken according to the GUARDD-US Blood Pressure Protocol provided in the MOP. Similarly, follow-up SBP will be the average of the 2nd and 3rd SBP measures at the follow-up visit, or alternatively the SBP from the EHR entered into the study database by the site in accordance with the study manual of procedures (MOP).

Secondary Endpoints will be defined as follows:

1. Change in urine microalbuminuria/proteinuria orders from baseline to 6 months (Y/N)
    a. Order of urine microalbuminuria/proteinuria test at baseline is a binary variable (Y/N) where 'Y' is defined as the presence of urine proteinuria or urine microalbuminuria diagnostic code (i.e. 82043/84156 (Current Procedural Terminology (CPT) codes) OR the presence of urine microalbuminuria/proteinuria lab test order and/or result in the participant's EHR record at any point in the 12 months prior to baseline.
    b. Order of urine microalbuminuria/proteinuria test at 6 months is a binary variable (Y/N) where 'Y' is defined as the presence of urine proteinuria or urine microalbuminuria diagnostic code 82043/84156 (CPT codes) OR the presence of urine microalbuminuria/proteinuria lab test order and/or result in the participant's EHR record at any point from baseline to 6 months.

c. Change in urine microalbuminuria/proteinuria orders is a binary variable (Y/N) where ‘Y’ is defined as no order of urine microalbuminuria/proteinuria test at baseline and having an order of urine microalbuminuria/proteinuria test at 6 months [No for 1a, Yes for 1b = NY].

2. Documented order of microalbuminuria/proteinuria tests by 6 months is a binary variable (Y/N) where ‘Y’ is defined as the change in urine microalbuminuria/proteinuria orders (as described in 1c) OR (order of urine microalbuminuria/proteinuria test at baseline) [NY, YN or YY]

3. Change in documented diagnosis for stage 3 CKD and above from baseline to 6 months (Y/N)
    a. Documented diagnosis at baseline is a binary variable (Y/N) where ‘Y’ is defined as [the presence of a CKD ICD10 diagnosis codes of N18.3 – N18.5 in the 24 months prior to baseline].
    b. Documented diagnosis at 6 months is a binary variable (Y/N) where ‘Y’ is defined as [the presence of a CKD ICD10 diagnosis codes of N18.3 – N18.6 in the 6 months after baseline].
    c. Change in documented diagnosis for stage 3 CKD and above is a binary variable (Y/N) where ‘Y’ is defined as no documented diagnosis at baseline and documented diagnosis at 6 months [No for 3a, Yes for 3b = NY].

4. Documented diagnosis of stage 3 and above CKD by 6 months is a binary variable (Y/N) where ‘Y’ is defined as (change in documented diagnosis for stage 3 CKD (as described in 3c)) OR (documented diagnosis at baseline (as described in 3a)) [NY, YN or YY].

5. Change in documented diagnosis for any stage CKD from baseline to 6 months (Y/N)
    a. Documented diagnosis at baseline is a binary variable (Y/N) where ‘Y’ is defined as [the presence of a CKD ICD10 diagnosis code (i.e., N18.x; E08.22; E09.22; E10.22; E11.22; E13.22) in the 24 months prior to baseline].
    b. Documented diagnosis at 6 months is a binary variable (Y/N) where ‘Y’ is defined as [the presence of a CKD ICD10 diagnosis code (i.e., E18.x; E08.22; E09.22; E10.22; E11.22; E13.22) in the 6 months after baseline].
    c. Change in documented diagnosis for any stage CKD is a binary variable defined as no documented diagnosis at baseline and documented diagnosis at 6 months [No for 5a, Yes for 5b = NY].

6. Documented diagnosis of any stage CKD by 6 months is a binary variable (Y/N) where ‘Y’ is defined as (the change in documented diagnosis of any stage CKD (as described in 5c)) OR (documented diagnosis at baseline (as described in 5a)) [NY, YN or YY].

For CEA analyses, the following definitions for variables obtained from the EHR will be used:

1. 6-month CKD stage will be defined with the follow categories using the most recent GFR measurement obtained between baseline and 6 months:
    a. Stage 1 with normal or high GFR (GFR > 90 mL/min)
    b. Stage 2 Mild CKD (GFR = 60-89 mL/min)
    c. Stage 3A Moderate CKD (GFR = 45-59 mL/min)
    d. Stage 3B Moderate CKD (GFR = 30-44 mL/min)
    e. Stage 4 Severe CKD (GFR = 15-29 mL/min)
    f. Stage 5 End Stage CKD (GFR <15 mL/min)
2. 6-month blood cholesterol: The last observed blood cholesterol level observed prior to baseline. Missing values will be imputed.

10.3 Database Management and Quality Control of Data

The IGNITE PTN CC will develop and manage the GUARDD-US study database and perform internal database quality-control checks. The CC will conduct data audits throughout the course of the trial. These audits are intended to identify data errors, protocol deviations, failure of standardization, missing data, or inconsistencies. Any out-of-range values and missing or inconsistent key variables are flagged and addressed/answered at the site in real time during the data entry process.

All data will be transferred daily from the study database to SAS datasets and stored on the CC's secure servers. The CC will periodically perform additional data quality checks in SAS. Clinical sites may also be given regular feedback directly to discuss issues identified by QC assessments.

10.4 Study Close Out

The end of the trial is defined as the end of participant follow-up in the trial. Upon completion of the study, the IGNITE PTN CC and/or their designee will notify the site of closeout. The CC and/or their designee will ensure that the Investigator's regulatory files are up to date and complete, and that any outstanding issues from previous correspondences have been resolved. Other issues to be reviewed at the closeout include: discussing retention of study files, possibility of site audits, publication policy, and notifying the IRB of study closure.

11. ETHICAL AND HUMAN SUBJECTS CONSIDERATIONS

11.1 Institutional Review Board/Ethics Committee Review

This study will be initiated only after all required documentation has been reviewed and approved by the central IRB according to national and international regulations. The investigator will provide the IGNITE PTN CC or their designee with the study approval documentation before the study may begin. The same is applicable for the implementation of changes introduced by amendments. Where applicable, the investigator must also provide to the IGNITE PTN CC and/or their designee the following documentation:

1. A copy of IRB annual re-approval of the protocol per current Title 21 CFR 312.66 regulations and 1997 International Conference on Harmonization guidelines.
2. IRB approval of revisions to the informed consent documents. Administrative changes (such as a change in address or phone number) must be sent to IRBs/Ethics Committees but do not require their approval.
3. The investigator must submit periodic status reports to their Executive Committee as required, as well as notification of completion of the study and a final report where applicable.
4. The investigator will provide the IGNITE PTN CC or their designee with documentation of all approvals.

11.2 Use and Disclosure of Protected Health Information

For clinical trial sites located in the United States, an Authorization for the use and disclosure of PHI under the HIPAA Privacy Rule [45 CFR § 164.102 *et seq*] will be obtained from every trial participant prior to, or at the time of, enrollment. The investigator is responsible for obtaining participants' authorizations and signatures, and for explaining the elements of the HIPAA Authorization form. HIPAA Authorization may either be a separate form or included in the study ICF, dependent upon local requirements.

11.3 Confidentiality and Privacy

Participant confidentiality will be maintained throughout the clinical study in a way that ensures the information can always be tracked back to the source data. For this purpose, a unique participant identification code (ID number) will be used that allows identification of all data reported for each participant. Participant information collected in this study and all records will be kept confidential.

Clinical data will be entered into an internet data entry system provided by the CC. The data system includes password protection and internal quality checks, such as automatic range checks to identify data that appear inconsistent, incomplete, or inaccurate. All data collection and storage devices will be password protected with a strong password and all sensitive research information on portable devices will be encrypted. Access to identifiable data will be limited to the study team. If it is necessary to use portable devices for initial collection of identifiers, the data files will be encrypted and the identifiers moved to a secure system as soon as possible. The portable device(s) will be locked in a secure location when it is not in use.

11.4 Publication and Data Sharing Policies

The IGNITE PTN will have a Publications and Presentations Committee (PPC) with the primary responsibility for coordinating, monitoring, and reviewing all publications and presentations resulting from IGNITE PTN studies. In addition, the PPC will oversee the review, approval, and supervision of the secondary analyses and ancillary studies that are conducted within the Network. The goal of the PPC is to facilitate dissemination of the maximum amount of information from these studies in a scientifically sound and ethically responsible fashion in accordance with the unique nature of the IGNITE PTN mission. The IGNITE PTN CC will draft a PPC charter in collaboration with NHGRI, PPC, and the IGNITE PTN, which specifies the publication policies and procedures.

The sharing of datasets will be performed following standard operating procedures (SOPs) at the IGNITE PTN

CC, requirements for NIH policy for data sharing, and guidelines for NIH Data Set Preparation. The de-identified or anonymized data and documentation in standardized formats may be made available in dbGaP, ClinVar or other NHGRI-approved cloud based platforms for sharing to the larger scientific community. Requested unrestricted data may be made available after database lock and after all planned primary and secondary analyses have been published to parties who sign a data sharing agreement, which stipulates that data must be: 1) used solely for research purposes, 2) properly acknowledged in resulting publications, 3) kept confidential and inaccessible to third parties, and 4) destroyed or returned after analyses are completed. Additionally, users must agree not to use data to identify individual participants.

12. PROTOCOL DEVIATIONS

A protocol deviation is defined as an event where the Investigator or site personnel did not conduct the study according to the protocol or the Investigator Agreement. Investigators are required to obtain prior approval from the IGNITE PTN CC and the reviewing IRB before initiating deviations from the protocol, except where necessary to protect the life or physical well-being of a participant in an emergency. Such approval will be documented and maintained in study files. Prior approval is generally not expected in situations where unforeseen circumstances are beyond the Investigator's control, (e.g., participant did not attend scheduled follow-up visit, blood sample lost by laboratory, etc.); however, the event is still considered a deviation. Deviations will be reported to the IGNITE PTN CC and/or their designee regardless of whether medically justifiable, or taken to protect the participant in an emergency. Investigators will maintain documentation of the dates and reasons for each deviation from the protocol in compliance with the International Conference on Harmonisation Good Clinical Practice guidelines.

13. CONFLICT OF INTEREST

Institutions and investigators must report all present or potential financial conflicts of interest to the NIH, including any financial conflicts of interest identified in accordance with the 2011 42 CRF Part 50 Subpart F in the time and manner specified in the regulation. In addition, the DSMB membership is restricted to individuals free of significant or apparent conflicts of interest. The source of these conflicts may be financial, scientific or regulatory in nature. The IGNITE PTN CC will hold and update annually conflict-of-interest statements from each member. Any member who develops a potential conflict of interest during the course of the trial should inform other members of the DSMB. If the potential conflict of interest is determined to be substantial and relevant, the member should resign from the DSMB.

14. REFERENCES

1. Kazancioglu, R., *Risk factors for chronic kidney disease: an update.* Kidney Int Suppl (2011), 2013. **3**(4): p. 368-371.
2. Crews, D.C., T. Pfaff, and N.R. Powe, *Socioeconomic factors and racial disparities in kidney disease outcomes.* Semin Nephrol, 2013. **33**(5): p. 468-75.
3. Ayanian, J.Z., B.E. Landon, J.P. Newhouse, and A.M. Zaslavsky, *Racial and ethnic disparities among enrollees in Medicare Advantage plans.* N Engl J Med, 2014. **371**(24): p. 2288-97.
4. Gu, A., Y. Yue, R.P. Desai, and E. Argulian, *Racial and Ethnic Differences in Antihypertensive Medication Use and Blood Pressure Control Among US Adults With Hypertension: The National Health and Nutrition Examination Survey, 2003 to 2012.* Circ Cardiovasc Qual Outcomes, 2017. **10**(1).
5. Judd, E. and D.A. Calhoun, *Management of hypertension in CKD: beyond the guidelines.* Adv Chronic Kidney Dis, 2015. **22**(2): p. 116-22.
6. Taler, S.J., R. Agarwal, G.L. Bakris, J.T. Flynn, P.M. Nilsson, M. Rahman, P.W. Sanders, S.C. Textor, M.R. Weir, and R.R. Townsend, *KDOQI US commentary on the 2012 KDIGO clinical practice guideline for management of blood pressure in CKD.* Am J Kidney Dis, 2013. **62**(2): p. 201-13.
7. Upadhyay, A., A. Earley, S.M. Haynes, and K. Uhlig, *Systematic review: blood pressure target in chronic kidney disease and proteinuria as an effect modifier.* Ann Intern Med, 2011. **154**(8): p. 541-8.
8. Fryar, C.D., Y. Ostchega, C.M. Hales, G. Zhang, and D. Kruszon-Moran, *Hypertension Prevalence and Control Among Adults: United States, 2015-2016.* NCHS Data Brief, 2017(289): p. 1-8.
9. Parsa, A., W.H. Kao, D. Xie, B.C. Astor, M. Li, C.Y. Hsu, H.I. Feldman, R.S. Parekh, J.W. Kusek, T.H. Greene, J.C. Fink, A.H. Anderson, M.J. Choi, J.T. Wright, Jr., J.P. Lash, B.I. Freedman, A. Ojo, C.A. Winkler, D.S. Raj, J.B. Kopp, J. He, N.G. Jensvold, K. Tao, M.S. Lipkowitz, and L.J. Appel, *APOL1 risk variants, race, and progression of chronic kidney disease.* N Engl J Med, 2013. **369**(23): p. 2183-96.
10. Genovese, G., D.J. Friedman, M.D. Ross, L. Lecordier, P. Uzureau, B.I. Freedman, D.W. Bowden, C.D. Langefeld, T.K. Oleksyk, A.L. Uscinski Knob, A.J. Bernhardy, P.J. Hicks, G.W. Nelson, B. Vanhollebeke, C.A. Winkler, J.B. Kopp, E. Pays, and M.R. Pollak, *Association of trypanolytic ApoL1 variants with kidney disease in African Americans.* Science, 2010. **329**(5993): p. 841-5.
11. Foster, M.C., J. Coresh, M. Fornage, B.C. Astor, M. Grams, N. Franceschini, E. Boerwinkle, R.S. Parekh, and W.H. Kao, *APOL1 variants associate with increased risk of CKD among African Americans.* J Am Soc Nephrol, 2013. **24**(9): p. 1484-91.
12. Kern, E.F., M. Maney, D.R. Miller, C.L. Tseng, A. Tiwari, M. Rajan, D. Aron, and L. Pogach, *Failure of ICD-9-CM codes to identify patients with comorbid chronic kidney disease in diabetes.* Health Serv Res, 2006. **41**(2): p. 564-80.
13. Nadkarni, G.N., O. Gottesman, J.G. Linneman, H. Chase, R.L. Berg, S. Farouk, R. Nadukuru, V. Lotay, S. Ellis, G. Hripcsak, P. Peissig, C. Weng, and E.P. Bottinger, *Development and validation of an electronic phenotyping algorithm for chronic kidney disease.* AMIA Annu Symp Proc, 2014. **2014**: p. 907-16.
14. Omron Healthcare, I. *Professional Intellisense Blood Pressure Monitor*. 2019 [cited 2019; Available from: https://omronhealthcare.com/products/intellisense-professional-digital-blood-pressure-monitor-hem907xl/.
15. Abraham, J.E., M.J. Maranian, I. Spiteri, R. Russell, S. Ingle, C. Luccarini, H.M. Earl, P.P. Pharoah, A.M. Dunning, and C. Caldas, *Saliva samples are a viable alternative to blood samples as a source of DNA for high throughput genotyping.* BMC Med Genomics, 2012. **5**: p. 19.

16. Ettehad, D., C.A. Emdin, A. Kiran, S.G. Anderson, T. Callender, J. Emberson, J. Chalmers, A. Rodgers, and K. Rahimi, *Blood pressure lowering for prevention of cardiovascular disease and death: a systematic review and meta-analysis*. Lancet, 2016. **387**(10022): p. 957-967.
17. U.S. Centers for Medicare & Medicaid Services. *Physician Fee Schedule*. 2019; Available from: https://www.cms.gov/medicare/medicare-fee-for-service-payment/physicianfeesched/.
18. U.S. Department of Veterans Affairs. *National Acquisition Center (CCST) Pharmaceutical Catalog Search*. 2019; Available from: https://www.vendorportal.ecms.va.gov/NAC/Pharma/List.
19. Centers for Medicare & Medicaid Services. *NADAC (National Average Drug Acquisition Cost)*. 2019; Available from: https://data.medicaid.gov/Drug-Pricing-and-Payment/NADAC-National-Average-Drug-Acquisition-Cost-/a4y5-998d.
20. U.S. Centers for Medicare & Medicaid Services. *The Official U.S. Goverment site for Medicare*. 2019; Available from: https://www.medicare.gov/.
21. U.S. Centers for Medicare & Medicaid Services. *Hospital Outpatient PPS*. 2019; Available from: https://www.cms.gov/Medicare/Medicare-Fee-for-Service-Payment/HospitalOutpatientPPS/.
22. U.S. Centers for Medicare & Medicaid Services. *Cost Reports by Fiscal Year*. 2014; Available from: https://www.cms.gov/Research-Statistics-Data-and-Systems/Downloadable-Public-Use-Files/Cost-Reports/Cost-Reports-by-Fiscal-Year.html.
23. Sanders, G.D., P.J. Neumann, A. Basu, D.W. Brock, D. Feeny, M. Krahn, K.M. Kuntz, D.O. Meltzer, D.K. Owens, L.A. Prosser, J.A. Salomon, M.J. Sculpher, T.A. Trikalinos, L.B. Russell, J.E. Siegel, and T.G. Ganiats, *Recommendations for Conduct, Methodological Practices, and Reporting of Cost-effectiveness Analyses: Second Panel on Cost-Effectiveness in Health and Medicine*. Jama, 2016. **316**(10): p. 1093-103.
24. Goff, D.C., Jr., D.M. Lloyd-Jones, G. Bennett, S. Coady, R.B. D'Agostino, R. Gibbons, P. Greenland, D.T. Lackland, D. Levy, C.J. O'Donnell, J.G. Robinson, J.S. Schwartz, S.T. Shero, S.C. Smith, Jr., P. Sorlie, N.J. Stone, P.W. Wilson, H.S. Jordan, L. Nevo, J. Wnek, J.L. Anderson, J.L. Halperin, N.M. Albert, B. Bozkurt, R.G. Brindis, L.H. Curtis, D. DeMets, J.S. Hochman, R.J. Kovacs, E.M. Ohman, S.J. Pressler, F.W. Sellke, W.K. Shen, S.C. Smith, Jr., and G.F. Tomaselli, *2013 ACC/AHA guideline on the assessment of cardiovascular risk: a report of the American College of Cardiology/American Heart Association Task Force on Practice Guidelines*. Circulation, 2014. **129**(25 Suppl 2): p. S49-73.

15. APPENDICES

15.1 Appendix A. Diabetes Diagnosis Codes

Table 8. ICD10 diagnosis codes and descriptions for diabetes

ICD-10-CM DIABETES DIAGNOSIS CODES	DESCRIPTIONS
E08.00	Diabetes mellitus due to underlying condition with hyperosmolarity without nonketotic hyperglycemic-hyperosmolar coma (NKHHC)
E08.01	Diabetes mellitus due to underlying condition with hyperosmolarity with coma
E08.10	Diabetes mellitus due to underlying condition with ketoacidosis without coma
E08.11	Diabetes mellitus due to underlying condition with ketoacidosis with coma
E08.21	Diabetes mellitus due to underlying condition with diabetic nephropathy
E08.22	Diabetes mellitus due to underlying condition with diabetic chronic kidney disease
E08.29	Diabetes mellitus due to underlying condition with other diabetic kidney complication
E08.311	Diabetes mellitus due to underlying condition with unspecified diabetic retinopathy with macular edema
E08.3129	Diabetes mellitus due to underlying condition with mild nonproliferative diabetic retinopathy with macular edema, unspecified eye
E08.319	Diabetes mellitus due to underlying condition with unspecified diabetic retinopathy without macular edema
E08.3211	Diabetes mellitus due to underlying condition with mild nonproliferative diabetic retinopathy with macular edema, right eye
E08.3212	Diabetes mellitus due to underlying condition with mild nonproliferative diabetic retinopathy with macular edema, left eye
E08.3213	Diabetes mellitus due to underlying condition with mild nonproliferative diabetic retinopathy with macular edema, bilateral
E08.3291	Diabetes mellitus due to underlying condition with mild nonproliferative diabetic retinopathy without macular edema, right eye
E08.3292	Diabetes mellitus due to underlying condition with mild nonproliferative diabetic retinopathy without macular edema, left eye
E08.3293	Diabetes mellitus due to underlying condition with mild nonproliferative diabetic retinopathy without macular edema, bilateral
E08.3311	Diabetes mellitus due to underlying condition with moderate nonproliferative diabetic retinopathy with macular edema, right eye
E08.3312	Diabetes mellitus due to underlying condition with moderate nonproliferative diabetic retinopathy with macular edema, left eye

E08.3313	Diabetes mellitus due to underlying condition with moderate nonproliferative diabetic retinopathy with macular edema, bilateral
E08.3319	Diabetes mellitus due to underlying condition with moderate nonproliferative diabetic retinopathy with macular edema, unspecified eye
E08.3391	Diabetes mellitus due to underlying condition with moderate nonproliferative diabetic retinopathy without macular edema, right eye
E08.3392	Diabetes mellitus due to underlying condition with moderate nonproliferative diabetic retinopathy without macular edema, left eye
E08.3393	Diabetes mellitus due to underlying condition with moderate nonproliferative diabetic retinopathy without macular edema, bilateral
E08.3399	Diabetes mellitus due to underlying condition with moderate nonproliferative diabetic retinopathy without macular edema, unspecified eye
E08.3411	Diabetes mellitus due to underlying condition with severe nonproliferative diabetic retinopathy with macular edema, right eye
E08.3412	Diabetes mellitus due to underlying condition with severe nonproliferative diabetic retinopathy with macular edema, left eye
E08.3413	Diabetes mellitus due to underlying condition with severe nonproliferative diabetic retinopathy with macular edema, bilateral
E08.3419	Diabetes mellitus due to underlying condition with severe nonproliferative diabetic retinopathy with macular edema, unspecified eye
E08.3491	Diabetes mellitus due to underlying condition with severe nonproliferative diabetic retinopathy without macular edema, right eye
E08.3492	Diabetes mellitus due to underlying condition with severe nonproliferative diabetic retinopathy without macular edema, left eye
E08.3493	Diabetes mellitus due to underlying condition with severe nonproliferative diabetic retinopathy without macular edema, bilateral
E08.3499	Diabetes mellitus due to underlying condition with severe nonproliferative diabetic retinopathy without macular edema, unspecified eye
E08.351	Diabetes mellitus due to underlying condition with proliferative diabetic retinopathy with macular edema
E08.3511	Diabetes mellitus due to underlying condition with proliferative diabetic retinopathy with macular edema, right eye
E08.3512	Diabetes mellitus due to underlying condition with proliferative diabetic retinopathy with macular edema, left eye
E08.3513	Diabetes mellitus due to underlying condition with proliferative diabetic retinopathy with macular edema, bilateral
E08.3519	Diabetes mellitus due to underlying condition with proliferative diabetic retinopathy with macular edema, unspecified eye
E08.352	Diabetes mellitus due to underlying condition with proliferative diabetic retinopathy with traction retinal detachment involving the macula
E08.3521	Diabetes mellitus due to underlying condition with proliferative diabetic retinopathy with traction retinal detachment involving the macula, right eye

E08.3522	Diabetes mellitus due to underlying condition with proliferative diabetic retinopathy with traction retinal detachment involving the macula, left eye
E08.3523	Diabetes mellitus due to underlying condition with proliferative diabetic retinopathy with traction retinal detachment involving the macula, bilateral
E08.3529	Diabetes mellitus due to underlying condition with proliferative diabetic retinopathy with traction retinal detachment involving the macula, unspecified eye
E08.353	Diabetes mellitus due to underlying condition with proliferative diabetic retinopathy with traction retinal detachment not involving the macula
E08.3531	Diabetes mellitus due to underlying condition with proliferative diabetic retinopathy with traction retinal detachment not involving the macula, right eye
E08.3532	Diabetes mellitus due to underlying condition with proliferative diabetic retinopathy with traction retinal detachment not involving the macula, left eye
E08.3533	Diabetes mellitus due to underlying condition with proliferative diabetic retinopathy with traction retinal detachment not involving the macula, bilateral
E08.354	Diabetes mellitus due to underlying condition with proliferative diabetic retinopathy with combined traction retinal detachment and rhegmatogenous retinal detachment
E08.3541	Diabetes mellitus due to underlying condition with proliferative diabetic retinopathy with combined traction retinal detachment and rhegmatogenous retinal detachment, right eye
E08.3542	Diabetes mellitus due to underlying condition with proliferative diabetic retinopathy with combined traction retinal detachment and rhegmatogenous retinal detachment, left eye
E08.3543	Diabetes mellitus due to underlying condition with proliferative diabetic retinopathy with combined traction retinal detachment and rhegmatogenous retinal detachment, bilateral
E08.3549	Diabetes mellitus due to underlying condition with proliferative diabetic retinopathy with combined traction retinal detachment and rhegmatogenous retinal detachment, unspecified eye
E08.355	Diabetes mellitus due to underlying condition with stable proliferative diabetic retinopathy
E08.3551	Diabetes mellitus due to underlying condition with stable proliferative diabetic retinopathy, right eye
E08.3552	Diabetes mellitus due to underlying condition with stable proliferative diabetic retinopathy, left eye
E08.3553	Diabetes mellitus due to underlying condition with stable proliferative diabetic retinopathy, bilateral

E08.3559	Diabetes mellitus due to underlying condition with stable proliferative diabetic retinopathy, unspecified eye
E08.359	Diabetes mellitus due to underlying condition with proliferative diabetic retinopathy without macular edema
E08.3591	Diabetes mellitus due to underlying condition with proliferative diabetic retinopathy without macular edema, right eye
E08.3592	Diabetes mellitus due to underlying condition with proliferative diabetic retinopathy without macular edema, left eye
E08.3593	Diabetes mellitus due to underlying condition with proliferative diabetic retinopathy without macular edema, bilateral
E08.3599	Diabetes mellitus due to underlying condition with proliferative diabetic retinopathy without macular edema, unspecified eye
E08.36	Diabetes mellitus due to underlying condition with diabetic cataract
E08.37	Diabetes mellitus due to underlying condition with diabetic macular edema, resolved following treatment
E08.37X1	Diabetes mellitus due to underlying condition with diabetic macular edema, resolved following treatment, right eye
E08.37X2	Diabetes mellitus due to underlying condition with diabetic macular edema, resolved following treatment, left eye
E08.37X3	Diabetes mellitus due to underlying condition with diabetic macular edema, resolved following treatment, bilateral
E08.37X9	Diabetes mellitus due to underlying condition with diabetic macular edema, resolved following treatment, unspecified
E08.39	Diabetes mellitus due to underlying condition with other diabetic ophthalmic complication
E08.3939	Diabetes mellitus due to underlying condition with proliferative diabetic retinopathy with traction retinal detachment not involving the macula, unspecified eye
E08.40	Diabetes mellitus due to underlying condition with diabetic neuropathy, unspecified
E08.41	Diabetes mellitus due to underlying condition with diabetic mononeuropathy
E08.42	Diabetes mellitus due to underlying condition with diabetic polyneuropathy
E08.43	Diabetes mellitus due to underlying condition with diabetic autonomic (poly)neuropathy
E08.44	Diabetes mellitus due to underlying condition with diabetic amyotrophy
E08.49	Diabetes mellitus due to underlying condition with other diabetic neurological complication
E08.51	Diabetes mellitus due to underlying condition with diabetic peripheral angiopathy without gangrene
E08.52	Diabetes mellitus due to underlying condition with diabetic peripheral angiopathy with gangrene

E08.59	Diabetes mellitus due to underlying condition with other circulatory complications
E08.610	Diabetes mellitus due to underlying condition with diabetic arthropathy
E08.618	Diabetes mellitus due to underlying condition with other diabetic arthropathy
E08.620	Diabetes mellitus due to underlying condition with diabetic dermatitis
E08.621	Diabetes mellitus due to underlying condition with foot ulcer
E08.622	Diabetes mellitus due to underlying condition with other skin ulcer
E08.628	Diabetes mellitus due to underlying condition with other skin complications
E08.630	Diabetes mellitus due to underlying condition with periodontal disease
E08.638	Diabetes mellitus due to underlying condition with other oral complications
E08.641	Diabetes mellitus due to underlying condition with hypoglycemia with coma
E08.649	Diabetes mellitus due to underlying condition with hypoglycemia without coma
E08.65	Diabetes mellitus due to underlying condition with hyperglycemia
E08.69	Diabetes mellitus due to underlying condition with other specified complication
E08.8	Diabetes mellitus due to underlying condition with unspecified complication
E08.9	Diabetes mellitus due to underlying condition without complications
E09.00	Drug or chemical induced diabetes mellitus with hyperosmolarity without nonketotic hyperglycemic-hyperosmolar coma (NKHHC)
E09.01	Drug or chemical induced diabetes mellitus with hyperosmolarity with coma
E09.10	Drug or chemical induced diabetes mellitus with ketoacidosis without coma
E09.11	Drug or chemical induced diabetes mellitus with ketoacidosis with coma
E09.21	Drug or chemical induced diabetes mellitus with diabetic nephropathy
E09.22	Drug or chemical induced diabetes mellitus with diabetic chronic kidney disease
E09.29	Drug or chemical induced diabetes mellitus with other diabetic kidney complication
E09.311	Drug or chemical induced diabetes mellitus with unspecified diabetic retinopathy with macular edema
E09.319	Drug or chemical induced diabetes mellitus with unspecified diabetic retinopathy without macular edema
E09.3211	Drug or chemical induced diabetes mellitus with mild nonproliferative diabetic retinopathy with macular edema, right eye
E09.3212	Drug or chemical induced diabetes mellitus with mild nonproliferative diabetic retinopathy with macular edema, left eye
E09.3213	Drug or chemical induced diabetes mellitus with mild nonproliferative diabetic retinopathy with macular edema, bilateral
E09.3219	Drug or chemical induced diabetes mellitus with mild nonproliferative diabetic retinopathy with macular edema, unspecified eye
E09.3291	Drug or chemical induced diabetes mellitus with mild nonproliferative diabetic retinopathy without macular edema, right eye

E09.3292	Drug or chemical induced diabetes mellitus with mild nonproliferative diabetic retinopathy without macular edema, left eye
E09.3293	Drug or chemical induced diabetes mellitus with mild nonproliferative diabetic retinopathy without macular edema, bilateral
E09.3299	Drug or chemical induced diabetes mellitus with mild nonproliferative diabetic retinopathy without macular edema, unspecified eye
E09.3311	Drug or chemical induced diabetes mellitus with moderate nonproliferative diabetic retinopathy with macular edema, right eye
E09.3312	Drug or chemical induced diabetes mellitus with moderate nonproliferative diabetic retinopathy with macular edema, left eye
E09.3313	Drug or chemical induced diabetes mellitus with moderate nonproliferative diabetic retinopathy with macular edema, bilateral
E09.3319	Drug or chemical induced diabetes mellitus with moderate nonproliferative diabetic retinopathy with macular edema, unspecified eye
E09.3391	Drug or chemical induced diabetes mellitus with moderate nonproliferative diabetic retinopathy without macular edema, right eye
E09.3392	Drug or chemical induced diabetes mellitus with moderate nonproliferative diabetic retinopathy without macular edema, left eye
E09.3393	Drug or chemical induced diabetes mellitus with moderate nonproliferative diabetic retinopathy without macular edema, bilateral
E09.3399	Drug or chemical induced diabetes mellitus with moderate nonproliferative diabetic retinopathy without macular edema, unspecified eye
E09.3411	Drug or chemical induced diabetes mellitus with severe nonproliferative diabetic retinopathy with macular edema, right eye
E09.3412	Drug or chemical induced diabetes mellitus with severe nonproliferative diabetic retinopathy with macular edema, left eye
E09.3413	Drug or chemical induced diabetes mellitus with severe nonproliferative diabetic retinopathy with macular edema, bilateral
E09.3419	Drug or chemical induced diabetes mellitus with severe nonproliferative diabetic retinopathy with macular edema, unspecified eye
E09.3491	Drug or chemical induced diabetes mellitus with severe nonproliferative diabetic retinopathy without macular edema, right eye
E09.3492	Drug or chemical induced diabetes mellitus with severe nonproliferative diabetic retinopathy without macular edema, left eye
E09.3493	Drug or chemical induced diabetes mellitus with severe nonproliferative diabetic retinopathy without macular edema, bilateral
E09.3499	Drug or chemical induced diabetes mellitus with severe nonproliferative diabetic retinopathy without macular edema, unspecified eye
E09.3511	Drug or chemical induced diabetes mellitus with proliferative diabetic retinopathy with macular edema, right eye
E09.3512	Drug or chemical induced diabetes mellitus with proliferative diabetic retinopathy with macular edema, left eye

E09.3513	Drug or chemical induced diabetes mellitus with proliferative diabetic retinopathy with macular edema, bilateral
E09.3519	Drug or chemical induced diabetes mellitus with proliferative diabetic retinopathy with macular edema, unspecified eye
E09.3521	Drug or chemical induced diabetes mellitus with proliferative diabetic retinopathy with traction retinal detachment involving the macula, right eye
E09.3522	Drug or chemical induced diabetes mellitus with proliferative diabetic retinopathy with traction retinal detachment involving the macula, left eye
E09.3523	Drug or chemical induced diabetes mellitus with proliferative diabetic retinopathy with traction retinal detachment involving the macula, bilateral
E09.3529	Drug or chemical induced diabetes mellitus with proliferative diabetic retinopathy with traction retinal detachment involving the macula, unspecified eye
E09.3531	Drug or chemical induced diabetes mellitus with proliferative diabetic retinopathy with traction retinal detachment not involving the macula, right eye
E09.3532	Drug or chemical induced diabetes mellitus with proliferative diabetic retinopathy with traction retinal detachment not involving the macula, left eye
E09.3533	Drug or chemical induced diabetes mellitus with proliferative diabetic retinopathy with traction retinal detachment not involving the macula, bilateral
E09.3539	Drug or chemical induced diabetes mellitus with proliferative diabetic retinopathy with traction retinal detachment not involving the macula, unspecified eye
E09.3541	Drug or chemical induced diabetes mellitus with proliferative diabetic retinopathy with combined traction retinal detachment and rhegmatogenous retinal detachment, right eye
E09.3542	Drug or chemical induced diabetes mellitus with proliferative diabetic retinopathy with combined traction retinal detachment and rhegmatogenous retinal detachment, left eye
E09.3543	Drug or chemical induced diabetes mellitus with proliferative diabetic retinopathy with combined traction retinal detachment and rhegmatogenous retinal detachment, bilateral
E09.3549	Drug or chemical induced diabetes mellitus with proliferative diabetic retinopathy with combined traction retinal detachment and rhegmatogenous retinal detachment, unspecified eye
E09.3551	Drug or chemical induced diabetes mellitus with stable proliferative diabetic retinopathy, right eye
E09.3552	Drug or chemical induced diabetes mellitus with stable proliferative diabetic retinopathy, left eye

E09.3553	Drug or chemical induced diabetes mellitus with stable proliferative diabetic retinopathy, bilateral
E09.3559	Drug or chemical induced diabetes mellitus with stable proliferative diabetic retinopathy, unspecified eye
E09.3591	Drug or chemical induced diabetes mellitus with proliferative diabetic retinopathy without macular edema, right eye
E09.3592	Drug or chemical induced diabetes mellitus with proliferative diabetic retinopathy without macular edema, left eye
E09.3593	Drug or chemical induced diabetes mellitus with proliferative diabetic retinopathy without macular edema, bilateral
E09.3599	Drug or chemical induced diabetes mellitus with proliferative diabetic retinopathy without macular edema, unspecified eye
E09.36	Drug or chemical induced diabetes mellitus with diabetic cataract
E09.37X1	Drug or chemical induced diabetes mellitus with diabetic macular edema, resolved following treatment, right eye
E09.37X2	Drug or chemical induced diabetes mellitus with diabetic macular edema, resolved following treatment, left eye
E09.37X3	Drug or chemical induced diabetes mellitus with diabetic macular edema, resolved following treatment, bilateral
E09.37X9	Drug or chemical induced diabetes mellitus with diabetic macular edema, resolved following treatment, unspecified eye
E09.39	Drug or chemical induced diabetes mellitus with other diabetic ophthalmic complication
E09.40	Drug or chemical induced diabetes mellitus with neurological complications with diabetic neuropathy, unspecified
E09.41	Drug or chemical induced diabetes mellitus with neurological complications with diabetic mononeuropathy
E09.42	Drug or chemical induced diabetes mellitus with neurological complications with diabetic polyneuropathy
E09.43	Drug or chemical induced diabetes mellitus with neurological complications with diabetic autonomic (poly)neuropathy
E09.44	Drug or chemical induced diabetes mellitus with neurological complications with diabetic amyotrophy
E09.49	Drug or chemical induced diabetes mellitus with neurological complications with other diabetic neurologic complication
E09.51	Drug or chemical induced diabetes mellitus with diabetic peripheral angiopathy without gangrene
E09.52	Drug or chemical induced diabetes mellitus with diabetic peripheral angiopathy with gangrene
E09.59	Drug or chemical induced diabetes mellitus with other circulatory complications

E09.610	Drug or chemical induced diabetes mellitus with diabetic neuropathic arthropathy
E09.618	Drug or chemical induced diabetes mellitus with other diabetic arthropathy
E09.620	Drug or chemical induced diabetes mellitus with diabetic dermatitis
E09.621	Drug or chemical induced diabetes mellitus with foot ulcer
E09.622	Drug or chemical induced diabetes mellitus with other skin ulcer
E09.628	Drug or chemical induced diabetes mellitus with other skin complications
E09.630	Drug or chemical induced diabetes mellitus with periodontal disease
E09.638	Drug or chemical induced diabetes mellitus with other oral complications
E09.641	Drug or chemical induced diabetes mellitus with hypoglycemia with coma
E09.649	Drug or chemical induced diabetes mellitus with hypoglycemia without coma
E09.65	Drug or chemical induced diabetes mellitus with hyperglycemia
E09.69	Drug or chemical induced diabetes mellitus with other specified complication
E09.8	Drug or chemical induced diabetes mellitus with unspecified complications
E09.9	Drug or chemical induced diabetes mellitus without complications
E10.10	Type 1 diabetes mellitus with ketoacidosis without coma
E10.11	Type 1 diabetes mellitus with ketoacidosis with coma
E10.21	Type 1 diabetes mellitus with diabetic nephropathy
E10.22	Type 1 diabetes mellitus with diabetic chronic kidney disease
E10.29	Type 1 diabetes mellitus with other diabetic kidney complication
E10.311	Type 1 diabetes mellitus with unspecified diabetic retinopathy with macular edema
E10.319	Type 1 diabetes mellitus with unspecified diabetic retinopathy without macular edema
E10.3211	Type 1 diabetes mellitus with mild nonproliferative diabetic retinopathy with macular edema, right eye
E10.3212	Type 1 diabetes mellitus with mild nonproliferative diabetic retinopathy with macular edema, left eye
E10.3213	Type 1 diabetes mellitus with mild nonproliferative diabetic retinopathy with macular edema, bilateral
E10.3219	Type 1 diabetes mellitus with mild nonproliferative diabetic retinopathy with macular edema, unspecified eye
E10.3291	Type 1 diabetes mellitus with mild nonproliferative diabetic retinopathy without macular edema, right eye
E10.3292	Type 1 diabetes mellitus with mild nonproliferative diabetic retinopathy without macular edema, left eye
E10.3293	Type 1 diabetes mellitus with mild nonproliferative diabetic retinopathy without macular edema, bilateral
E10.3299	Type 1 diabetes mellitus with mild nonproliferative diabetic retinopathy without macular edema, unspecified eye
E10.3311	Type 1 diabetes mellitus with moderate nonproliferative diabetic retinopathy with macular edema, right eye

E10.3312	Type 1 diabetes mellitus with moderate nonproliferative diabetic retinopathy with macular edema, left eye
E10.3313	Type 1 diabetes mellitus with moderate nonproliferative diabetic retinopathy with macular edema, bilateral
E10.3319	Type 1 diabetes mellitus with moderate nonproliferative diabetic retinopathy with macular edema, unspecified eye
E10.3391	Type 1 diabetes mellitus with moderate nonproliferative diabetic retinopathy without macular edema, right eye
E10.3392	Type 1 diabetes mellitus with moderate nonproliferative diabetic retinopathy without macular edema, left eye
E10.3393	Type 1 diabetes mellitus with moderate nonproliferative diabetic retinopathy without macular edema, bilateral
E10.3399	Type 1 diabetes mellitus with moderate nonproliferative diabetic retinopathy without macular edema, unspecified eye
E10.3411	Type 1 diabetes mellitus with severe nonproliferative diabetic retinopathy with macular edema, right eye
E10.3412	Type 1 diabetes mellitus with severe nonproliferative diabetic retinopathy with macular edema, left eye
E10.3413	Type 1 diabetes mellitus with severe nonproliferative diabetic retinopathy with macular edema, bilateral
E10.3419	Type 1 diabetes mellitus with severe nonproliferative diabetic retinopathy with macular edema, unspecified eye
E10.3491	Type 1 diabetes mellitus with severe nonproliferative diabetic retinopathy without macular edema, right eye
E10.3492	Type 1 diabetes mellitus with severe nonproliferative diabetic retinopathy without macular edema, left eye
E10.3493	Type 1 diabetes mellitus with severe nonproliferative diabetic retinopathy without macular edema, bilateral
E10.3499	Type 1 diabetes mellitus with severe nonproliferative diabetic retinopathy without macular edema, unspecified eye
E10.3511	Type 1 diabetes mellitus with proliferative diabetic retinopathy with macular edema, right eye
E10.3512	Type 1 diabetes mellitus with proliferative diabetic retinopathy with macular edema, left eye
E10.3513	Type 1 diabetes mellitus with proliferative diabetic retinopathy with macular edema, bilateral
E10.3519	Type 1 diabetes mellitus with proliferative diabetic retinopathy with macular edema, unspecified eye
E10.3521	Type 1 diabetes mellitus with proliferative diabetic retinopathy with traction retinal detachment involving the macula, right eye
E10.3522	Type 1 diabetes mellitus with proliferative diabetic retinopathy with traction retinal detachment involving the macula, left eye

E10.3523	Type 1 diabetes mellitus with proliferative diabetic retinopathy with traction retinal detachment involving the macula, bilateral
E10.3529	Type 1 diabetes mellitus with proliferative diabetic retinopathy with traction retinal detachment involving the macula, unspecified eye
E10.3531	Type 1 diabetes melliuts with proliferative diabetic retinopathy with traction rentinal detachment not involving the macula, right eye
E10.3532	Type 1 diabetes melliuts with proliferative diabetic retinopathy with traction rentinal detachment not involving the macula, left eye
E10.3533	Type 1 diabetes melliuts with proliferative diabetic retinopathy with traction rentinal detachment not involving the macula, bilateral
E10.3539	Type 1 diabetes melliuts with proliferative diabetic retinopathy with traction rentinal detachment not involving the macula, unspecified eye
E10.3541	Type 1 diabetes mellitus with proliferative diabetic retinopathy with combined traction retinal detachment and rhegmatogenous retinal detachment, right eye
E10.3542	Type 1 diabetes mellitus with proliferative diabetic retinopathy with combined traction retinal detachment and rhegmatogenous retinal detachment, left eye
E10.3543	Type 1 diabetes mellitus with proliferative diabetic retinopathy with combined traction retinal detachment and rhegmatogenous retinal detachment, bilateral
E10.3549	Type 1 diabetes mellitus with proliferative diabetic retinopathy with combined traction retinal detachment and rhegmatogenous retinal detachment, unspecified eye
E10.3551	Type 1 diabetes mellitus with stable proliferative diabetic retinopathy, right eye
E10.3552	Type 1 diabetes mellitus with stable proliferative diabetic retinopathy, left eye
E10.3553	Type 1 diabetes mellitus with stable proliferative diabetic retinopathy, bilateral
E10.3559	Type 1 diabetes mellitus with stable proliferative diabetic retinopathy, unspecified eye
E10.3591	Type 1 diabetes mellitus with proliferative diabetic retinopathy without macular edema, right eye
E10.3592	Type 1 diabetes mellitus with proliferative diabetic retinopathy without macular edema, left eye
E10.3593	Type 1 diabetes mellitus with proliferative diabetic retinopathy without macular edema, bilateral
E10.3599	Type 1 diabetes mellitus with proliferative diabetic retinopathy without macular edema, unspecified eye
E10.36	Type 1 diabetes mellitus with diabetic cataract

E10.37X1	Type 1 diabetes mellitus with diabetic macular edema, resolved following treatment, right eye
E10.37X2	Type 1 diabetes mellitus with diabetic macular edema, resolved following treatment, left eye
E10.37X3	Type 1 diabetes mellitus with diabetic macular edema, resolved following treatment, bilateral
E10.37X9	Type 1 diabetes mellitus with diabetic macular edema, resolved following treatment, unspecified eye
E10.39	Type 1 diabetes mellitus with other diabetic ophthalmic complication
E10.40	Type 1 diabetes mellitus with diabetic neuropathy, unspecified
E10.41	Type 1 diabetes mellitus with diabetic mononeuropathy
E10.42	Type 1 diabetes mellitus with diabetic polyneuropathy
E10.43	Type 1 diabetes mellitus with diabetic autonomic (poly)neuropathy
E10.44	Type 1 diabetes mellitus with diabetic amyotrophy
E10.49	Type 1 diabetes mellitus with other diabetic neurological complication
E10.51	Type 1 diabetes mellitus with diabetic peripheral angiopathy without gangrene
E10.52	Type 1 diabetes mellitus with diabetic peripheral angiopathy with gangrene
E10.59	Type 1 diabetes mellitus with other circulatory complications
E10.610	Type 1 diabetes mellitus with diabetic neuropathic arthropathy
E10.618	Type 1 diabetes mellitus with other diabetic arthropathy
E10.620	Type 1 diabetes mellitus with diabetic dermatitis
E10.621	Type 1 diabetes mellitus with foot ulcer
E10.622	Type 1 diabetes mellitus with other skin ulcer
E10.628	Type 1 diabetes mellitus with other skin complications
E10.630	Type 1 diabetes mellitus with periodontal disease
E10.638	Type 1 diabetes mellitus with other oral complications
E10.641	Type 1 diabetes mellitus with hypoglycemia with coma
E10.649	Type 1 diabetes mellitus with hypoglycemia without coma
E10.65	Type 1 diabetes mellitus with hyperglycemia
E10.69	Type 1 diabetes mellitus with other specified complication
E10.8	Type 1 diabetes mellitus with unspecified complications
E10.9	Type 1 diabetes mellitus without complications
E11.00	Type 2 diabetes mellitus with hyperosmolarity without nonketotic hyperglycemic-hyperosmolar coma (NKHHC)
E11.01	Type 2 diabetes mellitus with hyperosmolarity with coma
E11.10	Type 2 diabetes mellitus with ketoacidosis without coma
E11.11	Type 2 diabetes mellitus with ketoacidosis with coma
E11.21	Type 2 diabetes mellitus with diabetic nephropathy
E11.22	Type 2 diabetes mellitus with diabetic chronic kidney disease
E11.29	Type 2 diabetes mellitus with other diabetic kidney complication

E11.311	Type 2 diabetes mellitus with unspecified diabetic retinopathy with macular edema
E11.319	Type 2 diabetes mellitus with unspecified diabetic retinopathy without macular edema
E11.3211	Type 2 diabetes mellitus with mild nonproliferative diabetic retinopathy with macular edema, right eye
E11.3212	Type 2 diabetes mellitus with mild nonproliferative diabetic retinopathy with macular edema, left eye
E11.3213	Type 2 diabetes mellitus with mild nonproliferative diabetic retinopathy with macular edema, bilateral
E11.3219	Type 2 diabetes mellitus with mild nonproliferative diabetic retinopathy with macular edema, unspecified eye
E11.3291	Type 2 diabetes mellitus with mild nonproliferative diabetic retinopathy without macular edema, right eye
E11.3292	Type 2 diabetes mellitus with mild nonproliferative diabetic retinopathy without macular edema, left eye
E11.3293	Type 2 diabetes mellitus with mild nonproliferative diabetic retinopathy without macular edema, bilateral
E11.3299	Type 2 diabetes mellitus with mild nonproliferative diabetic retinopathy without macular edema, unspecified eye
E11.3311	Type 2 diabetes mellitus with moderate nonproliferative diabetic retinopathy with macular edema, right eye
E11.3312	Type 2 diabetes mellitus with moderate nonproliferative diabetic retinopathy with macular edema, left eye
E11.3313	Type 2 diabetes mellitus with moderate nonproliferative diabetic retinopathy with macular edema, bilateral
E11.3319	Type 2 diabetes mellitus with moderate nonproliferative diabetic retinopathy with macular edema, unspecified eye
E11.3391	Type 2 diabetes mellitus with moderate nonproliferative diabetic retinopathy without macular edema, right eye
E11.3392	Type 2 diabetes mellitus with moderate nonproliferative diabetic retinopathy without macular edema, left eye
E11.3393	Type 2 diabetes mellitus with moderate nonproliferative diabetic retinopathy without macular edema, bilateral
E11.3399	Type 2 diabetes mellitus with moderate nonproliferative diabetic retinopathy without macular edema, unspecified eye
E11.3411	Type 2 diabetes mellitus with severe nonproliferative diabetic retinopathy with macular edema, right eye
E11.3412	Type 2 diabetes mellitus with severe nonproliferative diabetic retinopathy with macular edema, left eye
E11.3413	Type 2 diabetes mellitus with severe nonproliferative diabetic retinopathy with macular edema, bilateral

E11.3419	Type 2 diabetes mellitus with severe nonproliferative diabetic retinopathy with macular edema, unspecified eye
E11.3491	Type 2 diabetes mellitus with severe nonproliferative diabetic retinopathy without macular edema, right eye
E11.3492	Type 2 diabetes mellitus with severe nonproliferative diabetic retinopathy without macular edema, left eye
E11.3493	Type 2 diabetes mellitus with severe nonproliferative diabetic retinopathy without macular edema, bilateral
E11.3499	Type 2 diabetes mellitus with severe nonproliferative diabetic retinopathy without macular edema, unspecified eye
E11.3511	Type 2 diabetes mellitus with proliferative diabetic retinopathy with macular edema, right eye
E11.3512	Type 2 diabetes mellitus with proliferative diabetic retinopathy with macular edema, left eye
E11.3513	Type 2 diabetes mellitus with proliferative diabetic retinopathy with macular edema, bilateral
E11.3519	Type 2 diabetes mellitus with proliferative diabetic retinopathy with macular edema, unspecified eye
E11.3521	Type 2 diabetes mellitus with proliferative diabetic retinopathy with traction retinal detachment involving the macula, right eye
E11.3522	Type 2 diabetes mellitus with proliferative diabetic retinopathy with traction retinal detachment involving the macula, left eye
E11.3523	Type 2 diabetes mellitus with proliferative diabetic retinopathy with traction retinal detachment involving the macula, bilateral
E11.3529	Type 2 diabetes mellitus with proliferative diabetic retinopathy with traction retinal detachment involving the macula, unspecified eye
E11.3531	Type 2 diabetes mellitus with proliferative diabetic retinopathy with traction retinal detachment not involving the macula, right eye
E11.3532	Type 2 diabetes mellitus with proliferative diabetic retinopathy with traction retinal detachment not involving the macula, left eye
E11.3533	Type 2 diabetes mellitus with proliferative diabetic retinopathy with traction retinal detachment not involving the macula, bilateral
E11.3539	Type 2 diabetes mellitus with proliferative diabetic retinopathy with traction retinal detachment not involving the macula, unspecified eye
E11.3541	Type 2 diabetes mellitus with proliferative diabetic retinopathy with combined traction retinal detachment and rhegmatogenous retinal detachment, right eye
E11.3542	Type 2 diabetes mellitus with proliferative diabetic retinopathy with combined traction retinal detachment and rhegmatogenous retinal detachment, left eye

E11.3543	Type 2 diabetes mellitus with proliferative diabetic retinopathy with combined traction retinal detachment and rhegmatogenous retinal detachment, bilateral
E11.3549	Type 2 diabetes mellitus with proliferative diabetic retinopathy with combined traction retinal detachment and rhegmatogenous retinal detachment, unspecified eye
E11.3551	Type 2 diabetes mellitus with stable proliferative diabetic retinopathy, right eye
E11.3552	Type 2 diabetes mellitus with stable proliferative diabetic retinopathy, left eye
E11.3553	Type 2 diabetes mellitus with stable proliferative diabetic retinopathy, bilateral
E11.3559	Type 2 diabetes mellitus with stable proliferative diabetic retinopathy, unspecified eye
E11.3591	Type 2 diabetes mellitus with proliferative diabetic retinopathy without macular edema, right eye
E11.3592	Type 2 diabetes mellitus with proliferative diabetic retinopathy without macular edema, left eye
E11.3593	Type 2 diabetes mellitus with proliferative diabetic retinopathy without macular edema, bilateral
E11.3599	Type 2 diabetes mellitus with proliferative diabetic retinopathy without macular edema, unspecified eye
E11.36	Type 2 diabetes mellitus with diabetic cataract
E11.37X1	Type 2 diabetes mellitus with diabetic macular edema, resolved following treatment, right eye
E11.37X2	Type 2 diabetes mellitus with diabetic macular edema, resolved following treatment, left eye
E11.37X3	Type 2 diabetes mellitus with diabetic macular edema, resolved following treatment, bilateral
E11.37X9	Type 2 diabetes mellitus with diabetic macular edema, resolved following treatment, unspecified eye
E11.39	Type 2 diabetes mellitus with other diabetic ophthalmic complication
E11.40	Type 2 diabetes mellitus with diabetic neuropathy, unspecified
E11.41	Type 2 diabetes mellitus with diabetic mononeuropathy
E11.42	Type 2 diabetes mellitus with diabetic polyneuropathy
E11.43	Type 2 diabetes mellitus with diabetic autonomic (poly)neuropathy
E11.44	Type 2 diabetes mellitus with diabetic amyotrophy
E11.49	Type 2 diabetes mellitus with other diabetic neurological complication
E11.51	Type 2 diabetes mellitus with diabetic peripheral angiopathy without gangrene
E11.52	Type 2 diabetes mellitus with diabetic peripheral angiopathy with gangrene
E11.59	Type 2 diabetes mellitus with other circulatory complications

E11.610	Type 2 diabetes mellitus with diabetic neuropathic arthropathy
E11.618	Type 2 diabetes mellitus with other diabetic arthropathy
E11.620	Type 2 diabetes mellitus with diabetic dermatitis
E11.621	Type 2 diabetes mellitus with foot ulcer
E11.622	Type 2 diabetes mellitus with other skin ulcer
E11.628	Type 2 diabetes mellitus with other skin complications
E11.630	Type 2 diabetes mellitus with periodontal disease
E11.638	Type 2 diabetes mellitus with other oral complications
E11.641	Type 2 diabetes mellitus with hypoglycemia with coma
E11.649	Type 2 diabetes mellitus with hypoglycemia without coma
E11.65	Type 2 diabetes mellitus with hyperglycemia
E11.69	Type 2 diabetes mellitus with other specified complication
E11.8	Type 2 diabetes mellitus with unspecified complications
E11.9	Type 2 diabetes mellitus without complications
E13.00	Other specified diabetes mellitus with hyperosmolarity without nonketotic hyperglycemic-hyperosmolar coma (NKHHC)
E13.01	Other specified diabetes mellitus with hyperosmolarity with coma
E13.10	Other specified diabetes mellitus with ketoacidosis without coma
E13.11	Other specified diabetes mellitus with ketoacidosis with coma
E13.21	Other specified diabetes mellitus with diabetic nephropathy
E13.22	Other specified diabetes mellitus with diabetic chronic kidney disease
E13.29	Other specified diabetes mellitus with other diabetic kidney complication
E13.311	Other specified diabetes mellitus with unspecified diabetic retinopathy with macular edema
E13.319	Other specified diabetes mellitus with unspecified diabetic retinopathy without macular edema
E13.3211	Other specified diabetes mellitus with mild nonproliferative diabetic retinopathy with macular edema, right eye
E13.3212	Other specified diabetes mellitus with mild nonproliferative diabetic retinopathy with macular edema, left eye
E13.3213	Other specified diabetes mellitus with mild nonproliferative diabetic retinopathy with macular edema, bilateral
E13.3219	Other specified diabetes mellitus with mild nonproliferative diabetic retinopathy with macular edema, unspecified eye
E13.3291	Other specified diabetes mellitus with mild nonproliferative diabetic retinopathy without macular edema, right eye
E13.3292	Other specified diabetes mellitus with mild nonproliferative diabetic retinopathy without macular edema, left eye
E13.3293	Other specified diabetes mellitus with mild nonproliferative diabetic retinopathy without macular edema, bilateral
E13.3299	Other specified diabetes mellitus with mild nonproliferative diabetic retinopathy without macular edema, unspecified eye

E13.3311	Other specified diabetes mellitus with moderate nonproliferative diabetic retinopathy with macular edema, right eye
E13.3312	Other specified diabetes mellitus with moderate nonproliferative diabetic retinopathy with macular edema, left eye
E13.3313	Other specified diabetes mellitus with moderate nonproliferative diabetic retinopathy with macular edema, bilateral
E13.3319	Other specified diabetes mellitus with moderate nonproliferative diabetic retinopathy with macular edema, unspecified eye
E13.3391	Other specified diabetes mellitus with moderate nonproliferative diabetic retinopathy without macular edema, right eye
E13.3392	Other specified diabetes mellitus with moderate nonproliferative diabetic retinopathy without macular edema, left eye
E13.3393	Other specified diabetes mellitus with moderate nonproliferative diabetic retinopathy without macular edema, bilateral
E13.3399	Other specified diabetes mellitus with moderate nonproliferative diabetic retinopathy without macular edema, unspecified eye
E13.3411	Other specified diabetes mellitus with severe nonproliferative diabetic retinopathy with macular edema, right eye
E13.3412	Other specified diabetes mellitus with severe nonproliferative diabetic retinopathy with macular edema, left eye
E13.3413	Other specified diabetes mellitus with severe nonproliferative diabetic retinopathy with macular edema, bilateral
E13.3419	Other specified diabetes mellitus with severe nonproliferative diabetic retinopathy with macular edema, unspecified eye
E13.3491	Other specified diabetes mellitus with severe nonproliferative diabetic retinopathy without macular edema, right eye
E13.3492	Other specified diabetes mellitus with severe nonproliferative diabetic retinopathy without macular edema, left eye
E13.3493	Other specified diabetes mellitus with severe nonproliferative diabetic retinopathy without macular edema, bilateral
E13.3499	Other specified diabetes mellitus with severe nonproliferative diabetic retinopathy without macular edema, unspecified eye
E13.3511	Other specified diabetes mellitus with proliferative diabetic retinopathy with macular edema, right eye
E13.3512	Other specified diabetes mellitus with proliferative diabetic retinopathy with macular edema, left eye
E13.3513	Other specified diabetes mellitus with proliferative diabetic retinopathy with macular edema, bilateral
E13.3519	Other specified diabetes mellitus with proliferative diabetic retinopathy with macular edema, unspecified eye
E13.3521	Other specified diabetes mellitus with proliferative diabetic retinopathy with traction retinal detachment involving the macula, right eye

E13.3522	Other specified diabetes mellitus with proliferative diabetic retinopathy with traction retinal detachment involving the macula, left eye
E13.3523	Other specified diabetes mellitus with proliferative diabetic retinopathy with traction retinal detachment involving the macula, bilateral
E13.3529	Other specified diabetes mellitus with proliferative diabetic retinopathy with traction retinal detachment involving the macula, unspecified eye
E13.3531	Other specified diabetes mellitus with proliferative diabetic retinopathy with traction retinal detachment not involving the macula, right eye
E13.3532	Other specified diabetes mellitus with proliferative diabetic retinopathy with traction retinal detachment not involving the macula, left eye
E13.3533	Other specified diabetes mellitus with proliferative diabetic retinopathy with traction retinal detachment not involving the macula, bilateral
E13.3539	Other specified diabetes mellitus with proliferative diabetic retinopathy with traction retinal detachment not involving the macula, unspecified eye
E13.3541	Other specified diabetes mellitus with proliferative diabetic retinopathy with combined traction retinal detachment and rhegmatogenous retinal detachment, right eye
E13.3542	Other specified diabetes mellitus with proliferative diabetic retinopathy with combined traction retinal detachment and rhegmatogenous retinal detachment, left eye
E13.3543	Other specified diabetes mellitus with proliferative diabetic retinopathy with combined traction retinal detachment and rhegmatogenous retinal detachment, bilateral
E13.3549	Other specified diabetes mellitus with proliferative diabetic retinopathy with combined traction retinal detachment and rhegmatogenous retinal detachment, unspecified eye
E13.3551	Other specified diabetes mellitus with stable proliferative diabetic retinopathy, right eye
E13.3552	Other specified diabetes mellitus with stable proliferative diabetic retinopathy, left eye
E13.3553	Other specified diabetes mellitus with stable proliferative diabetic retinopathy, bilateral
E13.3559	Other specified diabetes mellitus with stable proliferative diabetic retinopathy, unspecified eye
E13.3591	Other specified diabetes mellitus with proliferative diabetic retinopathy without macular edema, right eye
E13.3592	Other specified diabetes mellitus with proliferative diabetic retinopathy without macular edema, left eye
E13.3593	Other specified diabetes mellitus with proliferative diabetic retinopathy without macular edema, bilateral
E13.3599	Other specified diabetes mellitus with proliferative diabetic retinopathy without macular edema, unspecified eye

E13.36	Other specified diabetes mellitus with diabetic cataract
E13.37X1	Other specified diabetes mellitus with diabetic macular edema, resolved following treatment, right eye
E13.37X2	Other specified diabetes mellitus with diabetic macular edema, resolved following treatment, left eye
E13.37X3	Other specified diabetes mellitus with diabetic macular edema, resolved following treatment, bilateral
E13.37X9	Other specified diabetes mellitus with diabetic macular edema, resolved following treatment, unspecified eye
E13.39	Other specified diabetes mellitus with other diabetic ophthalmic complication
E13.40	Other specified diabetes mellitus with diabetic neuropathy, unspecified
E13.41	Other specified diabetes mellitus with diabetic mononeuropathy
E13.42	Other specified diabetes mellitus with diabetic polyneuropathy
E13.43	Other specified diabetes mellitus with diabetic autonomic (poly)neuropathy
E13.44	Other specified diabetes mellitus with diabetic amyotrophy
E13.49	Other specified diabetes mellitus with other diabetic neurological complication
E13.51	Other specified diabetes mellitus with diabetic peripheral angiopathy without gangrene
E13.52	Other specified diabetes mellitus with diabetic peripheral angiopathy with gangrene
E13.59	Other specified diabetes mellitus with other circulatory complications
E13.610	Other specified diabetes mellitus with diabetic neuropathic arthropathy
E13.618	Other specified diabetes mellitus with other diabetic arthropathy
E13.620	Other specified diabetes mellitus with diabetic dermatitis
E13.621	Other specified diabetes mellitus with foot ulcer
E13.622	Other specified diabetes mellitus with other skin ulcer
E13.628	Other specified diabetes mellitus with other skin complications
E13.630	Other specified diabetes mellitus with periodontal disease
E13.638	Other specified diabetes mellitus with other oral complications
E13.641	Other specified diabetes mellitus with hypoglycemia with coma
E13.649	Other specified diabetes mellitus with hypoglycemia without coma
E13.65	Other specified diabetes mellitus with hyperglycemia
E13.69	Other specified diabetes mellitus with other specified complication
E13.8	Other specified diabetes mellitus with unspecified complications
E13.9	Other specified diabetes mellitus without complications
E8.3299	Diabetes mellitus due to underlying condition with mild nonproliferative diabetic retinopathy without macular edema, unspecified eye
O24.410	Gestational diabetes mellitus in pregnancy, diet controlled
O24.414	Gestational diabetes mellitus in pregnancy, insulin controlled

O24.415	Gestational diabetes mellitus in pregnancy controlled by oral hypoglycemic drugs
O24.419	Gestational diabetes mellitus in pregnancy unspecified control
O24.420	Gestational diabetes mellitus in childbirth, diet controlled
O24.424	Gestational diabetes mellitus in childbirth, insulin controlled
O24.425	Gestational diabetes mellitus in childbirth, controlled by oral hypoglycemic drugs
O24.429	Gestational diabetes mellitus in childbirth, unspecified control
O24.430	Gestational diabetes mellitus in the puerperium, diet controlled
O24.434	Gestational diabetes mellitus in the puerperium, insulin controlled
O24.435	Gestational diabetes mellitus in the puerperium, controlled by oral hypoglycemic drugs
O24.439	Gestational diabetes mellitus in the puerperium, unspecified control

15.2

15.2 Appendix B. PGx Substudy Gene Description and Evidence

Overview: The PGx sub-study will examine the efficacy of providing genetic information for two gene-drug pairs. The first gene-drug pair is *YEATS4* and thiazide diuretic efficacy, impacting first-line antihypertensive therapy in African Americans. The second gene-drug pair is *NAT2* and hydralazine. Hydralazine is not first line therapy, but is used in the treatment of resistant hypertension. GUARDD-US will include patients with both hypertension and resistant hypertension. Neither gene-drug pair is presently recommended by CPIC, but both have significant evidence supporting their relationship in populations that include individuals of African ancestry. Given that 83% of the GUARDD-US intervention arm will be *APOL1* negative, the inclusion of a HTN PGx sub-study is an economical means of testing a second hypothesis.

Most individuals with hypertension require 2 or more agents to control blood pressure and those with resistant hypertension may require 4 or 5 agents. In contrast to other current PGx recommendations (e.g. antidepressants or opioids), antihypertensive agents are often not used in isolation. Thus, recommendations need to convey both dosing suggestions and/or relative efficacy to enable a provider to efficiently craft a regimen with fewer agents. This is of even greater importance in individuals with resistant hypertension.

YEATS4:

Evidence: The first gene-drug pair is *YEATS4*, predictive of thiazide efficacy in two independent cohorts. This gene-drug pair was associated with efficacy of thiazide diuretics (HCTZ) in the GERA and PEAR cohorts. It was discovered in a genome wide association of African American GERA patients (ST Turner) and later replicated in the PEAR cohort (JD Duarte). The SNP, rs7297610, is an expression quantitative locus of *YEATS4*, as expression is associated with both genotype and thiazide therapy. Citations are provided below. Although both GERA and PEAR studied HCTZ, we have decided to relay recommendations as a class effect (including both HCTZ and chlorthalidone). The rationale for this decision include the following: 1) the mechanisms of action are the same, 2) GUARDD-US is a pragmatic trial and the inclusion of the class effect enhances future generalizability, and 3) GUARDD-US includes individuals with CKD in whom chlorthalidone will be preferentially used by practitioners. We recognize hydrochlorothiazide (6-15 hours) and chlorthalidone (45-60 hours) each have a very different half-life. Overall, we feel the advantages of studying both drugs outweigh the potential heterogeneity. Further, we also note that the YEATS4 SNP is an expression quantitative loci and is anticipated to have a greater pharmacodynamic effect than pharmacokinetic effect.

Effect size: The effect size of rs7297610 is large. The T allele confers reduced efficacy to thiazide diuretics, reducing change in SBP by 3.4 mmHg and change in DBP by 2.5 mmHg. Stated another way, individuals with a TC or TT genotype are expected to have a 9.6 mmHg reduction in SBP in response to HCTZ initiation. Individuals with a CC genotype are expected to have a 13.1 mmHg reduction in SBP in response to HCTZ initiation.

Frequency: The minor allele frequency is ideal for testing effect size. The minor allele frequency of the T allele is ~0.3 with a dominant model for reduced efficacy. This means that approximately ½ of individuals (9% TT and 42% CT) would be expected to have reduced efficacy and ½ of individuals (49% CC) would

be expected to have increased/standard efficacy. Thus, ~1 in 2 individuals would have an actionable genotype.

Recommendation: First line antihypertensive therapy for African Americans presently consists of either a calcium channel blocker or thiazide diuretic. Our recommendation focusses on selection of first line therapy. The PEAR study included HCTZ and atenolol. Beta-blockers are no longer first line antihypertensive therapy. Thus, although HCTZ was not directly compared to a CCB in the study, we still have evidence that the T allele reduces thiazide efficacy. Thus, we are recommending thiazide or CCB use as the first drug in those with a CC genotype, but only a CCB as first-line in those with a T allele. This recommendation is pragmatic and straightforward for clinicians. The GUARDD-US trial will help us to test the efficacy of this recommendation.

Our recommendation does not include specific dosing recommendations for thiazide diuretics, only relative efficacy recommendations. The rationale for this decision is that there is limited dynamic range of thiazide efficacy (essentially 12.5 mg or 25 mg dosing) and in practice, many clinicians will begin therapy at 25 mg daily. The GERA trial included only one dose, 25 mg. The PEAR trial included a titration protocol, so the observed effect is a mix of the 12.5 mg and 25 mg dose. Thus, there is insufficient data to conclude a dose-effect relationship.

Goal (Drug Class)	Gene	Genotype	Recommendation
First Line Therapy (Thiazide diuretic)	*YEATS4*	C/C	This individual is predicted to have standard thiazide efficacy. Consider use of a thiazide diuretic or calcium channel blocker as first line treatment of hypertension.
First Line Therapy (Thiazide diuretic)	*YEATS4*	TT or TC	This individual is predicted to have reduced thiazide efficacy. Consider use of an alternate first line therapy such as a calcium channel blocker for treatment of hypertension.
Resistant Hypertension (Hydralazine)	*NAT2*	*4/*X	This individuals is predicted to have reduced hydralazine efficacy and may require a higher starting dose or an alternate agent such as clonidine, spironolactone, or doxazosin for resistant hypertension.
Resistant Hypertension (Hydralazine)	*NAT2*	2 non-*4 alleles	This individual is predicted to have increased efficacy of hydralazine.

NAT2 Evidence:

Evidence: The second gene-drug pair selected was *NAT2* and hydralazine. Hydralazine is metabolized by *NAT2*, so poor metabolizers have increased circulating concentrations of hydralazine. In this situation, poor metabolizers and slow acetylators terms are synonymous. This relationship was first reported nearly

50 years ago. PharmGKB presently lists hydralzine-*NAT2* as a level 3 recommendation, but there is only 1 reference cited in PharmGKB. There is actually a preponderance of evidence (21 references) supporting the relationship between: 1) metabolizer status to hydralazine PK or blood pressure effect, 2) Metabolizer status to genotype, or 3) genotype directly to blood pressure effect. We also identified 3 negative studies, but in each there were limitations (underpowered or included multiple drugs which confounded the effect). Please see the references below. In summary, the level of evidence for this drug-gene pair could potentially support a future CPIC recommendation. *NAT2* metabolism is also listed in the FDA drug label for hydralazine. The evidence includes data in African ancestry populations and the strongest effect is seen in slow acetylators/poor metabolizers. Based on evidence, it is possible that there is an intermediate phenotype, but the evidence is insufficient to distinguish blood pressure effects in rapid and intermediate acetylators.

Effect size: The effect size of the acetylator phenotype is large. Poor metabolizers may require a dose adjustment of 40% to >100% based on metabolizer status. There is a wide dose prescribing range for hydralazine from 10 mg BID to 100 mg TID.

Frequency: The allele frequency is ideal for testing effect size. In the African American population, 35% are intermediate (1 non-*4 allele) and ~50% are slow acetylators (possessing 2 non-*4 alleles). Thus, ~1 in 2 individuals would have an actionable genotype.

Recommendation: Based on the multitude of studies, we are relaying a climinical implication that poor metabolizers may have higher circulating concentrations with increased efficacy or risk for adverse effects. Normal/rapid metabolizers may require a higher dose of hydralazine or an alternate agent as compared to poor metabolizers (slow acetylators) to achieve equivalent blood pressure control.

Thiazide and *YEATS4* evidence

1. Shahin MH, Johnson JA. Mechanisms and pharmacogenetic signals underlying thiazide diuretics blood pressure response. Curr Opin Pharmacol. 2016 Apr;27:31-7. PMID: 26874237

2. Duarte JD, Turner ST, Tran B, Chapman AB, Bailey KR, Gong Y, Gums JG, Langaee TY, Beitelshees AL, Cooper-Dehoff RM, Boerwinkle E, Johnson JA. Association of chromosome 12 locus with

antihypertensive response to hydrochlorothiazide may involve differential YEATS4 expression. Pharmacogenomics J. 2013 Jun;13(3):257-63. Feb 21. PMID: 22350108

3. Turner ST, Bailey KR, Fridley BL, Chapman AB, Schwartz GL, Chai HS, Sicotte H, Kocher JP, Rodin AS, Boerwinkle E. Genomic association analysis suggests chromosome 12 locus influencing antihypertensive response to thiazide diuretic. Hypertension. 2008 Aug;52(2):359-65. PMID: 18591461

Hydralazine and *NAT2* evidence

Evidence supporting the relationship of the *NAT2* metabolic phenotype to hydralazine efficacy or PK

1. Jounela AJ, Pasanen M, Mattila MJ. Acetylator phenotype and the antihypertensive response to hydralazine. Acta Med Scand. 1975 Apr;197(4):303-6. PMID: 1136859. 40% increased hydralazine dose needed in fast acetylators for same BP effect.

2. Timbrell JA, Harland SJ, Facchini V. Polymorphic acetylation of hydralazine. Clin Pharmacol Ther. 1980 Sep;28(3):350-5. PMID: 7408395. Acetylation status relates to hydralazine metabolism.

3. Timbrell JA, Harland SJ. Identification and quantitation of hydrazine in the urine of patients treated with hydralazine. Clin Pharmacol Ther. 1979 Jul;26(1):81-8. PMID: 445966

4. Facchini V, Timbrell JA. Further evidence for an acetylator phenotype difference in the metabolism of hydralazine in man. Br J Clin Pharmacol. 1981 Apr;11(4):345-51. PMID: 7259927

5. Timbrell JA, Harland SJ, Facchini V. Effect of dose on acetylator phenotype distribution of hydralazine. Clin Pharmacol Ther. 1981 Mar;29(3):337-43. PMID: 7471604

6. Rashid JR1, Kofi-Tsepko, Juma FD. Acetylation status using hydralazine in African hypertensives at Kenyatta National Hospital. East Afr Med J. 1992 Jul;69(7):406-8. PMID: 1396201. Acetylation phenotype in African patients determines urinary MTP/hydralazine appearance.

7. Shepherd AM, Ludden TM, McNay JL, Lin MS. Hydralazine kinetics after single and repeated oral doses. Clin Pharmacol Ther. 1980 Dec;28(6):804-11. PMID: 7438695

8. Graves DA1, Muir KT, Richards W, Steiger BW, Chang I, Patel B. Hydralazine dose-response curve analysis. J Pharmacokinet Biopharm. 1990 Aug;18(4):279-91. PMID: 2231320

9. Rowell NP1, Clark K. The effects of oral hydralazine on blood pressure, cardiac output and peripheral resistance with respect to dose, age and acetylator status. Radiother Oncol. 1990 Aug;18(4):293-8. PMID: 2244017.

10. Ramsay LE, Silas JH, Ollerenshaw JD, Tucker GT, Phillips FC, Freestone S. Should the acetylator phenotype be determined when prescribing hydralazine for hypertension? Eur J Clin Pharmacol. 1984;26(1):39-42.

11. Shepherd A, Lin MS, McNay J, Ludden T, Musgrave G. Determinants of response to intravenous hydralazine in hypertension. Clin Pharmacol Ther. 1981 Dec;30(6):773-81.

12. Shepherd AM, McNay JL, Ludden TM, Lin MS, Musgrave GE. Plasma concentration and acetylator phenotype determine response to oral hydralazine. Hypertension. 1981 Sep-Oct;3(5):580-5.

13. Kalowski S1, Hua AS, Whitworth JA, Kincaid-Smith P. Hydrallazine with beta-blocker and diuretic in the treatment of hypertension. A double-blind crossover study. Med J Aust. 1979 Oct 20;2(8):439-40.

14. Zacest R, Koch-Weser J. Relation of hydralazine plasma concentration to dosage and hypotensive action. Clin Pharmacol Ther. 1972 May-Jun;13(3):420-5.

Evidence supporting the relationship of *NAT2* metabolic phenotype and genotype (could include other drugs as well)

1. Al-Ahmad MM1, Amir N1, Dhanasekaran S1, John A2, Abdulrazzaq YM3, Ali BR2, Bastaki S1. Studies on N-Acetyltransferase (NAT2) Genotype Relationships in Emiratis: Confirmation of the Existence of Phenotype Variation among Slow Acetylators. Ann Hum Genet. 2017 Sep;81(5):190-196. PMID: 28653770
2. Hein DW1, Doll MA. Accuracy of various human NAT2 SNP genotyping panels to infer rapid, intermediate and slow acetylator phenotypes. Pharmacogenomics. 2012 Jan;13(1):31-41. doi: 10.2217/pgs.11.122. Epub 2011 Nov 17. PMID: 22092036
3. Suarez-Kurtz G, Vargens DD, Sortica VA, Hutz MH. Accuracy of NAT2 SNP genotyping panels to infer acetylator phenotypes in African, Asian, Amerindian and admixed populations. Pharmacogenomics 13(8), 851–854 (2012). PMID: 22676187
4. Aklillu E1, Carrillo JA2, Makonnen E3, Bertilsson L4, Djordjevic N5. N-Acetyltransferase-2 (NAT2) phenotype is influenced by genotype-environment interaction in Ethiopians. Eur J Clin Pharmacol. 2018 Jul;74(7):903-911 PMID: 29589062
5. Others
6. Patin E1, Barreiro LB, Sabeti PC, Austerlitz F, Luca F, Sajantila A, Behar DM, Semino O, Sakuntabhai A, Guiso N, Gicquel B, McElreavey K, Harding RM, Heyer E, Quintana-Murci L. Deciphering the ancient and complex evolutionary history of human arylamine N-acetyltransferase genes. Am J Hum Genet. 2006 Mar;78(3):423-36. Epub 2006 Jan 13. PMID: 16416399

Evidence supporting the relationship of genotype to antihypertensive efficacy of hydralazine or metabolism.

1. Spinasse LB1, Santos AR, Suffys PN, Muxfeldt ES, Salles GF. PMID: 24444407. Pharmacogenomics. 2014 Feb;15(2):169-78. Different phenotypes of the NAT2 gene influences hydralazine antihypertensive response in patients with resistant hypertension. PMID: 24444407 Clinical Trial in Brazilian population assessing HTN response by NAT2 genotype
2. Garcés-Eisele SJ1, Cedillo-Carvallo B, Reyes-Núñez V, Estrada-Marín L, Vázquez-Pérez R, Juárez-Calderón M, Guzmán-García MO, Dueñas-González A, Ruiz-Argüelles A. Genetic selection of volunteers and concomitant dose adjustment leads to comparable hydralazine/valproate exposure. J Clin

Pharm Ther. 2014 Aug;39(4):368-75. PMID: 24702251. Mexican PK study showing >100% dose increase required for same AUC.

3. Allen CE N-Acetyltransferase 2 Genotype-Dependent N-Acetylation of Hydralazine in Human Hepatocytes. Drug Metab Dispos. 2017 Dec;45(12):1276-1281. PMID: 29018032

Other evidence/Gene summary:

1. McDonagh EM, Boukouvala S, Aklillu E, Hein DW, Altman RB, Klein TE. PharmGKB summary: very important pharmacogene information for N-acetyltransferase 2. Pharmacogenet Genomics. 2014 Aug;24(8):409-25. PMID: 24892773

2. BIDIL (hydralazine hydrochloride and isosorbide dinitrate) tablet, film coated. Arbor Pharmaceuticals, Inc; [Accessed 26th March 2013. .]. Drug label available from.http://dailymed.nlm.nih.gov/dailymed/lookup.cfm?setid=e1e63cd5-d1e4-4af5-bad5-1ad41ea46b00.

3. Hydralazine FDA drug label, https://www.accessdata.fda.gov/drugsatfda_docs/label/1996/008303s068lbl.pdf

4. Gonzalez-Fierro A, Vasquez-Bahena D, Taja-Chayeb L, Vidal S, Trejo-Becerril C, Pérez-Cardenas E, de la Cruz-Hernández E, Chávez-Blanco A, Gutiérrez O, Rodriguez D, Fernandez Z, Duenas-González A. Pharmacokinetics of hydralazine, an antihypertensive and DNA-demethylating agent, using controlled-release formulations designed for use in dosing schedules based on the acetylator phenotype. Int J Clin Pharmacol Ther. 2011 Aug; 49(8):519-24.

5. Candelaria M, Gallardo-Rincón D, Arce C, Cetina L, Aguilar-Ponce JL, Arrieta O, González-Fierro A, Chávez-Blanco A, de la Cruz-Hernández E, Camargo MF, Trejo-Becerril C, Pérez-Cárdenas E, Pérez-Plasencia C, Taja-Chayeb L, Wegman-Ostrosky T, Revilla-Vazquez A, Dueñas-González A. A phase II study of epigenetic therapy with hydralazine and magnesium valproate to overcome chemotherapy resistance in refractory solid tumors. Ann Oncol. 2007 Sep; 18(9):1529-38.

Negative studies of Hydralazine:

6. M J Vandenburg, P Wright, J Holmes, H J Rogers, and R A Ahmad. The hypotensive response to hydralazine, in triple therapy, is not related to acetylator phenotype. Br J Clin Pharmacol. 1982 May; 13(5): 747–750.

7. Hunyor SN. Hydrallazine and beta-blockade in refractory hypertension with characterization of acetylator phenotype. Aust N Z J Med. 1975 Dec;5(6):530-6.

8. Ludden TM, Shepherd AM, McNay JL Jr, Lin MS. Effect of intravenous dose on hydralazine kinetics after administration. Clin Pharmacol Ther. 1983 Aug;34(2):148-52.

Final Statistical Analysis Plan

Genetic Testing to Understand and Address Renal Disease Disparities Across the United States (GUARDD-US)

(For Main Study Primary Manuscript)

Study Funding:	National Human Genome Research Institute
Protocol Version:	6.0
Protocol Date:	19 March 2024
Analysis Plan Version:	1.1
Analysis Plan Date:	July 08, 2024

Revision History

Version	Date	File Name	Brief Summary of Changes
V1.0	11AUG2023	GUARDD-US_Main_Study_Final_Analysis_SAP_v1.0	Original
V1.1	08JUL2024	GUARDD-US_Main_Study_Final_Analysis_SAP_v1.1	• Made some grammatic corrections and minor wording adjustments across the SAP<br>• Change the term “sensitivity analysis" to “additional analysis” for some analyses proposed (sections 4.3, 4.4 and 7.2)<br>• Added clarifications to General Analysis Conventions (section 5)<br>• Added definition for baseline diabetes (section 6.2)<br>• Added descriptive analyses for study implementation characteristics (section 6.3)<br>• Updated the analysis method for the Per-protocol analysis and the As-treated analysis (sections 7.2.2 and 7.2.3)<br>• Removed CPT code 82570 because it is not a urine proteinuria or urine microalbuminuria diagnostic code (section 7.3.4.1)<br>• Updated the definitions for Controlled BP and Uncontrolled BP (section 7.4.3)<br>• Provided more details on existing subgroup analyses (sections 7.4.3 and 7.4.4)<br>• The analysis comparing treatment effects between participants with and without hypertension in the subset with SBP above 140 mmHg is removed (section 7.4.3)<br>• Specified additional subgroup analyses (section 7.4.5)

Version	Date	File Name	Brief Summary of Changes
			• Added interaction analysis on APOL1 by Treatment (section 7.4.6)<br>• Added analyses on other continuous SBP and DBP outcomes (section 7.4.7)<br>• Added analyses on BP control (yes/no) outcome (section 7.4.8)<br>• Added analyses on psycho-behavioral outcomes (section 7.4.9)<br>• Added a section on potential post-hoc analyses (section 7.4.10)

Signature page

Prepared by:

Lilin She
Biostatistician, Senior
Duke Clinical Research Institute

Signature:___________________________ Date: ______________

Approved by:

Hrishikesh Chakraborty, DrPH
Co-Principal Investigator, DCC
Professor of Biostatistics and Bioinformatics
Duke Clinical Research Institute

Signature:___________________________ Date: ______________

Carol R. Horowitz, MD, MPH
Principal Investigator
Professor of Medicine
Icahn School of Medicine at Mount Sinai

Signature:___________________________ Date: ______________

Girish Nadkarni, MD
Co-Investigator
Professor of Medicine
Icahn School of Medicine at Mount Sinai

Signature:___________________________ Date: ______________

Christina Wyatt, MD, MSc
Co-Principal Investigator
Associate Professor of Medicine
Division of Nephrology at Duke University School of Medicine
Duke Clinical Research Institute

Signature:___________________________ Date: ______________

Michael T. Eadon, MD
Co-Investigator
Associate Professor of Medicine
Indiana University School of Medicine

Signature:__________________________ Date: _____________

Kerri L. Cavanaugh, MD, MHS
Co-Investigator
Associate Professor of Medicine
Division of Nephrology and Hypertension
Vanderbilt University Medical Center.

Signature:__________________________ Date: _____________

Rhonda DeHoff, PharmD, MS, FAHA, FACC, FACCP
Co-Investigator
Associate Professor of Pharmacotherapy & Translational Research
Colleges of Pharmacy and Medicine
University of Florida

Signature:__________________________ Date: _____________

TABLE OF CONTENTS

1 Introduction 9
1.1 Study Design 9
1.2 Sample Size and Power 9
2 Objectives of Study 10
2.1 Primary 10
2.2 Secondary 10
2.3 Pharmacogenetic (PGx) Substudy 10
3 Data Sources 10
4 Analysis populations 11
4.1 Intention-to-treat (ITT) Population 11
4.2 Modified Intention-to-treat (mITT) population 11
4.3 Per-protocol (PP) Population 11
4.4 As-treated (AT) Population 11
4.5 PGx Substudy Population 12
5 General Analysis Conventions 12
5.1 General Principles 12
5.2 Multiple Comparisons 12
5.3 Blinding 13
5.4 Timing of Analyses 13
6 Participant and Study Implementation Characteristics 13
6.1 Disposition of Participants 13
6.2 Baseline Characteristics 14
6.3 Study Implementation Characteristics 14
7 Efficacy Evaluation 15
7.1 Primary Endpoint and Analysis 15
7.1.1 Primary Endpoint 15
7.1.2 Primary Hypotheses 16
7.1.3 Primary Analysis 16
7.2 Additional Analyses on Primary Endpoint 16
7.2.1 Covariate-adjusted Analysis 16

7.2.2 Per-protocol Analysis 17
7.2.3 As-Treated Analysis 17
7.3 Secondary Endpoints and Analyses 17
7.3.1 Secondary Endpoints 17
7.3.2 Hypotheses for Secondary Endpoint Analyses 17
7.3.3 Secondary Endpoint Analyses 17
7.3.4 Secondary Endpoint Data Element Definitions 18
7.4 Exploratory Analyses 19
7.4.1 Trends in SBP 19
7.4.2 CKD Subsets 19
7.4.3 Controlled versus Uncontrolled Blood Pressure Subsets 20
7.4.4 Change in Documented CKD Diagnosis among Participants with and without CKD (Disease) at Baseline 20
7.4.5 Additional Subgroup Analyses on Primary Endpoint 20
7.4.6 Interaction of APOL1 Phenotype and Randomized Treatment 24
7.4.7 Analyses on Other Continuous Blood Pressure Outcomes 24
7.4.8 Blood Pressure Control Outcome 25
7.4.9 Psycho-Behavioral Outcomes of Participants 25
7.4.10 Potential Post-hoc Analyses 26
7.5 Handling of Missing Data 26
8 Safety Evaluation 27
9 References 27

List of Abbreviations

Abbreviation	Definition
AT	As-treated
BP	Blood Pressure
CKD	Chronic Kidney Disease
DBP	Diastolic Blood Pressure
DCC	Data Coordinating Center
DCRI	Duke Clinical Research Institute
DSMB	Data and Safety Monitoring Board
eCRF	Electronic Case Report Form
EHR	Electronic Health Record
HTN	Hypertension
IGNITE	Implementing Genomics in Practice
ITT	Intention-To-Treat
mITT	Modified Intention-to-Treat
MMRM	Mixed Model for Repeated Measures
NHGRI	National Human Genome Research Institute
PGx	Pharmacogenetic
PP	Per-protocol
ROR	Return Of Results
SAP	Statistical Analysis Plan
SBP	Systolic Blood Pressure
Y/N	Yes/No

1 Introduction

GUARDD-US is a prospective, multicenter, unblinded, two arm randomized pragmatic clinical trial, and is one of the clinical trials conducted by the Implementing Genomics in Practice (IGNITE): Pragmatic Trials Network. The main objective of GUARDD-US is to determine the effect of participant and provider knowledge of a high-risk Apolipoprotein L1 (*APOL1*) genotype ("positive *APOL1* status", defined by the presence of two risk alleles) on blood pressure, urine testing to detect and monitor chronic kidney disease (CKD), and appropriate diagnosis of CKD.

All participants enrolled in this study are adults of self-reported African ancestry with hypertension that either: 1) do not have diabetes and do not have CKD, or 2) have CKD (with or without diabetes).

GUARDD-US includes a pharmacogenetic (PGx) substudy to determine the effect of returning PGx test results that predict efficacy of specific antihypertensive medications on change in SBP from baseline to 3 months in *APOL1* negative individuals (i.e., 0/1 *APOL1* CKD risk alleles).

1.1 Study Design

Participants are randomized in a 1:1 ratio to immediate *APOL1* genotype testing and return of results (ROR) to participant and provider (Intervention arm) versus delayed *APOL1* genotype testing and ROR to participant and provider (Control arm). The primary analysis will compare SBP change from baseline to 3 months between *APOL1* positive participants in the Intervention arm (i.e., early knowledge of risk status) to *APOL1* positive participants in the Control arm (i.e., delayed knowledge of *APOL1* risk status). Participants that are *APOL1* negative will not be included in the main study analyses for primary manuscript but will be included in other analyses of survey and clinical data. These other analyses will not be covered in this SAP.

Participants who are randomized to immediate *APOL1* genotype testing and return of results (ROR) to participant and provider (Intervention arm) will simultaneously be randomized to the PGx substudy intervention (i.e., immediate PGx gene testing and ROR to participant and provider) or the PGx control (delayed PGx gene testing and ROR to participant and provider) using a 1:1 allocation ratio. Among the sites that participate in the substudy, participants that test negative for *APOL1* will proceed into the PGx substudy under their assigned PGx treatment arms. Analyses for substudy data will not be covered in this SAP.

Participants enrolled in the study are scheduled to have baseline, 3-month, and 6-month study visits.

1.2 Sample Size and Power

GUARDD-US main study will randomize approximately 6700 individuals, including approximately 3350 individuals in each arm. Assuming a 14% prevalence of *APOL1* positive status, a sample size of 6700 for the randomized population would result in 938 *APOL1* positive individuals with 469 in each arm for the main study primary analysis population.

In order to maintain an overall alpha of 0.05 and account for two interim analyses, we will use a two-sided alpha of 0.0492 for the final analysis of the primary endpoint for the main study. For a two-tailed t-test, this leads to an estimated sample size of 938 *APOL1* positive participants (i.e., approximately 6700 randomized participants with 14% of *APOL1* positive rate). This sample size will provide 80% power to detect a 3.5 mmHg difference in SBP change from baseline to 3 months assuming a standard deviation of 18.1 mmHg for the SBP change and a 10% lost to follow-up rate.

Of the approximately 6700 participants in the GUARDD-US study, approximately 4400 will also participate in the PGx substudy based on enrollment at a participating PGx substudy site. A sample size of 4400 with a 14% prevalence of *APOL1* positive status would result in 1892 *APOL1* negative participants in the Intervention arm, who represent the study population for the PGx substudy. This would include approximately 946 participants in the PGx Control (delayed return of PGx results) and PGx Intervention arms (early return of PGx results).

2 Objectives of Study

2.1 Primary

The primary aim is to determine the effect of participant and provider knowledge of a positive *APOL1* status and accompanying guideline based clinical decision support on change in systolic blood pressure (SBP) from baseline to 3 months after randomization among *APOL1* positive participants.

2.2 Secondary

1. Determine the effect of participant and provider knowledge of a positive *APOL1* status on the probability of appropriate CKD diagnosis.
2. Determine the effect of participant and provider knowledge of a positive *APOL1* status on the probability of receiving a urine test for CKD.
3. Explore cost effectiveness, mediators, moderators, psycho-behavioral impact of results disclosure on participants, and effects of knowledge of *APOL1* status on provider intervention recommendations.

2.3 Pharmacogenetic (PGx) Substudy

The PGx substudy is nested within the overall GUARDD study. *APOL1* negative individuals in the Intervention arm at participating sites will be included in the PGx substudy. This substudy will investigate the effect of knowledge of genetic test results that predict efficacy of various antihypertensive medications on initial or add-on antihypertensive therapy management in *APOL1* negative individuals.

This SAP contains details on the statistical methods for the analyses and summaries of the main study data that are to be performed at the end of this study to help support the publication of primary manuscript for this trial. Details regarding analyses of the PGx substudy and Cost-Effectiveness (CE) can be found in separate SAPs. Information on table, figure, and listing shells to support the SAP will be in a supplemental Appendix.

3 Data Sources

The primary source of data for the GUARDD-US study will be the study database (REDCap) which will collect consent, safety events and site-reported protocol deviation/violation, baseline and follow-up characteristics including the primary endpoint (i.e., SBP), and data collected in baseline, 3- and 6-month patient surveys. For the secondary endpoints, sites will be given a list of information to extract from the electronic health record for their GUARDD-US participants, a common format that data should be converted to, and procedures for secure file transfer to the Coordinating Center. Transfers of data will occur after approximately half of the participants have completed their 6-month visit and 12 months after the last participant is randomized.

Database will be locked before the statistical analyses are finalized.

4 Analysis populations

4.1 Intention-to-treat (ITT) Population

This population includes all participants in this study that are randomized to Intervention arm (i.e., immediate *APOL1* gene testing and ROR to participant and provider) and Control arm (delayed *APOL1* gene testing and ROR to participant and provider). In this study, ITT population is the same as the randomized population.

4.2 Modified Intention-to-treat (mITT) population

This population includes all participants who are randomized and who test positive for *APOL1* high-risk genotype. The mITT population is a subset of ITT population. Unless otherwise specified, mITT population will be used in the GUARDD-US main study primary and secondary endpoint analyses. Exploratory analyses will also be performed using mITT population, unless otherwise specified. All analyses using mITT population will be performed by randomized treatment.

4.3 Per-protocol (PP) Population

The per-protocol population includes mITT participants who did not have a known protocol deviation or violation and have complete SBP data at baseline and 3 months. Protocol deviations or violations include:

- Protocol deviation or violation event(s) reported in eCRF Events page.
- Treatment crossovers as identified by actual ROR time reported in eCRF not consistent with the assigned ROR time per randomized treatment.

Treatment crossover refers to a protocol deviation that involves a participant randomized in one treatment arm actually receives treatment for another arm. This type of treatment switch occurs during the course of the trial. Specifically, it refers to the treatment deviation related the timing of the return of the genetic testing results:

- Participants randomized to the delayed arm who received their genetic testing result prior to the primary endpoint time-point (i.e. 3 months) OR
- Participants randomized to the immediate arm who receive their genetic testing result after the primary endpoint time-point (i.e. 3 months)

The primary analysis will be repeated in the per-protocol (PP) population as an additional analysis.

We plan to exclude all participants who have a known protocol deviation or violation from the analyses using "Per-protocol Population". Participants who have missing data on ROR date will also be excluded from this PP population.

4.4 As-treated (AT) Population

If more than 5% of the participants in mITT population have treatment crossover, an additional analysis will be performed using the "As-treated" (AT) population. Different from per-protocol population, participants with treatment crossover will be analyzed according to their actual treatment received in the as-treated population.

- Participants randomized to the delayed arm who received their genetic testing result prior to the primary endpoint time-point (i.e. 3 months) will be analyzed as participants in the immediate arm.

- Participants randomized to the immediate arm who receive their genetic testing result after the primary endpoint time-point (i.e. 3 months) will be analyzed as participants in the delayed arm.

Participants who have treatment crossover will be included in the as-treated population. The only participants that will be excluded from the as-treated population are those who have missing data on ROR date.

4.5 PGx Substudy Population

The population for the PGx Substudy is defined as participants who are enrolled at sites participating in the PGx substudy and randomized to the Intervention arm and who test negative for *APOL1* CKD high-risk genotype. PGx substudy population will only be used in the PGx substudy analyses that will be covered in a separate SAP for the PGx substudy.

5 General Analysis Conventions

5.1 General Principles

Unless otherwise specified, participants in modified intention-to-treat (mITT) population from all study sites will be combined and included in the primary, secondary and exploratory analyses by randomized treatment groups [i.e., immediate *APOL1* genotype testing and return of results (ROR) to participant and provider (Intervention arm) versus delayed *APOL1* genotype testing and ROR to participant and provider (Control arm)] regardless of their actual treatment received. Analyses based on per-protocol population will also be performed by randomized treatment, with participants who have treatment crossover, other known protocol deviations/violations or incomplete SBP data excluded. The analysis using as-treated population will be based on actual treatment received.

Unless otherwise specified, descriptive statistics in this study will be presented as mean, standard deviation, median, percentiles (25th and 75th), and range as appropriate for the continuous variables, as frequencies and percentages for the categorical variables.

Unless otherwise specified, summaries of continuous characteristics will be based on non-missing observations. The percentage for categorical variables will be calculated based on the number of participants with non-missing values for the variable.

Unless otherwise specified, statistical comparisons will be performed using two-sided significance tests. To maintain an overall alpha of 0.05 and account for two interim analyses, we will use a two-sided alpha of 0.0492 for the primary analysis. For all the other analyses, p-values will be reported along with the corresponding estimates of treatment effect and 95% confidence intervals. Additional details of specific analyses are presented in the sections below.

5.2 Multiple Comparisons

With the primary and various secondary endpoints that have been outlined in the protocol, there is a multiplicity of analyses to be performed, which leads to an increased probability that at least one of the comparisons could be "significant" by chance. Adjusting for the effects of the repeated significance testing for the multiplicity of secondary endpoints would require that very small significance levels be used for every comparison. Therefore,

rather than adjusting for multiple comparisons, we will be conservative in the interpretation of secondary and exploratory analyses.

5.3 Blinding

The randomization scheme was created by a statistician at DCRI, who operates independently and is segregated from the trial's operational team to ensure integrity. The operational team, comprising study investigators, their staff, and operational statisticians, does not have access to the randomization scheme, thereby preserving blinding to the aggregated data by treatment allocation. This operational team oversees the daily management of the trial and develops the statistical analysis plan, all while maintaining their blinding. At the time of database lock, the unblinded treatment codes will be combined with the blinded data reports containing aggregated data.

5.4 Timing of Analyses

All the analyses with unblinded treatment codes will be performed at the end of the study, meaning after the final participant has completed their last follow-up visit or contact, and the database is properly cleaned and locked. These analyses will be finalized and validated in adherence to DCRI Statistic Group Standard Operation Procedures (SOPs) before the unblinded final analysis results and the unblinded analysis datasets are shared beyond DCRI Statistic Group.

All statistical analyses will be performed using SAS statistical software Version 9.4 or higher (SAS Institute Inc., Cary, NC).

6 Participant and Study Implementation Characteristics

6.1 Disposition of Participants

Disposition data for all participants (number of participants who were screened, randomized, completed genotyping, received ROR, withdrew consent, lost to follow-up, died prior to 3-month visits, etc.) will be summarized by randomized treatment groups for ITT and mITT populations. The summary will include:

- Number of participants who were screened, randomized, completed genotyping, and/or received ROR,
- Inclusion in ITT and mITT populations
- Participants completing 3-month and/or 6-month visits
- Lost to follow-up
- Withdrew consent for follow-up
- Died prior to 3 months or 6 months

6.2 Baseline Characteristics

The baseline demographic, socioeconomic and clinical characteristics will be tabulated and summarized as descriptive statistics for both ITT and mITT populations by randomized treatment group. The data will be presented and compared by randomized treatment groups. Statistical tests will be performed using Wilcoxon rank-sum test for continuous variables and Chi-square test or Fisher's Exact test (if any of the expected cell count is less than 5) for categorical variables. The corresponding p-values will be presented for the comparison between treatment groups.

Baseline CKD is a binary variable (Y/N) where 'Y' is defined as [the presence of a CKD ICD10 diagnosis code (i.e., N18.x; E08.22; E09.22; E10.22; E11.22; E13.22) in the 24 months prior to baseline)] OR [having 2 lab results of eGFR ≤ 60 mL/min/1.73m2 3 months apart within the 24 months prior to baseline] OR [having 2 lab results of microalbuminuria or urine albumin-to-creatinine ratio (UACR) level >30 mg/g or equivalent 3 months apart within the 24 months prior to baseline] OR [urine protein creatinine ratio (UPCR) > 200 mg/g 3 months apart within the 24 months prior to baseline].

Baseline CKD will be used in the summary of baseline characteristics, the CKD subgroup analysis, and the baseline covariate adjusted analyses.

All eGFR data are obtained from creatinine data in EHR after converting from standardized serum creatinine (mg/dL) to eGFR (mL/min/1.73m2) using the CKD-EPI creatinine equation (2021) below:

$$\mathbf{eGFR_{cr} = 142 \times \min(S_{cr}/\kappa, 1)^{\alpha} \times \max(S_{cr}/\kappa, 1)^{-1.200} \times 0.9938^{Age} \times 1.012\ [if\ female]}$$

where:
S_{cr} = standardized serum creatinine in mg/dL
κ = 0.7 (females) or 0.9 (males)
α = -0.241 (female) or -0.302 (male)
$\min(S_{cr}/\kappa, 1)$ is the minimum of S_{cr}/κ or 1.0
$\max(S_{cr}/\kappa, 1)$ is the maximum of S_{cr}/κ or 1.0
Age (years)

Diabetes is defined by EHR data within 12 months prior to randomization:

- HbA1c ≥ 6.5% at least one time within 12 months prior to randomization date OR
- ICD10 diagnosis codes (see the GUARDD-US study protocol Appendix A) at least one time within 12 months prior to randomization date OR
- Having diabetes in the participant's medical record problem list at least one time within 12 months prior to randomization date.

6.3 Study Implementation Characteristics

Descriptive statistics will be utilized to summarize data related to the implementation of APOL1 testing and the return of results (ROR). Additionally, data on the completeness of the primary endpoint measurement will be included in these summaries. The summary will include:

- Number of ITT population participants with an APOL1 test result imported to the study database.

- The baseline characteristics for ITT population participants with and without APOL1 test result will be compared. The same comparison will be performed for ITT population participants who have APOL1 positive and APOL1 negative results.

- The duration from sample collection to APOL1 return of results (ROR) to participants (i.e., days from sample collection to ROR). This data summary will also be presented for both ITT and mITT populations by treatment groups and by the five clinical groups that participated in the study, enabling the assessment of site heterogeneity.

- APOL1 test result exposure time prior to the primary endpoint assessment time (i.e., days from ROR to the time of 3-month SBP measurement) will be compared for participants in two treatment groups of the mITT population.

- Number of participants with APOL1 results returned appropriately (i.e., participants who do not have treatment crossover) in mITT population.

- Number of participants who have non-missing SBP results at baseline, 3-month, and/or 6-month in mITT population.

- The baseline characteristics for participants with and without missing data on primary endpoint (i.e., Change in SBP from baseline to 3 months) will be compared among participants in mITT population.

- Number of participants who have early discontinuation from the study (i.e., participants who discontinued from the study prior to the SBP assessment at 3 months after randomization). The baseline characteristics of participants will be compared between two treatment groups for participants with and without study early discontinuation. This comparison will be performed in mITT population.

- Number of participants with SBP results obtained from in-person measurements, from EHR, or from either of them for both ITT and mITT populations.

- Data on blood pressure (BP) medication prescription reported in the study database Medication Inventory form at baseline, 3-month and 6-month will be summarized by randomized treatment for mITT population.

- Data on medication adherence at baseline, 3-month and 6-month. Medication adherence will be derived from survey data that is based on Voils Domains Of Subjective Extent of Nonadherence (DOSE-Nonadherence) measure.

7 Efficacy Evaluation

7.1 Primary Endpoint and Analysis

7.1.1 Primary Endpoint

The primary endpoint is the change in systolic blood pressure (SBP) from baseline to the 3-month study visit. Baseline SBP will be the average of the 2nd and 3rd of the three SBP measures performed at the baseline study visit prior to randomization. Similarly, 3-month SBP will be the average of the 2nd and 3rd SBP measures at the 3-month visit, or alternatively the SBP from the EHR entered the study database by the site in accordance with the study manual of procedures (MOP). If either the baseline or 3-month SBP is missing, then the change of SBP will be missing.

7.1.2 Primary Hypotheses

H0 (null hypothesis): Providing participant and provider knowledge of a positive *APOL1* status has no effect on the change in SBP from baseline to 3 months.

H1 (alternative hypothesis): Providing participant and provider knowledge of a positive *APOL1* status does affect the change in SBP from baseline to 3 months.

7.1.3 Primary Analysis

A two-sided t-test will be used to evaluate the difference in the change in SBP from baseline to the 3-month study visit between two randomized treatment groups (i.e., Immediate *APOL1* genotype testing & ROR and Delayed *APOL1* testing & ROR) among participants from mITT population.

To maintain the overall significance level of 0.05, the final analysis of the primary endpoint will be considered significant for p-value less than 0.0492 (adjusted for two interim analyses).

Summary statistics will be provided for the SBP at baseline and 3 months for each treatment arm. The change in SBP from baseline to 3 months will also be presented. P-value to test the difference in the SBP change from baseline to 3 months between two treatment groups will be provided using two-sided t-test.

7.2 Additional Analyses on Primary Endpoint

7.2.1 Covariate-adjusted Analysis

For the primary endpoint (i.e., SBP change from baseline to 3 months), we will conduct covariate-adjusted linear regression analysis to account for differences in baseline characteristics between the Immediate *APOL1* Testing & ROR Intervention and Delayed *APOL1* Testing & ROR. This analysis will be performed using mITT population. No SBP data for 6 months will be included in this analysis.

The model will include randomized treatment as an explanatory factor and baseline SBP as a covariate. Other baseline covariates are pre-specified according to whether they are prognostic for the primary endpoint in this trial (i.e., SBP change from baseline to 3 months). The term prognostic baseline covariates used here refers to the covariates that are likely associated with the primary endpoint. Pre-specified baseline covariates include CKD, age, and sex.

If any of the pre-specified baseline covariates has ≥ 1% of participants with missing data, the covariates will not be used in the analyses to maintain the mITT population for the model. If the baseline covariate has missing data on < 1% of the participants, we will still use the covariate in the model and let participants with missing value on the covariate be excluded from the model.

Collinearity among the baseline covariates will be assessed by pairwise correlations. Covariates with <0.6 correlation coefficient will be included in the model.

If the model fails to converge or encounter difficulties, we will attempt to simplify the model to eliminate the problems.

Least-square mean estimates and 95% CI for SBP change for each treatment group and the difference between two groups will be reported. The p-value for the difference in the by-treatment group least square mean estimates derived from the model will be presented.

7.2.2 Per-protocol Analysis

An additional analysis for the primary endpoint will be applied to the per-protocol population using covariate-adjusted linear regression model. In addition to the covariates pre-specified for the primary endpoint analysis in Section 7.2.1, we will also adjust for the site variable. This additional adjustment accounts for the possible site heterogeneity in the turn-around time in APOL1 return of results (ROR) among the five participating clinical groups. Only participants with non-missing data on SBP change from baseline to 3 months are included in this additional analysis.

7.2.3 As-Treated Analysis

An additional analysis for the primary endpoint will be performed using the as-treatment (AT) population, if >5% of mITT participants have treatment crossover. Covariate-adjusted linear regression model will be used in this analysis. Only participants with non-missing data on SBP change from baseline to 3 months are included in this additional analysis. In addition to the covariates pre-specified for the primary endpoint analysis in Section 7.2.1, we will also adjust for the site variable. This additional adjustment accounts for the possible site heterogeneity in the turn-around time in APOL1 return of results (ROR) among the five participating clinical groups.

7.3 Secondary Endpoints and Analyses

7.3.1 Secondary Endpoints

The secondary endpoints for the GUARDD-US Study are as follows:

- Change in urine microalbuminuria/proteinuria orders from baseline to 6 months
- Documented order of microalbuminuria/proteinuria tests by 6 months
- Change in documented diagnosis for stage 3 CKD and above from baseline to 6 months
- Documented diagnosis of CKD stage 3 and above by 6 months
- Change in documented diagnosis for any stage CKD from baseline to 6 months
- Documented diagnosis of all stages of CKD by 6 months

7.3.2 Hypotheses for Secondary Endpoint Analyses

H0 (null hypothesis): Providing participant and provider knowledge of a positive *APOL1* status has no effect on the probability of documented CKD diagnosis or on the probability of receiving a urine microalbuminuria/proteinuria testing.

H1 (alternative hypothesis): Providing participant and provider knowledge of a positive *APOL1* status does affect on the probability of documented CKD diagnosis or on the probability of receiving a urine microalbuminuria/proteinuria testing.

7.3.3 Secondary Endpoint Analyses

Secondary endpoints are all binary yes/no variables. Analysis of all the secondary endpoints will use a difference in proportions test. Additionally, counts of each of the secondary endpoints and their baseline and 3-month components by randomized treatment arm will be reported. The difference between two proportions, its 95% confidence interval, and p-value will be reported.

7.3.4 Secondary Endpoint Data Element Definitions

7.3.4.1 Change in Urine Microalbuminuria/Proteinuria Orders from Baseline to 6 Months (Y/N)

a) Order of urine microalbuminuria/proteinuria test at baseline is a binary variable (Y/N) where 'Y' is defined as the presence of urine proteinuria or urine microalbuminuria diagnostic code (i.e. 82043/84156 (Current Procedural Terminology (CPT) codes) OR the presence of urine microalbuminuria/proteinuria lab test order and/or result in the participant's EHR record at any point in the 12 months prior to baseline.

b) Order of urine microalbuminuria/proteinuria test at 6 months is a binary variable (Y/N) where 'Y' is defined as the presence of urine proteinuria or urine microalbuminuria diagnostic code 82043/84156 (CPT codes) OR the presence of urine microalbuminuria/proteinuria lab test order and/or result in the participant's EHR record at any point from baseline to 6 months.

c) Change in urine microalbuminuria/proteinuria orders is a binary variable (Y/N) where 'Y' is defined as no order of urine microalbuminuria/proteinuria test at baseline and having an order of urine microalbuminuria/proteinuria test at 6 months [No for a, Yes for b = NY].

7.3.4.2 Documented Order of Microalbuminuria/Proteinuria Tests by 6 Months

Documented order of microalbuminuria/proteinuria tests by 6 months is a binary variable (Y/N) where 'Y' is defined as the change in urine microalbuminuria/proteinuria orders (as described in 7.3.4.1 c) OR (order of urine microalbuminuria/proteinuria test at baseline) [NY, YN or YY].

7.3.4.3 Change in Documented Diagnosis for Stage 3 CKD and above from Baseline to 6 Months

a) Documented diagnosis at baseline is a binary variable (Y/N) where 'Y' is defined as: [the presence of a CKD ICD10 diagnosis codes of N18.3 – N18.5 in the 24 months prior to baseline]

b) Documented diagnosis at 6 months is a binary variable (Y/N) where 'Y' is defined as [the presence of a CKD ICD10 diagnosis codes of N18.3 – N18.6, in the 6 months after baseline]

c) Change in documented diagnosis for stage 3 CKD and above is a binary variable (Y/N) where 'Y' is defined as no documented diagnosis at baseline and documented diagnosis at 6 months. [No for a, Yes for b = NY].

7.3.4.4 Documented Diagnosis of CKD Stage 3 and above by 6 Months

Documented diagnosis of stage 3 and above CKD by 6 months is a binary variable (Y/N) where 'Y' is defined as (change in documented diagnosis for stage 3 or above CKD (as described in 7.3.4.3c)) OR (documented diagnosis at baseline (as described in 7.3.4.3a)) [NY, YN or YY]

7.3.4.5 Change in Documented Diagnosis for Any Stage CKD from Baseline to 6 Months

a) Documented diagnosis at baseline is a binary variable (Y/N) where 'Y' is defined as [the presence of a CKD ICD10 diagnosis code (i.e., N18.x; E08.22; E09.22; E10.22; E11.22; E13.22) in the 24 months prior to baseline].

b) Documented diagnosis at 6 months is a binary variable (Y/N) where 'Y' is defined as [the presence of a CKD ICD10 diagnosis code (i.e., N18.x; E08.22; E09.22; E10.22; E11.22; E13.22) in the 6 months after baseline].

c) Change in documented diagnosis for any stage CKD is a binary variable defined as no documented diagnosis at baseline and documented diagnosis at 6 months [No for a, Yes for b = NY].

7.3.4.6 Documented Diagnosis of Any Stage CKD by 6 Months (Y/N)

Documented diagnosis of any stage CKD by 6 months is a binary variable (Y/N) where 'Y' is defined as (the change in documented diagnosis of any stage CKD (as described in 7.3.4.5c)) OR (documented diagnosis at baseline (as described in 7.3.4.5a)) [NY, YN or YY]

7.4 Exploratory Analyses

For analyses using covariate-adjusted models, potential baseline covariates included will be the same as those pre-specified for the covariate-adjusted analysis for primary endpoint.

For all analyses using mixed model for repeated measures, appropriate covariance matrix (e.g., compound symmetry, autoregressive, variance components, or other covariance structure) will be selected based on the data to account for the correlated SBP measurements from individual participants. We will select the covariance structure that has the smallest Akaike's Information Criteria (AICC) value. When two or more covariance structures yield a similarly small AICC value, the most parsimonious model with the fewest number of parameters (i.e., distinct covariance values) will be selected.

7.4.1 Trends in SBP

We will compare the time trends in SBP from baseline to each post-baseline time point (including 3 months and 6 months) between two randomized treatment groups (i.e., Immediate *APOL1* Testing & ROR and Delayed *APOL1* Testing & ROR) using a repeated measures mixed model. More specifically, we will conduct covariate-adjusted repeated measures mixed models that will account for differences in baseline characteristics that differ between the Immediate *APOL1* Testing & ROR and Delayed *APOL1* Testing & ROR.

7.4.2 CKD Subsets

If the data provide evidence of an overall difference in the primary outcome (change in SBP from baseline to 3 months) between Immediate *APOL1* Testing & ROR and Delayed *APOL1* Testing & ROR, we will further examine whether the effect of knowledge of a positive *APOL1* status on SBP differs among those with and without CKD. This subgroup analysis will involve testing for interactions between intervention arm and CKD using a covariate-adjusted linear regression model. Effect estimates will be conservatively interpreted in conjunction with the formal interaction test. If the interaction is significant, we will test whether the time trends in effect of early

versus delayed knowledge of a positive *APOL1* status on SBP differs among Immediate *APOL1* Testing & ROR and Delayed *APOL1* Testing & ROR in those with CKD at baseline.

7.4.3 Controlled versus Uncontrolled Blood Pressure Subsets

We will also examine whether the effect of knowledge of a positive *APOL1* status on SBP differs among those whose blood pressure is well-controlled at baseline (defined as SBP < 140 mmHg and DBP < 90 mmHg) versus those whose blood pressure is not well-controlled at baseline (i.e., SBP ≥ 140 mmHg or DBP ≥ 90 mmHg). This subgroup analysis will involve testing for interactions between intervention arm and blood pressure control at baseline using a covariate-adjusted linear regression model. Effect estimates will be conservatively interpreted in conjunction with the formal interaction test.

7.4.4 Change in Documented CKD Diagnosis among Participants with and without CKD (Disease) at Baseline

In this subgroup analysis, our objective is to explore whether knowledge of a positive *APOL1* status affects the changes in documented chronic kidney disease (CKD) diagnosis differently among participants with and without CKD at baseline. We will assess this by testing the interaction between the study intervention and the baseline CKD (disease) subgroup factor using a covariate-adjusted logistic regression model. The same baseline covariates specified for the primary endpoint model will be adjusted in this analysis. We will compare the treatment effect on the change (baseline to 6 months) in documented CKD diagnosis (ICD code) among participants with and without CKD (disease) at baseline. The treatment effect within each baseline CKD subgroup will be evaluated by odds ratio and its 95% CI. The interaction p-value will be reported.

a) Documented CKD diagnosis at baseline is a binary variable (Y/N) where 'Y' is defined as [the presence of a CKD ICD10 diagnosis code (i.e., N18.x; E08.22; E09.22; E10.22; E11.22; E13.22) in the 24 months prior to baseline)].

b) Documented CKD diagnosis at 6 months is a binary variable (Y/N) where 'Y' is defined as [the presence of a CKD ICD10 diagnosis code (i.e., N18.x; E08.22; E09.22; E10.22; E11.22; E13.22) in the 6 months after baseline]

c) Change in documented CKD diagnosis for any stage CKD is a binary variable defined as no documented CKD diagnosis at baseline and with documented CKD diagnosis at 6 months. [No for a, Yes for b = NY].

7.4.5 Additional Subgroup Analyses on Primary Endpoint

All subgroup analyses will be performed for the primary endpoint in the mITT population to explore whether treatment effects on blood pressure are consistent across subgroups. Subgroup analyses will be performed using the same linear regression model as that for the covariate-adjusted analysis for the primary endpoint, with the addition of the subgroup factor and its interaction with the randomized treatment.

The subgroup analysis results will be summarized via a forest plot and interaction p-values will be reported. We will look further into the randomized treatment effect within a subgroup if any interaction p-value is smaller than 0.05.

The estimated treatment effect along with its 95% confidence interval within each subgroup will be reported on the forest plot(s).

If any of the subgroup categories contains less than 10% of the participants in the mITT population, the analysis based on that subgroup factor will not be conducted. This approach helps to avoid having subgroups with very small sample sizes.

Three subgroup analyses on the primary endpoint have already been pre-specified in Sections 7.4.2 and 7.4.3:

- **With vs. without CKD at baseline**
- **Uncontrolled BP vs. controlled BP control at baseline**
- **(Uncontrolled BP + HTN Intervention) vs. Other**

Additional pre-specified subgroup factors are provided below. The key subgroups of interest will be categorized based on these baseline factors:

- **Age (< median vs. ≥ median)**
- **Sex (Male vs. Female)**
- **eGFR (≤ 60 mL/min/1.73m2 vs. Other)**
    - eGFR data is obtained from EHR. Please see Section 6.2 on how eGFR should be computed.
    - Each participant could have multiple eGFR laboratory measures within 24 months prior to baseline. “eGFR ≤ 60 mL/min/1.73m2” is defined by a participant having two laboratory results of eGFR ≤ 60 mL/min/1.73m2 that are at least 3 months apart within the 24 months prior to baseline.
    - For participants whose baseline eGFR data are unavailable in the EHR, if they have ICD-10 codes corresponding to N18.3 or N18.4 in their baseline EHR data, they will also be categorized as having an eGFR ≤ 60 mL/min/1.73m2.

- **Verbal Health Literacy (Low vs. High)**
    - This distinction for subgroups is determined by participants’ responses to the question on the BRIEF Health Literacy Screening Tool.
    - The specific survey question is “How often do you have a problem understanding what is told to you about your medical condition?”. The response options include:
        {1} Always
        {2} Often
        {3} Sometimes
        {4} Occasionally
        {5} Never
    - Participants whose responses fall into categories 1-3 are considered to have “Low” verbal health literacy, while those in categories 4-5 are considered to have “High” verbal health literacy.

- **Other Health Literacy (Low vs. High)**
    - This distinction for subgroups is determined by participants’ responses to the three questions on the BRIEF Health Literacy Screening Tool, excluding the question on verbal health literacy mentioned earlier.
    - These three questions are:
        - ✓ “How often do you have someone (like a family member, friend, hospital/clinic worker or caregiver) help you read medical materials? “

{1} Always
{2} Often
{3} Sometimes
{4} Occasionally
{5} Never

- ✓ "How often do you have problems learning about your medical conditions because of difficulty understanding written information?"
  {1} Always
  {2} Often
  {3} Sometimes
  {4} Occasionally
  {5} Never
- ✓ "How confident are you filling out medical forms by yourself?"
  {1} Not at all
  {2} A little bit
  {3} Somewhat
  {4} Quite a bit
  {5} Extremely

- A total score on these three items can be computed if a participant has non-missing response to all three questions. The range of the total score is from 3 to 15.
- Health literacy status is categorized as follow:
  - ✓ Low health literacy: Total score ranged from 3 to 9.
  - ✓ High health literacy: Total score ranged from 10 to 15.

▪ **Education (≤ High school vs. > High school)**

- Participants' responses to the baseline question below define their education subgroups:
  - ✓ "What is the highest grade or level of school you completed or the highest degree you received?"
    {1} Less than high school (less than 9th grade)
    {2} Some high school (9th to 12th grade), no diploma
    {3} High school graduate (diploma or GED or equivalent)
    {4} Some post high school training (college or occupational, technical, or vocational training), no degree or certificate
    {5} Associate (2-year) college degree, or completed occupational, technical, or vocational program and received degree or certificate
    {6} Bachelor's degree (for example: BA, AB, BS)
    {7} Graduate or professional degree (for example: MA, MBA, JD, MD, PhD)
- Participants whose responses fall into categories 1-3 are considered to have education of high school or lower (≤ High school), while those in categories 4-7 are considered to have education of above high school (> High school).

▪ **Health Insurance (Private vs. Medicare/Medicaid vs. Other)**

- Participants' responses to the baseline question below define their insurance subgroups:
  - ✓ What kind of Health Insurance, if any, do you have right now?
    {1} No Insurance/Sliding Scale
    {2} Medicaid
    {3} Medicare
    {4} Private Health Insurance
    {5} Military Health Care/VA
    {6} Other insurance
    {888} DON'T KNOW
    {999} REFUSED

- Participants who selected category 4 as their only insurance will be classified as having "Private" health insurance.
- Participants who selected category 2 and/or category 3 will be classified under Medicare/Medicaid.
- Otherwise, participants who selected categories 1, 4, 5, or 6 and who are not included in the above two subgroups will be included in the "Other" subgroup.

▪ **Poverty Status (Poverty vs. Not Poverty)**
  - Poverty status will be determined using data from two sources:
    - ✓ Participants' responses to baseline survey questions regarding household size and household income in this study.
    - ✓ The Federal Poverty Guideline for 2020 (Source: https://aspe.hhs.gov/topics/poverty-economic-mobility/poverty-guidelines/prior-hhs-poverty-guidelines-federal-register-references). Since participants in this study were randomized between the year 2020 to 2024, we select the year 2020 to be the most conservative in terms of poverty classification.
  - The "Poverty" subgroup includes participants who are classified as "In Poverty" or "Near Poverty" according to the Federal Poverty Guideline for 2020. Near poverty is defined as 125% of the federal poverty level.
  - Participants' responses to the baseline question below define their household income status:
    - ✓ "What was your household's total family income (before taxes) from all sources in the last year?"
      {0} Less than $20,000 (or less than 1,666 per month)
      {1} $20,000 to $39,999 (or 1,666 to 3,333 per month)
      {2} $40,000 to $59,999 (or 3,333 to 4,999 per month)
      {3} $60,000 to $79,999 (or 5,000 to 6,666 per month)
      {4} $80,000 to $99,999 (or 6,666 to 8,333 per month)
      {5} $100,000 to $139,999 (or 8,333 to 11,666 per month)
      {6} $140,000 or more (or 11,666 or more per month)
  - Due to the categorization of income, the actual federal poverty guidelines cannot be directly applied to certain household income categories. For these income categories, the midpoint of the income category will be compared with the poverty thresholds to determine whether a participant falls into the "In Poverty" or "Near Poverty" category.

• **Healthcare Access (Yes vs. No):**
  - Participants' responses to the baseline question below define their subgroups:
    - ✓ "Overall, how difficult is it for you to get medical care when you need it? Would you say it is: "
      {1} Not at all difficult
      {2} A little bit difficult
      {3} Somewhat difficult
      {4} Very much difficult
      {5} Extremely difficult.
  - Participants whose responses fall into categories 1 or 2 are considered to have a "Yes" response to the healthcare access question, while those in categories 3, 4 or 5 are considered to have a "No" response to the healthcare access question.

▪ **Previous Genetic Test (Yes vs. No)**
  - The subgroups are defined by participants' responses to the baseline question below:
    - ✓ "Have you ever had a genetic test before?"

- **Medication Adherence (Non-adherent vs. Adherent)**
  - This distinction for subgroups is determined by participants' responses to three items in the Extent of Nonadherence scale of the Voils Domains of Subjective Extent of Nonadherence (DOSE-Nonadherence) measure.
  - These three questions are:
    - ✓ "I missed my (blood pressure) medicine. "
      - {1} None of the time
      - {2} A little of the time
      - {3} Some of the time
      - {4} Most of the time
      - {5} Every time
    - ✓ "I skipped a dose of my (blood pressure) medicine."
      - {1} None of the time
      - {2} A little of the time
      - {3} Some of the time
      - {4} Most of the time
      - {5} Every time
    - ✓ "I did not take a dose of my (blood pressure) medicine."
      - {1} None of the time
      - {2} A little of the time
      - {3} Some of the time
      - {4} Most of the time
      - {5} Every time
  - For participants who responded to at least two out of the above three items, their responses to these three items are dichotomized using the following algorithm:
    - ✓ If a participant has a score of 2-5 on any of the items, the participant is considered as nonadherent (e.g., coded as 1 or "Yes").
    - ✓ If a participant has a score of 1 (none of the time) on all the items, the participant is considered as adherent (e.g., coded as 0 or "No").

7.4.6 Interaction of APOL1 Phenotype and Randomized Treatment

This analysis will be performed among participants in the ITT population. The focus of this analysis is to examine the interaction between APOL1 phenotype and the randomized treatment for the primary endpoint. Specifically, we aim to explore whether treatment effects on SBP change from baseline to 3 months differ between participants with positive and negative APOL1 phenotypes. To achieve this, we will employ the same linear regression model used for the covariate-adjusted analysis for the primary endpoint, with the addition of the APOL1 phenotype factor and its interaction with the randomized treatment.

This analysis aims to examine whether the primary endpoint analysis results obtained in the mITT population (i.e., the APOL1 positive participants) are consistent with those obtained in the APOL1 negative participants.

7.4.7 Analyses on Other Continuous Blood Pressure Outcomes

Analyses on four additional blood pressure outcomes by randomized treatment groups in the mITT population will be performed:

- Change in SBP from baseline to 6 months
- Change in DBP from baseline to 3 months
- Change in DBP from baseline to 6 months
- Change in SBP from baseline to 3 months after excluding SBP obtained from EHR

These analyses will be performed utilizing the mixed-effect model for repeated measures (MMRM). The blood pressure data observed at all the follow-up time-points (i.e., 3 months and 6 months) will be included, and the time-point will be treated as a categorical variable in the model. The MMRM analyses will incorporate adjustments for baseline blood pressure and other baseline covariates pre-specified for the primary endpoint model, as detailed in Section 7.2.1.

7.4.8 Blood Pressure Control Outcome

Blood pressure (BP) control is a binary (yes/no) variable that is defined as:

- BP Control = yes, if SBP < 140 mmHg and DBP < 90 mmHg.
- BP Control = no (or uncontrolled BP), if SBP ≥ 140 mmHg or DBP ≥ 90 mmHg.

Data on blood pressure (BP) control status (yes/no) at 3-month and 6-month visits between the two treatment groups will be summarized.

Descriptive summary of the change in blood pressure (BP) control from baseline to the 3-month visit for the two randomized treatment groups, categorized as follows, will be provided:

- Uncontrolled BP at baseline → Controlled BP at 3-month visit
- Controlled BP at baseline → Uncontrolled BP at 3-month visit
- Uncontrolled BP at baseline → Uncontrolled BP at 3-month visit
- Controlled BP at baseline → Controlled BP at 3-month visit

The blood pressure (BP) control status (yes/no) at each follow-up visits and the change in BP control status (yes/no) from baseline to 3- and 6-minth visits will be compared between the two randomized treatment groups using the Generalized Estimating Equation (GEE) method. This analysis will adjust for the same covariates included in the covariate-adjusted model for the primary endpoint.

7.4.9 Psycho-Behavioral Outcomes of Participants

Data on participants' perspectives on study participation, APOL1 testing, and result sharing will be summarized and compared between the two randomized treatment groups. This comparison will be conducted using the Chi-square test or Fisher's Exact test, as appropriate. The data for this analysis is derived from participants' responses to the following 3-month survey questions:

- Had enough information to decide about joining the study and getting tested for APOL1.
- The information about the study, the APOL1 test and the result was easy to understand.

Similar analyses will be performed to assess whether providing participants with APOL1 testing and return of results (ROR) leads to positive behavioral changes that could enhance blood pressure management and reduce

the risk of kidney disease. Specifically, we will compare participants' self-reported data on the 3-month and/or 6-month survey questions below between the two treatment groups:

- Made positive lifestyle changes on either of the two items below:
  - Become more physically active or exercise more.
  - Make healthier changes to your diet in the past 3 months.
- Changed how you take BP medication.
- Take BP medication more often.

7.4.10 Potential Post-hoc Analyses

While there are no known systematic disruptions to randomization in the trial conduct, patterns underlying missing data in the primary endpoint and the genotype results will be assessed for systematic differences between the two randomized treatment groups that persist across the entire trial period after unblinding. If evidence of such systematic differences arises, post-hoc analyses with adjustments may be considered.

7.5 Handling of Missing Data

If more than 10% of participants in the mITT population have missing data on the primary endpoint (i.e., SBP change from baseline to 3 months), sensitivity analysis will be performed using data obtained from multiple imputation. This sensitivity analysis will be performed only for the primary analysis and the covariate-adjusted analysis for primary endpoint.

Multiple imputation will be used to impute the missing baseline and 3-month SBP values. All observed data at baseline and 3 months will be included in the imputation model, except for participants who died before the due date of Month 3 visit. SAS PROC MI procedure will be used for multiple imputations. Specifically, full condition specification (FCS) method will be used to impute missing values. Baseline SBP and other baseline covariates as specified for the covariate-adjusted analysis for the primary endpoint will be included as the covariates in the imputation model. The multiple imputation algorithm will create 20 complete datasets. The results across the 20 imputed datasets will be combined using Rubin's rule (Rubin, 1987) to obtain one set of results for the primary analysis and covariate-adjusted analysis on the primary endpoint.

Multiple imputation analyses are based on the assumption that data are missing at random (MAR). To ensure that the missing at random (MAR) assumption for multiple imputation is valid, we will compare baseline SBP and other participant characteristics among mITT participants who have non-missing data on SBP change from baseline to 3 months and those who have missing data on that SBP change. This will help us to check whether there is any indication of data missing not at random (MNAR). If more than 10% of mITT participants have missing data on SBP change from baseline to 3 months, and if these participants with missing data are significantly different from other participants, sensitivity analysis using tipping point method will be performed to further test the robustness of the primary endpoint analysis results obtained with multiple imputation. In the tipping point analysis, missing data are imputed over a range of possible scenarios for the treatment effect. We will assume progressively biased imputation for SBP at 3 months varying from the worst SBP favoring control arm to the best SBP at 3 months. For each scenario, p-value for t-test that compares the SBP change from baseline to

3 months between two treatment arms will be reported. The mean and 95% CI in the mean difference between two treatment groups in SBP change from baseline to 3 months for each scenario will also be presented. This is done in order to identify the scenario or 'tipping point' where the treatment effect in subjects with missing data overturns the significant or non-significant treatment effect obtained in the analyses using multiple imputation assuming MAR. We will then use our knowledge of participant population to assess the plausibility of the tipping point scenario. If considered unlikely, this provides support for the treatment effect found in the MAR analysis.

8 Safety Evaluation

GUARDD-US is a study that does not include drug or device intervention. It presents minimal risks to participants. For this reason, adverse event data are not systematically collected in the study database. The following safety data will be presented by randomized treatment for safety evaluation for participants in mITT and ITT populations:

- Distress from return of results
- Early termination and discontinuation
- Events of interest as reported in EHR data (including emergency department visits and hospitalizations)
- Adverse events suspected to be related to study interventions
- Unanticipated study related deaths
- Participants with very high blood pressure (i.e., the third of three consecutively measured blood pressure readings is greater than or equal to 190 mmHg for SBP or greater than or equal to 110 mmHg for DBP) at baseline and each of the follow-up time points (i.e., 3 months and 6 months)

Safety evaluation will be performed among all participants in the mITT and ITT populations. Participants' actual treatment received will be presented.

9 References

E9(R1) Statistical Principles for Clinical Trials: Addendum: Estimands and Sensitivity Analysis in Clinical Trials

"Genetic Testing to Understand and Address Renal Disease Disparities across the United States (GUARDD-US)" Protocol, Version 5.0 2022

Jennison, C., and Turnbull, B.W. 2000. Group Sequential Methods with Applications to Clinical Trials. Chapman & Hall/CRC. New York.

Rubin DB. 1987. Multiple Imputation for Nonresponse in Surveys. John Wiley, New York.

Inker LA, Levey AS, Tighiouart H, et al. New Creatinine- and Cystatin C–Based Equations to Estimate GFR without Race. N Engl J Med [Internet] 2021 [cited 2023 Aug 4]; 385:328-39. Available from: https://www.nejm.org/doi/full/10.1056/NEJMoa2023182
