# Supplementary material for: Genetic Testing for APOL1 in Adults With Hypertension: The GUARDD-US Randomized Clinical Trial
Source: JAMA Netw Open. 2026 Mar 5;9(3):e260528. doi: 10.1001/jamanetworkopen.2026.0528 (PMC12964156; doi:10.1001/jamanetworkopen.2026.0528)
Supplement: Supplement 2. — eAppendix 1. GUARDD-US Clinical Groups eAppendix 2. GUARDD-US Protocol Implementation Teams eAppendix 3. GUARDD-US Site PIs and Site Study Staff eAppendix 4. Additional Collaborators eTable 1. Participant Characteristics of ITT Population eTable 2. Participant Characteristics of APOL1-HR, APOL1-HR With Uncontrolled BP and APOL1-HR With Controlled BP eTable 3. Description of Antihypertensive Medication Use at Enrollment, 3 Months, and 6 Months mITT and Uncontrolled BP eTable 4. Blood Pressure Outcomes at 3 Months and 6 Months, APOL1-HR Population eTable 5. Sensitivity Analysis of Primary Outcome With Removal of EHR-Derived Measures eTable 6. Controlled and Uncontrolled Systolic Blood Pressure Analysis eTable 7. Secondary Chronic Kidney Disease-Related Outcomes at 6 Months eTable 8. Perspectives of Trial Participants on Sharing Genotype Results and Self-Reported Behaviors eFigure 1. Per-Protocol Subgroup Analyses of Treatment Group Difference in Systolic Blood Pressure Change From Baseline to 3 Months eFigure 2. Exploratory Subgroup Analyses of Treatment Group Difference in Systolic Blood Pressure Change From Baseline to 3 Months [file jamanetwopen-e260528-s002.pdf]

Supplementary Online Content

Eadon MT, Cavanaugh KL, She L, et al; Implementing Genomics in Practice (IGNITE) Pragmatic Trials Network. Genetic testing for *APOL1* in adults with hypertension: the GUARDD-US randomized clinical trial. *JAMA Netw Open.* 2026;9(3):e260528. doi:10.1001/jamanetworkopen.2026.0528

eAppendix 1. GUARDD-US Clinical Groups
eAppendix 2. GUARDD-US Protocol Implementation Teams
eAppendix 3. GUARDD-US Site PIs and Site Study Staff
eAppendix 4. Additional Collaborators
eTable 1. Participant Characteristics of ITT Population
eTable 2. Participant Characteristics of *APOL1*-HR, *APOL1*-HR With Uncontrolled BP and *APOL1*-HR With Controlled BP
eTable 3. Description of Antihypertensive Medication Use at Enrollment, 3 Months, and 6 Months mITT and Uncontrolled BP
eTable 4. Blood Pressure Outcomes at 3 Months and 6 Months, *APOL1*-HR Population
eTable 5. Sensitivity Analysis of Primary Outcome With Removal of EHR-Derived Measures
eTable 6. Controlled and Uncontrolled Systolic Blood Pressure Analysis
eTable 7. Secondary Chronic Kidney Disease-Related Outcomes at 6 Months
eTable 8. Perspectives of Trial Participants on Sharing Genotype Results and Self-Reported Behaviors
eFigure 1. Per-Protocol Subgroup Analyses of Treatment Group Difference in Systolic Blood Pressure Change From Baseline to 3 Months
eFigure 2. Exploratory Subgroup Analyses of Treatment Group Difference in Systolic Blood Pressure Change From Baseline to 3 Months

This supplementary material has been provided by the authors to give readers additional information about their work.

© 2026 Eadon MT et al. *JAMA Network Open.*

eAppendix 1. GUARDD-US Clinical Groups

Clinical Group Award PI: Duke University Medical Center: Durham, NC: Orlando, L

- Duke University Medical Center, Durham, NC: Orlando, L
- Baylor Research Institute, Dallas, TX: Kitzman, H, Dodgen, L
- Louisiana Public Health Institute, New Orleans, LA: Nauman, B
- Southeastern Healthcare, Lumberton, NC: Roberts, J
- University Medical Center – New Orleans, New Orleans, LA: Fuloria, J
- University of North Carolina – Pembroke, Pembroke, NC: Beasley, C

Clinical Group Award PI: Icahn School of Medicine at Mount Sinai, New York, NY: Horowitz, C

- Icahn School of Medicine at Mount Sinai, New York, NY: Horowitz, C
- The Institute for Family Health, New York, NY: Calman, N

Clinical Group Award PI: Indiana University Health System, Indianapolis, IN: Skaar, T

- Indiana University Health System, Indianapolis, IN: Eadon, M
- Eskenazi Health, Indianapolis, IN: Eadon, M
- University of Pittsburgh/UPMC, Pittsburgh, PA: Empey, P

Clinical Group Award PI: University of Florida, Gainesville, FL: Johnson, J

- University of Florida, Gainesville, FL: Cooper-DeHoff, R
- University of Florida, Jacksonville, FL: Cooper-DeHoff, R
- University of Alabama, Birmingham, Birmingham, AL: Limdi, N

Clinical Group Award PI: Vanderbilt University Medical Center, Nashville, TN: Peterson, J

- Vanderbilt University Medical Center, Nashville, TN: Cavanaugh, K
- Meharry Medical College, Nashville, TN: Singh, R
- Nashville General, Nashville, TN: Singh, R

IGNITE Coordinating Center: Duke Clinical Research Institute, Durham, NC: Wyatt, C, Chakraborty, H

© 2026 Eadon MT et al. *JAMA Network Open.*

eAppendix 2. GUARDD-US Protocol Implementation Teams

IGNITE Coordinating Center: Rishi Chakraborty (PI), Christina Wyatt (PI), Geoff Ginsburg (*f*-PI), Kady-Ann Steen-Burrell, Bhargav Adagarla, Sarah George, Rachel Myers, Elizabeth Harris, Jaclyn Holland, Yashika Johnson, Phyllis Kennel, Kristen Linney, Peter Merrill, Rania Metry, Gayle Passmore, Carol Pereira, Teji Rakhra-Burris, Lilin She, Jennifer Shepherd and Saira Siddiqui

Duke University Medical Center: Lori Orlando *(CG PI)*, Nancy Garrett-Mead *(Duke)*, Teji Rakhra-Burris *(Duke)*, Beth Nauman *(site PI – LPHI)*, Meagan Alley *(LPHI)*, Erica Johnson (*LPHI*), Jyotsna Fuloria (*site PI—UMC-NO*), Emily Bozant (*UMC-NO),* Brianne Voros (*UMC-NO*)Cherry Beasley *(site PI – UNC-P),* Neatte Ridgeway *(UNC-P)*, Joseph Roberts *(site PI – SE)*, Leilani Dodgen *(site PI – BSW), (BSW)*, Patricia Allison *(BSW),* Heather Kitzman, Joycelyn Larbie *(BSW*), Aisha Montgomery (*BSW)* and Meera Patel

Icahn School of Medicine at Mount Sinai: Carol Horowitz *(PI)*, Girish Nadkarni *(co-I)*, Dinushika Mohottige, Michelle Ramos, Sabrina Clermont, Diane Hauser, Janet Seo, Nandini Shroff, Saskia Shuman, and Randi Zinberg

Indiana University Health System: Michael Eadon *(site PI – IU)*, Jennifer Stuart, Travis Beamon, Zachary Cowsert, Stephanie Eadon, Jennelle Hodge, Jared Lovins, Sheryl Lynch, Kelsey McClara, Vicky Pratt, Emma Tillman, Philip Empey *(site PI – Pittsburgh)*, Mylynda Massart *(Pittsburgh)* and Linda Prebehalla *(Pittsburgh)*

University of Florida: Rhonda Cooper-DeHoff *(site PI – UF)*, Elizabeth Eddy, Erica Elwood, Ryan Rhoden, Adeia Williams, Nita Limdi *(site PI – UAB),* Bethany Etheridge *(UAB),* Blake Goff *(UAB)* and Lynn Holtam *(UAB)*

Vanderbilt University Medical Center: Kerri Cavanaugh *(CG PI)*, Sara Block, Henry Ong, Sarah Pleasant, Rajbir Singh *(site PI – Meharry)*, Sidd Pratap (*Meharry)* and Margaret Trietsch (*Meharry*)

NHGRI: Simona Volpi, Sarah Hutchison, Natalie Kucher, Ebony Madden, Jessica Reinach, Renee Rider, Ismail Safi and Ella Samer

© 2026 Eadon MT et al. *JAMA Network Open.*

eAppendix 3. GUARDD-US Site PIs and Site Study Staff

Baylor Scott & White Research Institute: Leilani Dodgen (PI), Heather Kitzman-Carmichael (*f*-PI), Patricia Allison, Lydia Best, Kierra Bennett, Libertad Gracia, Joycelyn Larbie, Aisha Montgomery, Brenda Olivares, Meera Patel, Carol Patterson, Emilie Ruiz, Anne Marie Strauss, Tenira Stubblefield, Doris Webb and Emily Villegas

Duke University Medical Center: Nancy Garrett-Mead and Azita Sadeghpour

Icahn School of Medicine at Mount Sinai and/or The Institute of Family Health: Carol Horowitz (PI), Michelle Ramos, Sabrina Clermont, Daviana Buck, Nicole Canchucaja, Christina Carranceja, David Christian, Tyler Colon, Porshia Cook, Aaishah Francis, Kevin Gernavage, Erika Gonzalez, Samantha Guagliardo, Diane Hauser, Shamara Henry, Ariel Lindsay Jacobs, Danielle John, Najiba Khan, Camila Tan Lam, Kara Lindsay, Emma Maiman-Stadtmauer, Sofia Medina-Pardo, Mirta Milanes, Wambui Ngari, Tatiana Sabin, Samantha St. Elin, Michelle Sciarrino, Nandini Shroff, Saskia Shuman, Nicole Simons and Lola Williams

IGNITE Coordinating Center: Rishi Chakraborty (PI), Christina Wyatt (PI), Geoff Ginsburg (*f*-PI), Sheng Luo, Kady-Ann Steen-Burrell, Kevin Anstrom, Bhargav Adagarla, Alicia Ellis, Stephen Ellis, Sarah George, Beth Harris, Jaclyn Holland, Yashika Johnson, Phyllis Kennel, Hwasoon Kim, Kristen Linney, Peter Merrill, Rania Metry, Rachel Myers, Wanda Parker, Gayle Passmore, Carol Pereira, Teji Rakhra-Burris, Lilin She, Jennifer Shepherd, Saira Siddiqui, and Jun Wen

Indiana University and Eskenazi Health: Michael Eadon (PI), Travis Beamon, Christian Brummett, Zachary Cowsert, Brittany Davis, Stephanie Eadon, Makenzie Gee, John Howard, Alia Jamison, Lydia Joyner, Gabrielle Kline, Jared Lovins, Sheryl Lynch, Kelsey McClara, Johnathan Oliver, Jefney Ongeri, Krina Patel, Kristina Perez, Adam Ruhayel, Nehal Sheth, Libbie Silverman, Jordan Sonneville, Ashley Vetor and Alex Woodcock

Meharry Medical College and Nashville General Hospital: Rajbir Singh (PI), Megan Trietsch, Anaite Montes Bu, Sylvia Eluhu, Abraham Garcia, Carol Gutierrez, Steven Houtschilt, Maria Lopez, Allan Mejia, Nimrit Mokha, Kimberly Snell, Ana Tomescu, Deanna Webb and Sonja White

University Medical Center – New Orleans: Domnic Bett, Emily Bozant, Ranielle Emnace, Alexandria Henderson, Tae'lar Henry, Pauline Pastore, Wayne Swink, Alexander Vandeerlin, Brianne Voros and Madeline Young

University of Alabama at Birmingham: Nita Limdi (PI), Bethany Etheridge, Josh Cortopassi, Brittney Davis, Chrisly Dillon, Edna Fields, Stephanie Ford, April Garrett, Blake Goff, Emory Heffernan, Mickey Holley, Robert Holtam, Jonjerica Lucky, Tyesha Smith and Lorenzo Thompson

University of Florida at Gainesville and/or at Jacksonville: Rhonda Cooper-DeHoff (PI), Alexander Parker (PI), Erica Elwood, Elizabeth Eddy, Jose Alonso, Tala Basha, Maria Bautista, Emily Cicali, Asia Cobb, Brendon Cooper, Karla Giron, Anuksha Gotmare, Fatoumata Kaba, Alexander Litvintchouk, Ricky Mareus, Shivani Mehta, Arielle Nelson, Khoa Nguyen, Olinda Nichols, Anthony Quero, Ryan Rhoden, Genevieve Rosier, Joshua Terrell, Claudia Cartaya Torres, Kimberly Vigal, ADeia Williams and Precious Williams

© 2026 Eadon MT et al. *JAMA Network Open.*

University of Pittsburgh: Phil Empey (PI), Mylynda Massart (PI), Kristine Daw, Erika Dreidorn, Alexandra Mykita and Linda Prebehalla

Southeastern Healthcare: Cherry Beasley (PI), Te'Kayla Alexander, Jocelyn Bullard, Jason Butler, Tierra Chavis, Paige Chmura, Lori Dove, Autumn Hair, Chelsey Lowery, Tymia McNeil, Jasmine Powell, Asa Revels, Neatte Ridgeway, Cheryl Tschopp, Alexis Turner and Summer Williamson

Vanderbilt University Medical Center: Kerri Cavanaugh (PI), Sara Block, Chantel Bender, Henry Ong, Hadassah Pegues, Kendra Phillips, Sarah Pleasant, Chris Roach, Christina Ross, Ebele Umeukeje and Sonya Williams

© 2026 Eadon MT et al. *JAMA Network Open.*

eAppendix 4. Additional Collaborators

Irfan Asif, MD, Nader Bahri, MD, Amy M. Breman, John Thomas Callaghan, Gajapathiraju Chamarthi, MD, Ku Chang, Erin Delaney, MD, Makenzie Gee, Daniel Getu, MD, Sarah Gopman, Melanie Gross Hagen, MD, Judive Jocelyn, DO, Ross Jones, MD, Joseph Kannry, MD, Wai Lang Lau, MD, Jeffrey Leegon, MS, Edlira Maska, Elvira Silveria Mercado, MD, Ying Lu Nagoshi, MD, Thomas D. Nolin, Olanrewaju Adebayo Olaoye, MD, Frank Orlando, MD, Brandi Plunkett, Siddarth Pratap, PhD, RN, Ebony Pratt, MD, Victoria M. Pratt, Maria P. Robles, Siegfried Schmidt MD, S. Jawad Sher, Arjun D. Sinha, Mircea Sorin, MD, Eric Stewart, MD, Evgenia Teal, Deanna R. Willis

© 2026 Eadon MT et al. *JAMA Network Open.*

eTable 1. Participant Characteristics of ITT Population

	**ITT, All Participants (N=6754)**	**ITT Immediate *APOL1* ROR, Intervention (N=3380)**	**ITT Delayed *APOL1* ROR, Control (N=3374)**
APOL1 phenotype, no./No., (%)			
APOL1 high risk/positive	954/6704 (14.2%)	486/3365 (14.4%)	468/3339 (14.0%)
APOL1 not high risk/negative	5750/6704 (85.8%)	2879/3365 (85.6%)	2871/3339 (86.0%)
Source of baseline SBP data, no. (%)			
Baseline SBP, in-person	6746 (99.9%)	3373/3380 (99.8%)	3373/3374 (>99.9%)
Baseline SBP, medical record	7 (0.1%)	6/3380 (0.2%)	1/3374 (<0.1%)
3-month visit SBP, in-person, no. (%)	5815 (86.1%)	2880/3380 (85.2%)	2935/3374 (87.0%)
3-month visit SBP, medical record, no. (%)	347 (5.1%)	184/3380 (5.4%)	163/3374 (4.8%)
6-month visit SBP, in-person, no. (%)	5561 (82.3%)	2762/3380 (81.7%)	2799/3374 (83.0%)
6-month visit SBP, medical record, no. (%)	401 (5.9%)	203/3380 (6.0%)	198/3374 (5.9%)
Completed both 3-month and 6-month visit SBP, no. (%)	5695 (84.3%)	2825/3380 (83.6%)	2870/3374 (85.1%)
Age at randomization, mean±SD, yrs	55.3±10.3	55.3±10.3	55.4±10.2
Sex at birth, no./No. (%)			
Female	4310/6753 (63.8%)	2178/3380 (64.4%)	2132/3373 (63.2%)
Male	2443/6753 (36.2%)	1202/3380 (35.6%)	1241/3373 (36.8%)
Self-identified race/ethnicity, no./No. (%)			
American Indian, Native American or Alaska Native	19/6753 (0.3%)	10/3379 (0.3%)	9/3374 (0.3%)
Asian	1/6753 (<0.1%)	1/3379 (<0.1%)	0/3374 (0.0%)
Black or African American	6113/6753 (90.5%)	3071/3379 (90.9%)	3042/3374 (90.2%)
Native Hawaiian or Other Pacific Islander	4/6753 (0.1%)	3/3379 (0.1%)	1/3374 (<0.1%)
White or European American	1/6753 (<.0.1%)	0/3379 (0.0%)	1/3374 (<0.1%)

© 2026 Eadon MT et al. *JAMA Network Open.*

Middle Eastern or North African/Mediterranean	2/6753 (<0.1%)	2/3379 (0.1%)	0/3374 (0.0%)
Hispanic/Latino(a)	58/6753 (0.9%)	31/3379 (0.9%)	27/3374 (0.8%)
Multiple	453/6753 (6.7%)	215/3379 (6.4%)	238/3374 (7.1%)
Other, unknown, prefer not to answer	102/6753 (1.5%)	46/3379 (1.4%)	56/3374 (1.7%)
Education–more than high school, no./No. (%)	4141/6726 (61.6%)	2072/3367 (61.5%)	2069/3359 (61.6%)
Household income <$40,000/year, no./No. (%)	2782/5171 (53.8%)	1385/2588 (53.5%)	1397/2583 (54.1%)
Insurance, no./No. (%)			
No insurance	464/6710 (6.9%)	225/3364 (6.7%)	239/3346 (7.1%)
Medicaid*	1335/6710 (19.9%)	704/3364 (20.9%)	631/3346 (18.9%)
Medicare*	1042/6710 (15.5%)	523/3364 (15.5%)	519/3346 (15.5%)
Medicare and Medicaid	729/6710 (10.9%)	357/3364 (10.6%)	372/3346 (11.1%)
Private health insurance only	2510/6710 (37.4%)	1230/3364 (36.6%)	1280/3346 (38.3%)
Other insurance (including military health care/VA)	630/6710 (9.4%)	325/3364 (9.7%)	305/3346 (9.1%)
Poverty, no./No. (%)	1721/5156 (33.4%)	851/2584 (32.9%)	870/2572 (33.8%)
Current tobacco use, no./No. (%)	1545/6746 (22.9%)	761/3377 (22.5%)	784/3369 (23.3%)
BP medication use at enrollment, no. (%)	6329/6754 (93.7%)	3159/3380 (93.5%)	3170/3374 (94.0%)
Enrollment SBP, mean±SD, mm Hg	133.5±19.3 (n=6746)	133.5±19.2 (n=3373)	133.6±19.4 (n=3373)
Enrollment DBP, mean±SD, mm Hg	83.1±13.2 (n=6746)	83.3±13.1 (n=3373)	82.9±13.3 (n=3373)
SBP ≥140 mm Hg, no./No. (%)	2133/6746 (31.6%)	1073/3373 (31.8%)	1060/3373 (31.4%)
DBP ≥90 mm Hg, no./No. (%)	1807/6746 (26.8%)	924/3373 (27.4%)	883/3373 (26.2%)
SBP <140 and DBP <90 mm Hg, no./No. (%)	4047/6746 (60.0%)	2004/3373 (59.4%)	2043/3373 (60.6%)
Diabetes diagnosis†, no./No. (%)	1404/6156 (22.8%)	691/3085 (22.4%)	713/3071 (23.2%)
CKD diagnosis‡, no./No. (%)	1887/6168 (30.6%)	934/3086 (30.3%)	953/3082 (30.9%)

*Regardless of any additional coverage by private insurance or other insurance.
†Medical record diagnosis.
‡Medical record diagnosis, Stage 1–4.
BP indicates blood pressure; CKD, chronic kidney disease; DBP, diastolic blood pressure; ITT, intention-to-treat; ROR, return of *APOL1* testing results; SBP, systolic blood pressure; SD, standard deviation; VA, Veterans Administration.

© 2026 Eadon MT et al. *JAMA Network Open.*

eTable 2. Participant Characteristics of *APOL1*-HR, *APOL1*-HR With Uncontrolled BP and *APOL1*-HR With Controlled BP

	***APOL1*-HR, All Participants (N=954)**	***APOL1*-HR, with Uncontrolled BP, All Participants[*] (N=377)**	***APOL1*-HR with Controlled BP, All Participants (N=575)**	***APOL1*-HR with Controlled BP**	
				**Immediate *APOL1* ROR, Intervention (N=291)**	**Delayed *APOL1* ROR, Control (N=284)**
**Visit Completion of BP Measurement**					
Baseline SBP, in-person	952/954 (99.8%)	377/377 (100%)	575/575 (100%)	291/291 (100%)	284/284 (100%)
Baseline SBP, medical record	1/954 (0.1%)	0 (0%)	0 (0%)	0 (0%)	0 (0%)
3-month visit SBP, in-person	833/954 (87.3%)	327/377 (86.7%)	505/575 (87.8%)	254/291 (87.3%)	251/284 (88.4%)
3-month visit SBP, medical record	42/954 (4.4%)	14/377 (3.7%)	28/575 (4.9%)	14/291 (4.8%)	14/284 (4.9%)
6-month visit SBP, in-person	782/954 (82.0%)	306/377 (81.2%)	475/575 (82.6%)	236/291 (81.1%)	239/284 (84.2%)
6-month visit SBP, medical record	53/954 (5.6%)	19/377 (5.0%)	34/575 (5.9%)	16/291 (5.5%)	18/284 (6.3%)
Completed both 3-month and 6-month visit SBP	801/954 (84.0%)	309/377 (82.0%)	491/575 (85.4%)	244/291 (83.8%)	247/284 (87.0%)
Age at randomization, mean±SD, yrs	54.9±10.0	53.6±10.2	55.8±9.8	55.6±9.7	56.0±10.0
Sex at birth					
Female	600/954 (62.9%)	233/377 (61.8%)	367/575 (63.8%)	196/291 (67.4%)	171/284 (60.2%)
Male	354/954 (37.1%)	144/377 (38.2%)	208/575 (36.2%)	95/291 (32.6%)	113/284 (39.8%)
Self-identified race/ethnicity					
American Indian, Native American or Alaska Native	2/954 (0.2%)	1/377 (0.3%)	1/575 (0.2%)	0 (0%)	1/284 (0.4%)
Asian	0 (0%)	0 (0%)	0 (0%)	0 (0%)	0 (0%)
Black or African American	879/954 (92.1%)	346/377 (91.8%)	531/575 (92.3%)	273/291 (93.8%)	258/284 (90.8%)
Native Hawaiian or Other Pacific Islander	2/954 (0.2%)	1/377 (0.3%)	1/575 (0.2%)	1/291 (0.3%)	0 (0%)
White or European American	0 (0%)	0 (0%)	0 (0%)	0 (0%)	0 (0%)
Middle Eastern or North African/Mediterranean	1/954 (0.1%)	0 (0%)	1/575 (0.2%)	1/291 (0.3%)	0 (0%)
Hispanic/Latino(a)	2/954 (0.2%)	0 (0%)	2/575 (0.3%)	1/291 (0.3%)	1/284 (0.4%)
Multiple	51/954 (5.3%)	21/377 (5.6%)	30/575 (5.2%)	10/291 (3.4%)	20/284 (7.0%)
Other, unknown, prefer not to answer	17/954 (1.8%)	8/377 (2.1%)	9/575 (1.6%)	5/291 (1.7%)	4/284 (1.4%)
Education–more than high school	547/949 (57.6%)	210/374 (56.1%)	335/573 (58.5%)	166/289 (57.4%)	169/284 (59.5%)
Household income <$40,000/year	411/737 (55.8%)	170/298 (57.0%)	240/437 (54.9%)	124/207 (59.9%)	116/230 (50.4%)
Insurance					
No insurance	77/949 (8.1%)	49/375 (13.1%)	28/572 (4.9%)	12/288 (4.2%)	16/284 (5.6%)
Medicaid[†]	199/949 (21.0%)	77/375 (20.5%)	121/572 (21.2%)	76/288 (26.4%)	45/284 (15.8%)
Medicare[†]	139/949 (14.6%)	43/375 (11.5%)	96/572 (16.8%)	47/288 (16.3%)	49/284 (17.3%)
Medicare and Medicaid	100/949 (10.5%)	25/375 (6.7%)	75/572 (13.1%)	35/288 (12.2%)	40/284 (14.1%)
Private health insurance only	336/949 (35.4%)	139/375 (37.1%)	197/572 (34.4%)	90/288 (31.1%)	107/284 (37.7%)
Other insurance (including military health care/VA)	98/949 (10.3%)	42/375 (11.2%)	55/572 (9.6%)	28/288 (9.7%)	27/284 (9.5%)
Poverty	250/734 (34.1%)	104/296 (35.1%)	145/436 (33.3%)	75/207 (36.2%)	70/229 (30.6%)
Current tobacco use	213/952 (22.4%)	84/376 (22.3%)	129/574 (22.5%)	72/291 (24.7%)	57/283 (20.1%)
BP medication use at enrollment	900/954 (94.3%)	351/377 (93.1%)	547/575 (95.1%)	280/291 (96.2%)	267/284 (94.0%)

© 2026 Eadon MT et al. *JAMA Network Open.*

Baseline SBP, mean±SD, mm Hg	133.1±19.2 (n=952)	149.8±17.1 (n=377)	122.2±10.8 (n=575)	121.7±11.1 (n=291)	122.7±10.5 (n=284)
Baseline DBP, mean±SD, mm Hg	83.0±12.8 (n=952)	93.3±11.6 (n=377)	76.2±8.2 (n=575)	76.6±7.9 (n=291)	75.8±8.4 (n=284)
SBP ≥140 mm Hg	285/952 (29.9%)	285/377 (75.6%)	---	---	---
DBP ≥90 mm Hg	254/952 (26.7%)	254/377 (67.4%)	---	---	---
SBP <140 and DBP <90 mm Hg	575/952 (60.4%)	---	100%	100%	100%
Diabetes diagnosis[‡]	203/887 (22.9%)	79/353 (22.4%)	123/532 (23.1%)	61/269 (22.7%)	62/263 (23.6%)
CKD diagnosis[§]	312/887 (35.2%)	125/352 (35.5%)	186/533 (34.9%)	92/271 (33.9%)	94/262 (35.9%)

Data presented as no./No. (%), unless otherwise indicated.

[*]Participants with baseline SBP ≥140 mm Hg or DBP ≥90 mm Hg; two participants have missing BP data at baseline.

[†]Regardless of any additional coverage by private insurance or other insurance.

[‡]Medical record diagnosis.

[§]Medical record diagnosis, Stage 1–4.

BP indicates blood pressure; CKD, chronic kidney disease; DBP, diastolic blood pressure; ROR, return of *APOL1* testing results; SBP, systolic blood pressure; SD, standard deviation; VA, Veterans Administration.

© 2026 Eadon MT et al. *JAMA Network Open.*

eTable 3. Description of Antihypertensive Medication Use at Enrollment, 3 Months, and 6 Months mITT and Uncontrolled BP

	***APOL1*-HR, All Participants (N=954)**						**Uncontrolled Baseline Blood Pressure in *APOL1*-HR (N=377)**					
	**Immediate *APOL1* ROR, Intervention (N=486)**			**Delayed *APOL1* ROR, Control (N=468)**			**Immediate *APOL1* ROR, Intervention (N=194)**			**Delayed *APOL1* ROR, Control (N=183)**		
**Medication Prescription, by Drug Class**												
	Baseline N=486	3-month N=452	6-month N=414	Baseline N=468	3-month N=431	6-month N=397	Baseline N=194	3-month N=181	6-month N=170	Baseline N=183	3-month N=167	6-month N=148
Thiazide	210 (43.2%)	196 (43.4%)	177 (42.8%)	171 (36.5%)	149 (34.6%)	142 (35.8%)	79 (40.7%)	73 (40.3%)	66 (38.8%)	69 (37.7%)	57 (34.1%)*	50 (33.8%)
Calcium channel blocker	259 (53.3%)	235 (52.0%)	223 (53.9%)	262 (56.0%)	242 (56.1%)	222 (55.9%)	108 (55.7%)	102 (56.4%)	101 (59.4%)	107 (58.5%)	98 (58.7%)	87 (58.8%)
Hydralazine	19 (3.9%)	17 (3.8%)	18 (4.3%)	17 (3.6%)	17 (3.9%)	15 (3.8%)	9 (4.6%)	9 (5.0%)	8 (4.7%)	14 (7.7%)*	14 (8.4%)*	12 (8.1%)*
Beta blocker	107 (22.0%)	103 (22.8%)	100 (24.2%)	135 (28.8%)	123 (28.5%)	117 (29.5%)	40 (20.6%)	38 (21.0%)	40 (23.5%)	59 (32.2%)*	53 (31.7%)	49 (33.1%)
ACE inhibitor	130 (26.7%)	126 (27.9%)	111 (26.8%)	129 (27.6%)	121 (28.1%)	111 (28.0%)	49 (25.3%)	48 (26.5%)	47 (27.6%)	49 (26.8%)	44 (26.3%)	39 (26.4%)
Angiotensin receptor blocker	132 (27.2%)	123 (27.2%)	116 (28.0%)	116 (24.8%)	108 (25.1%)	103 (25.9%)	50 (25.8%)	49 (27.1%)	48 (28.2%)	44 (24.0%)	41 (24.6%)	40 (27.0%)
Alpha blocker	3 (0.6%)	3 (0.7%)	3 (0.7%)	2 (0.4%)	2 (0.5%)	3 (0.8%)	1 (0.5%)	1 (0.6%)	1 (0.6%)	1 (0.5%)	1 (0.6%)	2 (1.4%)
Loop diuretic	33 (6.8%)	33 (7.3%)	30 (7.2%)	26 (5.6%)	20 (4.6%)	23 (5.8%)	12 (6.2%)	12 (6.6%)	13 (7.6%)	13 (7.1%)	10 (6.0%)	11 (7.4%)
K-sparing diuretic	19 (3.9%)	16 (3.5%)	16 (3.9%)	10 (2.1%)	10 (2.3%)	9 (2.3%)	5 (2.6%)	4 (2.2%)	4 (2.4%)	4 (2.2%)	4 (2.4%)	3 (2.0%)
Mineralocorticoid receptor antagonist	25 (5.1%)	22 (4.9%)	24 (5.8%)	23 (4.9%)	20 (4.6%)	20 (5.0%)	8 (4.1%)	7 (3.9%)	8 (4.7%)	8 (4.4%)	7 (4.2%)	7 (4.7%)
Vasodilator	22 (4.5%)	20 (4.4%)	21 (5.1%)	24 (5.1%)	24 (5.6%)	21 (5.3%)	11 (5.7%)	11 (6.1%)	10 (5.9%)	15 (8.2%)	15 (9.0%)*	13 (8.8%)
Alpha 2 receptor agonist	5 (1.0%)	4 (0.9%)	5 (1.2%)	9 (1.9%)	9 (2.1%)	9 (2.3%)	2 (1.0%)	2 (1.1%)	3 (1.8%)	7 (3.8%)	7 (4.2%)*	7 (4.7%)*
Direct renin inhibitor	0 (0%)	0 (0%)	0 (0%)	1 (0.2%)	1 (0.2%)	1 (0.3%)	0 (0.0%)	0 (0.0%)	0 (0.0%)	1 (0.5%)	1 (0.6%)	1 (0.7%)
SGLT2 inhibitor†	5 (1.0%)	5 (1.1%)	5 (1.2%)	3 (0.6%)	5 (1.2%)	5 (1.3%)	3 (1.5%)	3 (1.7%)	3 (1.8%)	1 (0.5%)	2 (1.2%)	1 (0.7%)
**Number of Antihypertensive Medications**												
0	23 (4.7%)	19 (4.2%)	19 (4.6%)	31 (6.6%)	25 (5.8%)	23 (5.8%)	12 (6.2%)	12 (6.6%)	10 (5.9%)	14 (7.7%)	11 (6.6%)	10 (6.8%)

© 2026 Eadon MT et al. *JAMA Network Open.*

	***APOL1*-HR, All Participants (N=954)**						**Uncontrolled Baseline Blood Pressure in *APOL1*-HR (N=377)**					
1	152 (31.3%)	145 (32.1%)	122 (29.5%)	151 (32.3%)	143 (33.2%)	121 (30.5%)	67 (34.5%)	59 (32.6%)	51 (30.0%)	50 (27.3%)	48 (28.7%)	36 (24.3%)
2	180 (37.0%)	162 (35.8%)	149 (36.0%)	145 (31.0%)	138 (32.0%)	135 (34.0%)	60 (30.9%)	54 (29.8%)	51 (30.0%)	58 (31.7%)	57 (34.1%)	56 (37.8%)
≥ 3	131 (27.0%)	126 (27.9%)	124 (30.0%)	141 (30.1%)	125 (29.0%)	118 (29.7%)	55 (28.4%)	56 (30.9%)	58 (34.1%)	61 (33.3%)	51 (30.5%)	46 (31.1%)
**Number of antihypertensives, mean±SD**	1.97±1.1	1.97±1.1	2.02±1.1	1.96±1.1	1.94±1.1	1.99±1.1	1.91±1.1	1.94±1.1	2.04±1.1	2.08±1.3	2.05±1.2	2.10±1.2
**Changes in Number of Antihypertensive medications, Baseline to 3-months, and Baseline to 6-months**												
Group	**Immediate *APOL1* ROR, Intervention (N=486)**			**Delayed *APOL1* ROR, Control (N=468)**			**Immediate *APOL1* ROR, Intervention (N=194)**			**Delayed *APOL1* ROR, Control (N=183)**		
	Baseline N=486	3-month N=452	6-month N=414	Baseline N=468	3-month N=431	6-month N=397	Baseline N=194	3-month N=181	6-month N=170	Baseline N=183	3-month N=167	6-month N=148
Increase in antihypertensive number		18 (3.9%)	37 (8.9%)		19 (4.4%)	24 (6.0%)		12 (6.6%)	25 (14.7%)		12 (7.2%)	15 (10.1%)
Decrease in antihypertensive number		16 (3.5%)	20 (4.8%)		22 (5.1%)	23 (5.8%)		6 (3.3%)	8 (4.7%)		11 (6.6%)	12 (8.1%)
No change in number of antihypertensives		418 (92.5%)	357 (86.2%)		390 (90.5%)	350 (88.2%)		163 (90.1%)	137 (80.6%)		144 (86.2%)	121 (81.8%)

Data presented as no. (%), unless otherwise indicated.
*p<0.05 (intervention vs. control).
†SGLT2 inhibitors were counted as antihypertensives due to their modest blood pressure lowering effect.
ACE indicates angiotensin converting enzyme; ROR, return of *APOL1* testing results; SD, standard deviation; SGLT2, sodium glucose cotransporter 2.

© 2026 Eadon MT et al. *JAMA Network Open.*

eTable 4. Blood Pressure Outcomes at 3 Months and 6 Months, *APOL1*-HR Population

	**Immediate *APOL1* ROR, Intervention**	**Delayed *APOL1* ROR, Control**	**Difference Between Groups (95% CI)**
**Primary analyses*, Change in SBP, Baseline to 3 months**			
SBP (mm Hg) 3-month<br>All participants, mean±SD	130.4±18.0<br>(n=446)	131.8±19.4<br>(n=429)	
Change SBP (mm Hg) All participants,<br>mean±SD or mean (95% CI)	-1.8±18.8<br>(n=446)	-1.5±17.3<br>(n=428)	-0.3 (-2.7, 2.1)<br>P=0.78
**Covariate adjusted linear regression model of primary outcome†, 3 months**			
Change SBP 3-month, All participants<br>Least-square mean±SE or (95% CI)	-1.3±0.8<br>(n=446)	-0.8±0.8<br>(n=428)	-0.5 (-2.6, 1.6)
**Secondary analysis, Covariate adjusted mixed model analysis of SBP Trend, Baseline to 6 months**			
Trend in SBP 6-month (mm Hg), All participants, Mixed model‡ Least square mean±SE or (95% CI)	130.7±0.7<br>(n=424)	131.8±0.7<br>(n=411)	-1.1 (-2.8, 0.7)
**Per-protocol subgroup analyses, covariate adjusted linear regression model†, 3 months**			
Change SBP 3-month, Uncontrolled baseline BP<br>Least-square mean±SE or (95% CI)	-11.2±1.3<br>(n=178)	-6.80±1.3<br>(n=163)	-4.5 (-8.1, -0.9)
Change SBP 3-month, Uncontrolled baseline BP on antihypertensive therapy<br>Least-square mean±SE or (95% CI)	-11.2±1.3<br>(n=167)	-6.6±1.4<br>(n=152)	-4.7 (-8.4, -0.9)
Change SBP (mm Hg) 3-month, with CKD<br>Least-square mean±SE or (95% CI)	0.1±1.4<br>(n=132)	-0.5±1.3<br>(n=155)	0.5 (-3.1, 4.2)
Change SBP 3-month, Controlled baseline BP<br>Least-square mean±SE or (95% CI)	5.0±1.1<br>(n=268)	2.4±1.0<br>(n=265)	2.7 (-0.2, 5.5)
**Exploratory analyses, Change in SBP, Baseline to 6 months**			
SBP (mm Hg) 6-month<br>All participants, mean±SE	129.5±18.3<br>(n=424)	131.5±18.4<br>(n=411)	
Change SBP 6-month (mm Hg), All participants, Mixed model‡ Least square mean±SE or (95% CI)	-2.5±0.8<br>(n=424)	-1.0±0.8<br>(n=410)	-1.5 (-3.6, 0.7)
Change SBP (mm Hg) Uncontrolled BP, Mixed model‡<br>Least square mean±SE or (95% CI)	-12.0±1.3<br>(n=172)	-10.2±1.4<br>(n=153)	-1.7 (-5.4, 1.9)
Change SBP (mm Hg) Uncontrolled BP on antihypertensive therapy, Mixed model‡ Least square mean±SE or (95% CI)	-11.8±1.4<br>(n=162)	-10.0±1.4<br>(n=144)	-1.8 (-5.6, 2.0)
Change SBP (mm Hg) with CKD, Mixed model‡<br>Least square mean±SE or (95% CI)	-3.7±1.4<br>(n=126)	-0.9±1.2<br>(n=147)	-2.8 (-6.5, 1.0)
**Exploratory analyses, Change in DBP, Baseline to 3 6 months**			
DBP (mm Hg) 3-month<br>All participants, mean±SD	81.1±12.3<br>(n=446)	81.7±13.2<br>(n=429)	
Change DBP (mm Hg) 3-month, All Participants,<br>Least square mean±SE or (95% CI)	-1.5±0.5<br>(n=446)	-0.6±0.5<br>(n=428)	-0.9 (-2.2, 0.5)‡
DBP (mm Hg) 6-month<br>All participants, mean±SD	80.5±12.0<br>(n=424)	81.5±12.5<br>(n=411)	
Change DBP (mm Hg) 6-month, All participants,<br>Least square mean±SE or (95% CI)	-2.2±0.5<br>(n=424)	-0.9±0.5<br>(n=410)	-1.3 (-2.7, 0.1)‡
**Exploratory Analyses§, Change in BP control category (controlled SBP<140 and DBP<90), All Participants**			
Participants with controlled BP at 3 months, no./ no. total (%); Odds ratio of controlled BP (Intervention vs. Control) (95% CI)	288/446 (64.6%)	273/429 (63.6%)	1.07 (0.81, 1.40)

© 2026 Eadon MT et al. *JAMA Network Open.*

Participants with controlled BP at 6 months, no./ no. total (%); Odds ratio of controlled BP (Intervention vs. Control) (95% CI)	276/424 (65.1%)	255/411 (62.0%)	1.18 (0.89, 1.56)
Participants new controlled at 3 months, no./ no. total (%), difference in proportions (95% CI)	86/446 (19.3%)	57/428 (13.3%)	6.0% (1.1%, 10.8%)
Participants stay controlled at 3 months, no./no. total (%), difference in proportions (95% CI)	202/446 (45.3%)	215/428 (50.2%)	-4.9% (-11.6%, 1.7%)
Participants new uncontrolled at 3 months, no./ no. total (%), difference in proportions (95% CI)	66/446 (14.8%)	50/428 (11.7%)	3.1% (-1.4%, 7.6%)
Participants stay uncontrolled at 3 months, no./no. total (%), difference in proportions (95% CI)	92/446 (20.6%)	106/428 (24.8%)	-4.1% (-9.7%, 1.4%)

[*]T-test.
[†]Covariate-adjusted mixed model exploratory analysis.
[‡]Mixed-effect model for repeated measures.
[§]Covariate-adjusted Generalized Estimating Equation (GEE) model analysis.
BP indicates blood pressure; CI, confidence interval; CKD, chronic kidney disease; DBP, diastolic blood pressure; ROR, return of *APOL1* testing results; SBP, systolic blood pressure; SD, standard deviation; SE, standard error.

© 2026 Eadon MT et al. *JAMA Network Open.*

eTable 5. Sensitivity Analysis of Primary Outcome With Removal of EHR-Derived Measures

**Covariate-Adjusted Linear Regression Analysis of Systolic Blood Pressure (mmHg) Change from Baseline to 3 Months (EHR outcomes removed)**	**Immediate *APOL1* Testing, Intervention N=434**	**Delayed *APOL1* Testing, Control N=426**
**Analysis[1] without data imputation:**		
SBP change from baseline to 3 months		
LS mean (SE)	-1.4 (0.82)	-1.2 (0.81)
95% CI	(-3.0, 0.2)	(-2.8, 0.4)
Difference between treatment groups (Intervention vs. Control)		
LS mean (SE)	-0.2 (1.11)	
95% CI	(-2.4, 2.0)	
P-value	0.8606	

NOTE: Data are presented by randomized treatment groups. CI = Confidence Interval, SE = Standard Error, LS mean=Least-square mean.

1. Covariates prespecified in SAP consist of age, sex, baseline CKD, clinical group, and baseline SBP. Baseline CKD is excluded as a covariate due to ≥ 1% missing data.

2. The Per-protocol population excludes participants with known protocol deviations or violations. Those with treatment crossovers are also excluded from the population.

© 2026 Eadon MT et al. *JAMA Network Open.*

eTable 6. Controlled and Uncontrolled Systolic Blood Pressure Analysis

**Covariate-Adjusted Linear Regression Analyses of Systolic Blood Pressures (mmHg) Change from Baseline to 3 Months - For Participants with Controlled and Uncontrolled BP at Baseline**	**Controlled BP at Baseline (SBP < 140 mmHg and DBP < 90 mmHg) N=575**		**Uncontrolled BP at Baseline (SBP ≥ 140 mmHg or DBP ≥ 90 mmHg) N=377**	
	**Immediate *APOL1* Testing, Intervention N=291**	**Delayed *APOL1* Testing, Control N=284**	**Immediate *APOL1* Testing, Intervention N=194**	**Delayed *APOL1* Testing, Control N=183**
**Descriptive statistics for observed data**				
Baseline				
n	291	284	194	183
Mean (SD)	121.7 (11.07)	122.7 (10.54)	148.6 (16.71)	151.1 (17.42)
Median (Q1, Q3)	123.0 (114.0, 130.0)	124.0 (116.5, 130.5)	145.5 (139.0, 156.5)	148.5 (140.5, 160.0)
Min, Max	81.0, 139.5	88.5, 139.5	118.0, 229.0	111.5, 235.5
Month 3				
n	268	265	178	163
Mean (SD)	125.8 (16.28)	124.8 (14.42)	137.3 (18.27)	143.2 (21.04)
Median (Q1, Q3)	124.5 (114.0, 135.5)	124.0 (115.0, 133.0)	136.0 (125.5, 146.5)	141.5 (127.5, 158.0)
Min, Max	82.0, 186.0	83.0, 186.0	96.0, 201.0	100.0, 200.0
Change from baseline to 3 months				
n	268	265	178	163
Mean (SD)	4.5 (15.91)	2.0 (14.19)	-11.4 (18.73)	-7.1 (20.32)
Median (Q1, Q3)	4.5 (-5.5, 12.3)	1.5 (-7.0, 11.0)	-11.0 (-21.0, -1.0)	-7.5 (-18.0, 7.0)
Min, Max	-44.0, 67.5	-36.5, 50.0	-82.5, 55.5	-75.5, 46.5
**Linear regression model analysis result[1]:**				
SBP change from baseline to 3 months				
LS mean (SE)	5.1 (1.06)	2.4 (1.05)	-11.1 (1.29)	-7.0 (1.33)
95% CI	(3.0, 7.2)	(0.3, 4.4)	(-13.6, -8.5)	(-9.6, -4.4)
Difference between treatment groups (Intervention vs. Control)				
LS mean (SE)	2.7 (1.47)		-4.1 (1.84)	
95% CI	(-0.2, 5.6)		(-7.7, -0.5)	
P-value	0.0646		0.0269	
Interaction p-value 0.0039				

Covariates prespecified in SAP consist of age, sex, baseline CKD, and baseline SBP. Baseline CKD is excluded as a covariat e due to ≥ 1% missing data, and baseline SBP is excluded due to its correlation coefficient with baseline blood pressure control status is ≥ 0.6.

© 2026 Eadon MT et al. *JAMA Network Open.*

eTable 7. Secondary Chronic Kidney Disease-Related Outcomes at 6 Months

	Immediate *APOL1* ROR, Intervention (N=486)	Delayed *APOL1* ROR, Control (N=468)	Difference in Proportions (95% CI)
**Documented diagnosis of CKD Stage 3 or above**			
Documented diagnosis of CKD Stage 3 or above at baseline, no./ no. total (%)	77/456 (16.9%)	98/441 (22.2%)	-5.3% (-10.5%, -0.2%)
Documented diagnosis of CKD Stage 3 and above by 6 months, no./ no. total (%)	83/456 (18.2%)	103/441 (23.4%)	-5.2% (-10.5%, 0.2%)
Change in documented diagnosis for CKD Stage 3 and above from baseline to 6 months, no./ no. total (%)	6/456 (1.3%)	5/441 (1.1%)	0.2% (-1.3%, 1.6%)
Change in documented diagnosis for CKD Stage 3 and above from baseline to 6 months, among participants with no diagnosis at baseline, no./ no. total (%)	6/379 (1.6%)	5/343 (1.5%)	0.1% (-1.7%, 1.9%)
**Change in Documentation of CKD, by Baseline CKD**			
With CKD, documented diagnosis of CKD (any stage) at baseline, no./ no. total (%)	109/140 (77.9%)	128/165 (77.6%)	
With CKD, change in documented diagnosis of CKD (any stage) from baseline to 6 months	6/140 (4.3%)	9/165 (5.5%)	OR (95% CI) 0.78 (0.27, 2.27)
Without CKD, change in documented diagnosis of CKD (any stage) from baseline to 6 months	24/308 (7.8%)	1/267 (0.4%)	OR (95% CI) 22.40 (3.01, 166.83)

CKD defined by ICD-10 codes, eGFR and urine albuminuria or proteinuria tests in the 24 months prior to enrollment.
CI indicates confidence interval; CKD, chronic kidney disease; ROR, return of *APOL1* testing results.

eTable 8. Perspectives of Trial Participants on Sharing Genotype Results and Self-Reported Behaviors

	***APOL1*-HR, All Participants (N=954)**	**Immediate *APOL1* ROR, Intervention (N=486)**	**Delayed *APOL1* ROR, Control (N=468)**
Previous Genetic Test, yes no./no. total (%)	96/930 (10.3%)	49/473 (10.4%)	47/457 (10.3%)
Had enough information on joining the study and getting tested, Strongly agree or Agree, no./ no. total (%)	821/856 (95.9%)	426/438 (97.3%)	395/418 (94.5%)
Information for test (or study) was easy to understand, Strongly agree or Agree, no./ no. total (%)	789/857 (92.1%)	384/438 (87.7%)	405/419 (96.7%)
Lifestyle changes, 6-month			
Became more physically active or exercised more, yes no./ no. total (%)	437/807 (54.2%)	229/412 (55.6%)	208/395 (52.7%)
Made healthier changes to diet in past 3 months, yes no./ no. total (%)	484/807 (60.0%)	255/411 (62.0%)	229/396 (57.8%)
**Immediate Testing Group only:**			
If I could go back in time, I would make the same choice to get my APOL1 genetic test result.			
Strongly Agree		243/440 (55.2%)	
Agree		171/440 (38.9%)	
Neither agree nor disagree		10/440 (2.3%)	
Disagree		6/440 (1.4%)	
Strongly disagree		3/440 (0.7%)	
Don't Know		7/440 (1.6%)	

Previous genetic test refers to testing other than for *APOL1*.
ROR indicates return of *APOL1* testing results.

© 2026 Eadon MT et al. *JAMA Network Open.*

eFigure 1. Per-Protocol Subgroup Analyses of Treatment Group Difference in Systolic Blood Pressure Change From Baseline to 3 Months

Subgroup	Treatment Group LS Mean Change from Baseline (95% CI) Intervention	Control	Intervention vs. Control Difference in LS Mean Change from Baseline (95% CI)	P-value for Interaction
**Overall (874)**	-1.33 (-2.84, 0.18)	-0.82 (-2.33, 0.68)	-0.51 (-2.60, 1.59)	
**Uncontrolled BP (>= 140/90 mmHg):**				0.0039
Yes (341)	-11.05 (-13.58, -8.52)	-6.98 (-9.58, -4.37)	-4.07 (-7.68, -0.47)	
No (533)	5.09 (3.02, 7.17)	2.38 (0.32, 4.44)	2.71 (-0.16, 5.59)	
**Uncontrolled BP & HTN Intervention:**				0.0041
Yes (319)	-11.06 (-13.68, -8.44)	-6.78 (-9.49, -4.07)	-4.28 (-8.04, -0.53)	
No (555)	4.52 (2.47, 6.57)	1.91 (-0.13, 3.94)	2.61 (-0.23, 5.45)	
**Baseline CKD:**				0.4248
Yes (287)	0.05 (-2.67, 2.78)	-0.70 (-3.21, 1.81)	0.75 (-2.95, 4.45)	
No (527)	-1.64 (-3.58, 0.29)	-0.52 (-2.58, 1.54)	-1.12 (-3.85, 1.61)	

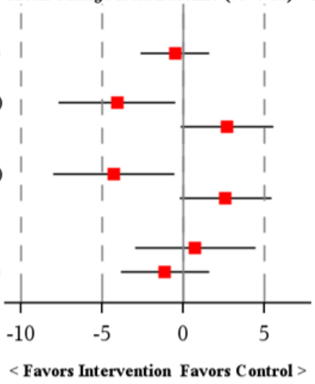

eFigure 1: Per-protocol subgroup analyses. A blood pressure of ≥140/90 means either a systolic reading of 140 mmHg or greater or a diastolic reading of 90 mmHg or greater. The number of participants in each subgroup is based on those included in each subgroup analysis model. Prespecified covariates in the SAP consist of age, sex, baseline CKD, and baseline SBP. Baseline CKD is excluded as a covariate due to ≥ 1% missing data. A covariate that is highly correlated with a subgroup variable is not included in the model.

© 2026 Eadon MT et al. *JAMA Network Open.*

eFigure 2. Exploratory Subgroup Analyses of Treatment Group Difference in Systolic Blood Pressure Change From Baseline to 3 Months

Subgroup	Treatment Group LS Mean Change from Baseline (95% CI) Intervention	Control	Intervention vs. Control Difference in LS Mean Change from Baseline (95% CI)	P-value for Interaction
**Overall (874)**	-1.33 (-2.84, 0.18)	-0.82 (-2.33, 0.68)	-0.51 (-2.60, 1.59)	
**Age:**				0.9074
< Median (421)	-1.65 (-3.78, 0.48)	-1.01 (-3.19, 1.16)	-0.64 (-3.65, 2.38)	
>= Median (453)	-1.03 (-3.11, 1.04)	-0.64 (-2.71, 1.42)	-0.39 (-3.29, 2.51)	
**Sex:**				0.3692
Female (561)	-3.72 (-5.49, -1.95)	-2.49 (-4.42, -0.57)	-1.22 (-3.83, 1.39)	
Male (313)	1.43 (-1.16, 4.02)	0.65 (-1.71, 3.01)	0.78 (-2.72, 4.27)	
**eGFR (mL/min/1.73m2):**				0.4916
<= 60 (236)	-0.16 (-3.16, 2.84)	-1.04 (-3.77, 1.69)	0.88 (-3.17, 4.92)	
Other (630)	-1.63 (-3.39, 0.13)	-0.84 (-2.67, 0.99)	-0.78 (-3.26, 1.69)	
**Verbal Health Literacy:**				0.3618
Low (226)	0.21 (-2.67, 3.10)	2.37 (-0.55, 5.28)	-2.15 (-6.25, 1.94)	
High (648)	-1.91 (-3.65, -0.17)	-1.96 (-3.70, -0.21)	0.05 (-2.37, 2.47)	
**Other Health Literacy:**				0.5553
Low (130)	1.53 (-2.39, 5.44)	3.42 (-0.31, 7.14)	-1.89 (-7.28, 3.51)	
High (734)	-1.75 (-3.39, -0.12)	-1.63 (-3.28, 0.03)	-0.13 (-2.40, 2.15)	
**Education:**				0.1024
<= High school (366)	-0.63 (-2.86, 1.61)	1.95 (-0.36, 4.27)	-2.58 (-5.79, 0.63)	
> High school (504)	-1.92 (-3.92, 0.09)	-2.84 (-4.81, -0.87)	0.93 (-1.81, 3.66)	
**Health Insurance:**				0.9465
Private[1] (308)	-1.92 (-4.49, 0.64)	-1.96 (-4.42, 0.50)	0.04 (-3.48, 3.55)	
Medicare/Medicaid (402)	-1.66 (-3.80, 0.49)	-0.94 (-3.21, 1.33)	-0.72 (-3.80, 2.36)	
Other[2] (160)	0.97 (-2.51, 4.46)	1.64 (-1.82, 5.10)	-0.66 (-5.54, 4.21)	
**Poverty:**				0.0249
Poverty[3] (240)	1.33 (-1.46, 4.12)	-1.58 (-4.44, 1.27)	2.91 (-1.07, 6.89)	
Not in poverty (434)	-4.02 (-6.19, -1.85)	-1.25 (-3.33, 0.83)	-2.77 (-5.74, 0.20)	
**Healthcare Access:**				0.8771
Yes (729)	-1.96 (-3.63, -0.29)	-1.38 (-3.00, 0.25)	-0.58 (-2.87, 1.70)	
No (144)	1.28 (-2.15, 4.70)	2.31 (-1.58, 6.19)	-1.03 (-6.20, 4.13)	
**Previous Genetic Test:**				0.8036
Yes (93)	-2.54 (-6.99, 1.91)	-2.53 (-7.18, 2.13)	-0.01 (-6.43, 6.41)	
No (758)	-1.25 (-2.87, 0.37)	-0.38 (-1.99, 1.23)	-0.87 (-3.12, 1.37)	
**Medication Non-Adherence by VOILS**				0.5603
Non-adherent (340)	-0.17 (-2.54, 2.20)	-0.30 (-2.74, 2.13)	0.13 (-3.20, 3.47)	
Adherent (496)	-1.97 (-3.95, 0.01)	-0.82 (-2.79, 1.15)	-1.15 (-3.90, 1.60)	

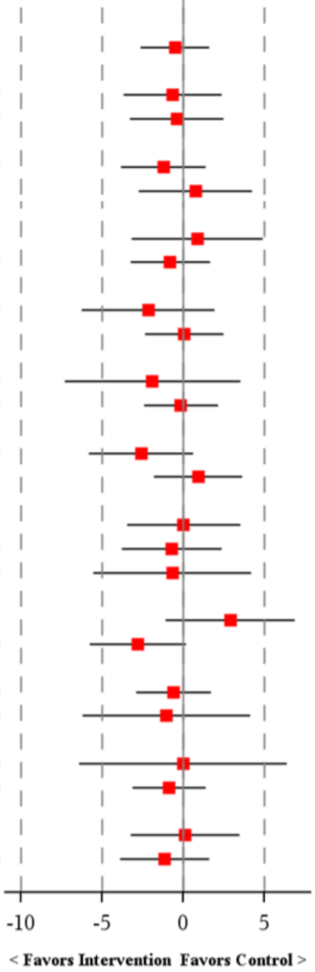

eFigure 2: Exploratory Subgroup analyses. A blood pressure of ≥140/90 means either a systolic reading of 140 mmHg or greater or a diastolic reading of 90 mmHg or greater. Notes: Age subgroups are based on the overall median age of the *APOL1*-HR population. Medication non-adherence by VOILS is assessed using baseline data. Private = Private Health Insurance Only. Other = Other insurance or no insurance. Poverty = In poverty or near poverty per Federal Poverty Guideline 2020. Near poverty = 125% of the federal poverty level. The number of participants in each subgroup is based on those included in each subgroup analysis model. Prespecified covariates in the SAP consist of age, sex, baseline CKD, and baseline SBP. Baseline CKD is excluded as a covariate due to ≥ 1% missing data. A covariate that is highly correlated with a subgroup variable is not included in the model.

© 2026 Eadon MT et al. *JAMA Network Open.*
