# Supplementary material for: Genetic Testing for APOL1 in Adults With Hypertension: The GUARDD-US Randomized Clinical Trial
Source: JAMA Netw Open. 2026 Mar 5;9(3):e260528. doi: 10.1001/jamanetworkopen.2026.0528 (PMC12964156; doi:10.1001/jamanetworkopen.2026.0528)
Supplement: Supplement 3. — Nonauthor Collaborators. Members of the Implementing Genomics in Practice (IGNITE) Pragmatic Trials Network [file jamanetwopen-e260528-s003.pdf]

Supplemental Online Content: Nonauthor Collaborators
*First name, last name, and suffix (if applicable) are required and will appear in PubMed.

*Group Name(s): Implementing Genomics in Practice (IGNITE) Pragmatic Trials Network							
***First Name and Middle Initial(s)**	***Last Name**	***Suffix (eg, Jr, III)**	Academic Degrees	Institution	Location (city, state/province, country)	Role or Contribution, eg, chair, principal investigator	Group (if more than 1 Group listed in the byline) and/or Subgroup (eg, Steering Committee)
Bhargav	Adagarla						
Olanrewaju	Adebayo Olaoye						
Te'Kayla	Alexander						
Meagan	Alley						
Patricia	Allison						
Jose	Alonso						
Kevin	Anstrom						
Irfan	Asif						
Nader	Bahri						
Tala	Basha						
Maria	Bautista						
Travis	Beamon						
Kierra	Bennett						
Domnic	Bett						
Sara	Block						
Emily	Bozant						
Amy M.	Breman						
Christian	Brummett						
Daviana	Buck						
Jocelyn	Bullard						
Jason	Butler						
John Thomas	Callaghan						
Neil	Calman						
Nicole	Canchucaja						
Christina	Carranceja						
Claudia	Cartaya Torres						
Gajapathiraju	Chamarthi						
Ku	Chang						
Tierra	Chavis						
Paige	Chmura						

Supplemental Online Content: Nonauthor Collaborators
*First name, last name, and suffix (if applicable) are required and will appear in PubMed.

*First Name and Middle Initial(s)	*Last Name	*Suffix (eg, Jr, III)	Academic Degrees	Institution	Location (city, state/province, country)	Role or Contribution, eg, chair, principal investigator	Group (if more than 1 Group listed in the byline) and/or Subgroup (eg, Steering Committee)
David	Christian						
Asia	Cobb						
Tyler	Colon						
Porshia	Cook						
Brendon	Cooper						
Zachary	Cowsert						
Brittany	Davis						
Kristine	Daw						
Erin	Delaney						
Lori	Dove						
Erika	Dreidorn						
Stephanie	Eadon						
Elizabeth	Eddy						
Alicia	Ellis						
Stephen	Ellis						
Sylvia	Eluhu						
Ranielle	Emnace						
Bethany	Etheridge						
Edna	Fields						
Aaishah	Francis						
Jyotsna	Fuloria						
Abraham	Garcia						
April	Garrett						
Nancy	Garrett-Mead						
Makenzie	Gee						
Sarah	George						
Kevin	Gernavage						
Daniel	Getu						
Geoff	Ginsburg						
Karla	Giron						
Blake	Goff						

Supplemental Online Content: Nonauthor Collaborators
*First name, last name, and suffix (if applicable) are required and will appear in PubMed.

***First Name and Middle Initial(s)**	***Last Name**	***Suffix (eg, Jr, III)**	Academic Degrees	Institution	Location (city, state/province, country)	Role or Contribution, eg, chair, principal investigator	Group (if more than 1 Group listed in the byline) and/or Subgroup (eg, Steering Committee)
Erika	Gonzalez						
Sarah	Gopman						
Anuksha	Gotmare						
Libertad	Gracia						
Melanie	Gross Hagen						
Samantha	Guagliardo						
Carol	Gutierrez						
Autumn	Hair						
Elizabeth	Harris						
Diane	Hauser						
Alexandria	Henderson						
Shamara	Henry						
Tae'lar	Henry						
Jennelle	Hodge						
Jaclyn	Holland						
Mickey	Holley						
Lynn	Holtam						
Robert	Holtam						
Steven	Houtschilt						
John	Howard						
Sarah	Hutchison						
Ariel Lindsay	Jacobs						
Alia	Jamison						
Judive	Jocelyn						
Danielle	John						
Yashika	Johnson						
Erica	Johnson						
Ross	Jones						
Lydia	Joyner						
Fatoumata	Kaba						
Joseph	Kannry						

Supplemental Online Content: Nonauthor Collaborators
*First name, last name, and suffix (if applicable) are required and will appear in PubMed.

***First Name and Middle Initial(s)**	***Last Name**	***Suffix (eg, Jr, III)**	Academic Degrees	Institution	Location (city, state/province, country)	Role or Contribution, eg, chair, principal investigator	Group (if more than 1 Group listed in the byline) and/or Subgroup (eg, Steering Committee)
Najiba	Khan						
Hwasoon	Kim						
Gabrielle	Kline						
Natalie	Kucher						
Camila Tan	Lam						
Joycelyn	Larbie						
Wai Lang	Lau						
Jeffrey	Leegon						
Kara	Lindsay						
Alexander	Litvintchouk						
Maria	Lopez						
Jared	Lovins						
Chelsey	Lowery						
Jonjerica	Lucky						
Sheng	Luo						
Sheryl	Lynch						
Ebony	Madden						
Emma	Maiman-Stadtmauer						
Ricky	Mareus						
Edlira	Maska						
Mylynda	Massart						
Kelsey	McClara						
Tymia	McNeil						
Sofia	Medina-Pardo						
Shivani	Mehta						
Allan	Mejia						
Peter	Merrill						
Rania	Metry						
Mirta	Milanes						
Nimrit	Mokha						
Anaite	Montes Bu						

Supplemental Online Content: Nonauthor Collaborators
*First name, last name, and suffix (if applicable) are required and will appear in PubMed.

***First Name and Middle Initial(s)**	***Last Name**	***Suffix (eg, Jr, III)**	Academic Degrees	Institution	Location (city, state/province, country)	Role or Contribution, eg, chair, principal investigator	Group (if more than 1 Group listed in the byline) and/or Subgroup (eg, Steering Committee)
Aisha	Montgomery						
Rachel	Myers						
Alexandra	Mykita						
Ying Lu	Nagoshi						
Beth	Nauman						
Arielle	Nelson						
Wambui	Ngari						
Olinda	Nichols						
Thomas D.	Nolin						
Brenda	Olivares						
Johnathan	Oliver						
Henry	Ong						
Jefney	Ongeri						
Frank	Orlando						
Wanda	Parker						
Pauline	Pastore						
Meera	Patel						
Krina	Patel						
Carol	Patterson						
Hadassah	Pegues						
Carol	Pereira						
Kristina	Perez						
Kendra	Phillips						
Sarah	Pleasant						
Brandi	Plunkett						
Jasmine	Powell						
Siddarth	Pratap						
Ebony	Pratt						
Victoria M.	Pratt						
Linda	Prebehalla						
Anthony	Quero						

Supplemental Online Content: Nonauthor Collaborators
*First name, last name, and suffix (if applicable) are required and will appear in PubMed.

***First Name and Middle Initial(s)**	***Last Name**	***Suffix (eg, Jr, III)**	Academic Degrees	Institution	Location (city, state/province, country)	Role or Contribution, eg, chair, principal investigator	Group (if more than 1 Group listed in the byline) and/or Subgroup (eg, Steering Committee)
Teji	Rakhra-Burris						
Jessica	Reinach						
Asa	Revels						
Ryan	Rhoden						
Neatte	Ridgeway						
Chris	Roach						
Joseph	Roberts						
Maria P.	Robles						
Genevieve	Rosier						
Christina	Ross						
Adam	Ruhayel						
Emilie	Ruiz						
Tatiana	Sabin						
Azita	Sadeghpour						
Ismail	Safi						
Ella	Samer						
Siegfried	Schmidt						
Michelle	Sciarrino						
Janet	Seo						
Jennifer	Shepherd						
S. Jawad	Sher						
Nehal	Sheth						
Saskia	Shuman						
Elvira	Silveria Mercado						
Libbie	Silverman						
Arjun D.	Sinha						
Kimberly	Snell						
Jordan	Sonneville						
Mircea	Sorin						
Samantha	St. Elin						
Eric	Stewart						

Supplemental Online Content: Nonauthor Collaborators
*First name, last name, and suffix (if applicable) are required and will appear in PubMed.

***First Name and Middle Initial(s)**	***Last Name**	***Suffix (eg, Jr, III)**	Academic Degrees	Institution	Location (city, state/province, country)	Role or Contribution, eg, chair, principal investigator	Group (if more than 1 Group listed in the byline) and/or Subgroup (eg, Steering Committee)
Anne Marie	Strauss						
Jennifer	Stuart						
Tenira	Stubblefield						
Wayne	Swink						
Evgenia	Teal						
Joshua	Terrell						
Emma	Tillman						
Ana	Tomescu						
Margaret	Trietsch						
Megan	Trietsch						
Cheryl	Tschopp						
Alexis	Turner						
Alexander	Vandeerlin						
Ashley	Vetor						
Kimberly	Vigal						
Emily	Villegas						
Brianne	Voros						
Doris	Webb						
Deanna	Webb						
Jun	Wen						
Sonja	White						
Adeia	Williams						
Lola	Williams						
Precious	Williams						
Sonya	Williams						
Summer	Williamson						
Deanna R.	Willis						
Alex	Woodcock						
Madeline	Young						
Randi	Zinberg						

Supplemental Online Content: Nonauthor Collaborators
*First name, last name, and suffix (if applicable) are required and will appear in PubMed.

***First Name and Middle Initial(s)**	***Last Name**	***Suffix (eg, Jr, III)**	Academic Degrees	Institution	Location (city, state/province, country)	Role or Contribution, eg, chair, principal investigator	Group (if more than 1 Group listed in the byline) and/or Subgroup (eg, Steering Committee)

Supplemental Online Content: Nonauthor Collaborators
*First name, last name, and suffix (if applicable) are required and will appear in PubMed.

***First Name and Middle Initial(s)**	***Last Name**	***Suffix (eg, Jr, III)**	Academic Degrees	Institution	Location (city, state/province, country)	Role or Contribution, eg, chair, principal investigator	Group (if more than 1 Group listed in the byline) and/or Subgroup (eg, Steering Committee)
