# Supplementary material for: Genetic Testing for APOL1 in Adults With Hypertension: The GUARDD-US Randomized Clinical Trial
Source: JAMA Netw Open. 2026 Mar 5;9(3):e260528. doi: 10.1001/jamanetworkopen.2026.0528 (PMC12964156; doi:10.1001/jamanetworkopen.2026.0528)
Supplement: Supplement 4. — Data Sharing Statement [file jamanetwopen-e260528-s004.pdf]

Data Sharing Statement

Eadon. Genetic Testing for APOL1 in Adults With Hypertension. *JAMA Netw Open*. Published March 05, 2026. doi:10.1001/jamanetworkopen.2026.0528

Data
Additional Information: NCT04191824
Data available: Yes
Data types: Deidentified participant data
How to access data: ANVIL
When available: With publication

Supporting Documents
Document types: None

Additional Information
Who can access the data: Those who apply through ANVIL
Types of analyses: For any purpose
Mechanisms of data availability: Via Anvil
Any additional restrictions: None
